# Anti-Inflammatory Activity of Pyrazolo[1,5-*a*]quinazolines

**DOI:** 10.3390/molecules29112421

**Published:** 2024-05-21

**Authors:** Letizia Crocetti, Andrei I. Khlebnikov, Gabriella Guerrini, Igor A. Schepetkin, Fabrizio Melani, Maria Paola Giovannoni, Mark T. Quinn

**Affiliations:** 1 Dipartimento di Neuroscienze, Psicologia, Area del Farmaco e Salute del Bambino (NEUROFARBA), Pharmaceutical and Nutraceutical Section, University of Florence, Via Ugo Schiff 6, 50019 Florence, Italy; letizia.crocetti@unifi.it (L.C.); fabrizio.melani@unifi.it (F.M.); mariapaola.giovannoni@unifi.it (M.P.G.); 2 Kizhner Research Center, National Research Tomsk Polytechnic University, Tomsk 634050, Russia; aikhl@chem.org.ru; 3 Department of Microbiology and Cell Biology, Montana State University, Bozeman, MT 59717, USA; igor@montana.edu

**Keywords:** pyrazolo[1,5-*a*]quinazoline, anti-inflammatory compound, mitogen-activated protein kinase, *c*-Jun *N*-terminal kinase, molecular docking, pharmacophore mapping

## Abstract

Chronic inflammation contributes to a number of diseases. Therefore, control of the inflammatory response is an important therapeutic goal. To identify novel anti-inflammatory compounds, we synthesized and screened a library of 80 pyrazolo[1,5-*a*]quinazoline compounds and related derivatives. Screening of these compounds for their ability to inhibit lipopolysaccharide (LPS)-induced nuclear factor κB (NF-κB) transcriptional activity in human THP-1Blue monocytic cells identified 13 compounds with anti-inflammatory activity (IC_50_ < 50 µM) in a cell-based test system, with two of the most potent being compounds **13i** (5-[(4-sulfamoylbenzyl)oxy]pyrazolo[1,5-*a*]quinazoline-3-carboxamide) and **16** (5-[(4-(methylsulfinyl)benzyloxy]pyrazolo[1,5-*a*]quinazoline-3-carboxamide). Pharmacophore mapping of potential targets predicted that **13i** and **16** may be ligands for three mitogen-activated protein kinases (MAPKs), including extracellular signal-regulated kinase 2 (ERK2), p38α, and *c*-Jun *N*-terminal kinase 3 (JNK3). Indeed, molecular modeling supported that these compounds could effectively bind to ERK2, p38α, and JNK3, with the highest complementarity to JNK3. The key residues of JNK3 important for this binding were identified. Moreover, compounds **13i** and **16** exhibited micromolar binding affinities for JNK1, JNK2, and JNK3. Thus, our results demonstrate the potential for developing lead anti-inflammatory drugs based on the pyrazolo[1,5-*a*]quinazoline and related scaffolds that are targeted toward MAPKs.

## 1. Introduction

Inflammation is an essential process that protects the host from harmful pathogens or irritants and can be acute, lasting for a short period of time, or chronic and lasting much longer [1,2]. Notably, chronic, low-grade inflammation has been shown to contribute to a variety of diseases, including cardiovascular disease [3], cancer [4], type 2 diabetes [5], Alzheimer’s disease [6], arthritis, and many other chronic inflammatory conditions [5]. Thus, it is essential that effective anti-inflammatory therapeutics are developed to help control chronic inflammation and the onset or progression of these diseases [7]. The current therapeutics for treating inflammation generally focus on suppressing, blocking, or inhibiting proinflammatory mediators of inflammation, such as prostaglandins, leukotrienes, and cytokines [8]. While many of these treatments are effective, it is evident that chronic inflammation continues to be a major component associated with the pathogenesis of chronic inflammatory diseases and that new therapeutic interventions with fewer adverse effects need to be developed. Indeed, the pipeline of new anti-inflammatory therapeutics targeting additional pathways other than those being currently targeted is quite limited. Nevertheless, recent work on the development of new resolving mediators has been a success [9,10].

Whether acute or chronic, inflammation involves the activation and/or recruitment of inflammatory leukocytes to sites of infection or injury. The acute inflammatory response is initiated by resident phagocytes, such as macrophages, dendritic cells, and mast cells, but soon results in the recruitment of large numbers of neutrophils, which are the primary leukocytes involved in acute inflammatory responses [11,12]. If the acute response is not resolved, chronic inflammation can occur, which lasts much longer and primarily involves macrophages, as well as lymphocytes and plasma cells, which are able to produce a variety of bioactive inflammatory mediators that can cause cell and tissue damage [13]. Thus, targeting inflammatory responses of leukocytes, such as neutrophils and macrophages, represents a reasonable approach to treating chronic inflammation.

Current anti-inflammatory therapeutics focus mainly on reducing the production or activity of inflammatory eicosanoids or certain cytokines or blocking their receptors, while others can block lymphocyte trafficking into tissues, prevent the binding of monocyte–lymphocyte costimulatory molecules, or reduce the number of circulating B lymphocytes [14,15,16]. In addition, the potential of targeting several biochemical pathways and multiple enzymes involved in inflammation, including neuroinflammation, has been reported [17]. For example, we have synthesized and characterized a number of compounds with anti-inflammatory activity that inhibit mitogen-activated protein kinase (MAPK) pathways, especially the *c*-Jun *N*-terminal kinase (JNK) pathway [18,19]; antagonize *N*-formyl peptide chemotactic receptors (FPRs) [20]; and inhibit human neutrophil elastase [21]. In addition, we have identified a number of pyridazinone-like compounds with anti-inflammatory activity from a large compound library, suggesting that the pyridazinone scaffold could be useful for the development of novel anti-inflammatory therapeutics [22].

**Figure 1:**
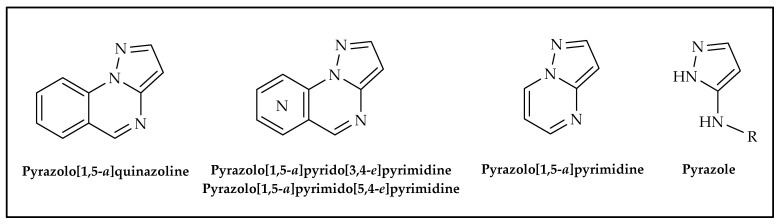
Chemical structures of scaffolds of the screened compounds.

**Figure 2:**
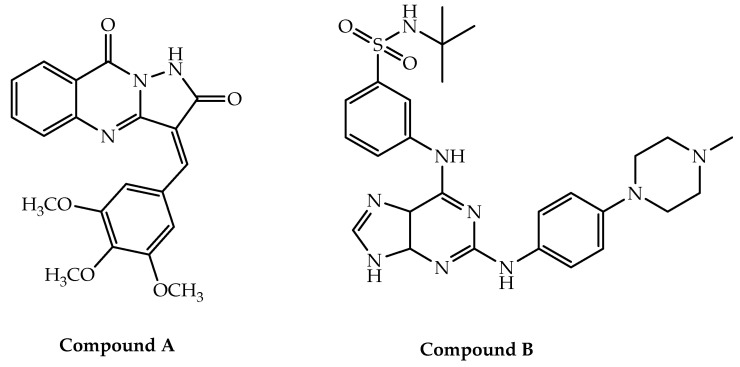
New polyheterocycle anti-inflammatory compounds reported in the literature.

As indicated above, our research group has been investigating a number of biologically active polyheterocycles, and we have created a large library of compounds, including both final products and synthetic intermediates. Here, we selected 80 nitrogen (poly)heterocycles derivatives, which were mainly pyrazolo[1,5-*a*]quinazolines (Figure 1). These compounds were selected based on our previous work in this field, as well as recent publications reporting examples of anti-inflammatory agents with similar nitrogen polycyclic scaffolds [23,24,25,26]. For example, the pyrazolo[5,1-*b*]quinazoline **A** (designated as **3j** in the original publication [23], Figure 2) is a very potent cyclooxygenase 2 (COX-2) inhibitor (IC_50_ = 47 nM), which had about 14-fold selectivity toward COX-2 versus COX-1 but also inhibited 5-lipoxygenase (5-LOX), with an IC_50_ of 2.3 μM. In an in vivo carrageenan-induced paw edema model, 10 mg/kg of compound **A** reduced edema by 39% and did not exhibit gastric ulcerogenic effects. Another recently reported polyheterocyclic anti-inflammatory compound is the purine derivative **B** (designated as **9j** in the original publication [25], Figure 2), which was reported to be a potent dual inhibitor of Janus 2 tyrosine kinase and bromodomain-containing protein 4 (JAK2/BRD4) with IC_50_ values of 22 and 13 nM, respectively, and also downregulated the NF-κB pathway. In vivo studies in an acute ulcerative model demonstrated that 60 mg/kg of compound **B** was able to relieve the symptom of ulcerative colitis with minimal adverse effects.

For the biological screening of the selected library of compounds, we evaluated their effects on lipopolysaccharide (LPS)-induced NF-κB transcriptional activity in THP-1Blue monocyte/macrophages, since NF-κB activation is an important component of many inflammatory responses [27,28]. The complete list and structures of all 80 selected and screened compounds can be found in the Supplementary Materials (Supplementary Tables S1–S3). Two of the most potent compounds identified by our biological screen (**13i** and **16**) were then evaluated in silico to identify potential targets using PharmMapper, which suggested that ERK2, JNK3, and p38α MAPK could be likely biotargets. Furthermore, molecular docking of compounds **13i** and **16** into the binding sites of these kinases using Rosetta docking suggested high affinity binding interactions, confirming that these MAPKs are likely targets for these anti-inflammatory compounds.

## 2. Results and Discussion

### 2.1. Synthesis

**Scheme 1:**
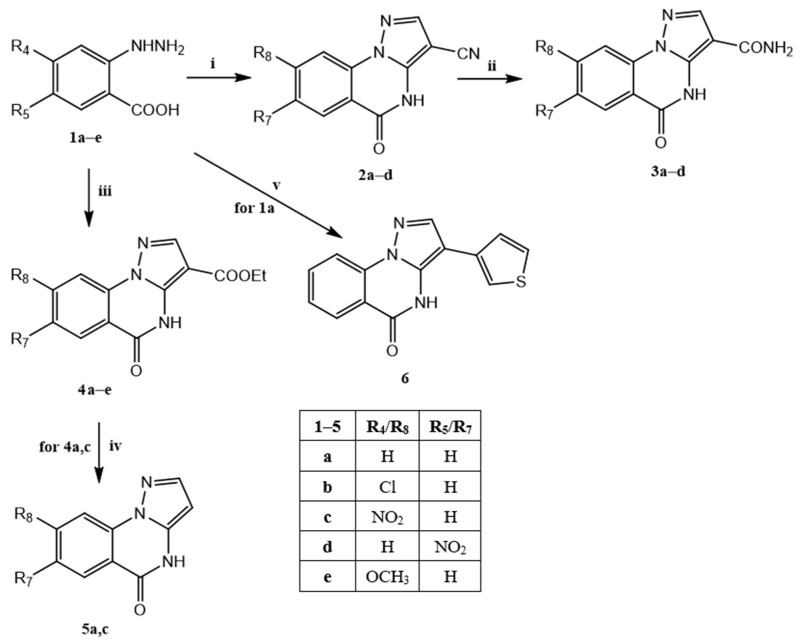
Reagents and conditions: (**i**) (ethoxymethylene)malononitrile, dry DMF, CH_3_COONa, reflux, 2 h; (**ii**) H_2_SO_4_ conc., 80 °C, 2 h; (**iii**) ethyl 2-cyano-3-ethoxyacrylate, dry DMF, CH_3_COONa, reflux, 2 h; (**iv**) HCl 37%, reflux, 4 h; (**v**) 3-oxo-2-(3-thienyl)-propionitrile, glacial CH_3_COOH, reflux, 2 h.

**Scheme 2:**
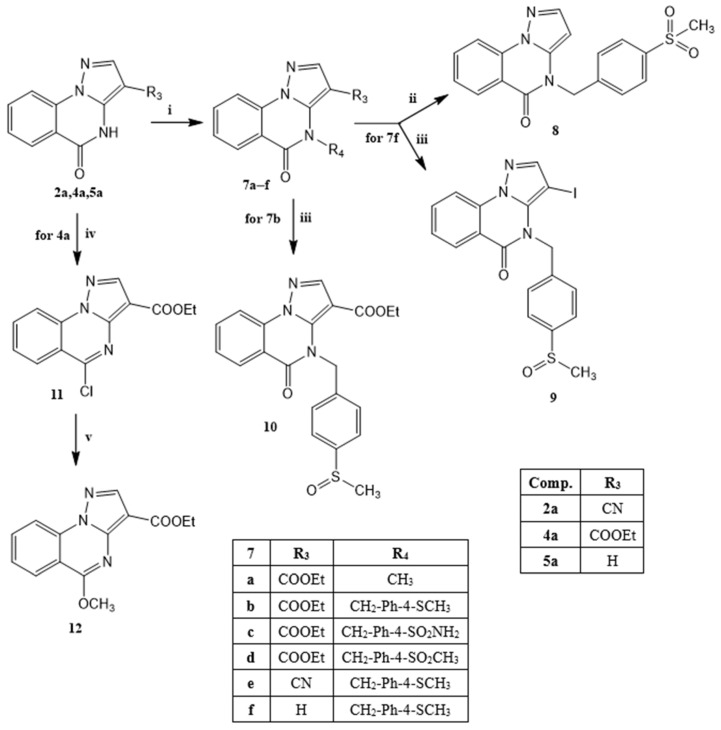
Reagents and conditions: (**i**) **7a**—dry DMF, K_2_CO_3_, CH_3_I, 80 °C, 1 h; **7b**–**f**—dry CH_3_CN, K_2_CO_3_, appropriate 4-substituted benzyl bromide, 80–100 °C, 2–24 h; (**ii**) MeOH, 0 °C, then H_2_O, OXONE^®^, 100 °C, 2 h; (**iii**) acetone/H_2_O (10:1), HIO_3_, TBAB, 5 min, then 80 °C, 2 h; (**iv**) POCl_3_, PCl_5_, 100 °C, 1.5 h; (**v**) MeOH, K_2_CO_3_, reflux, 1.5 h.

**Scheme 3:**
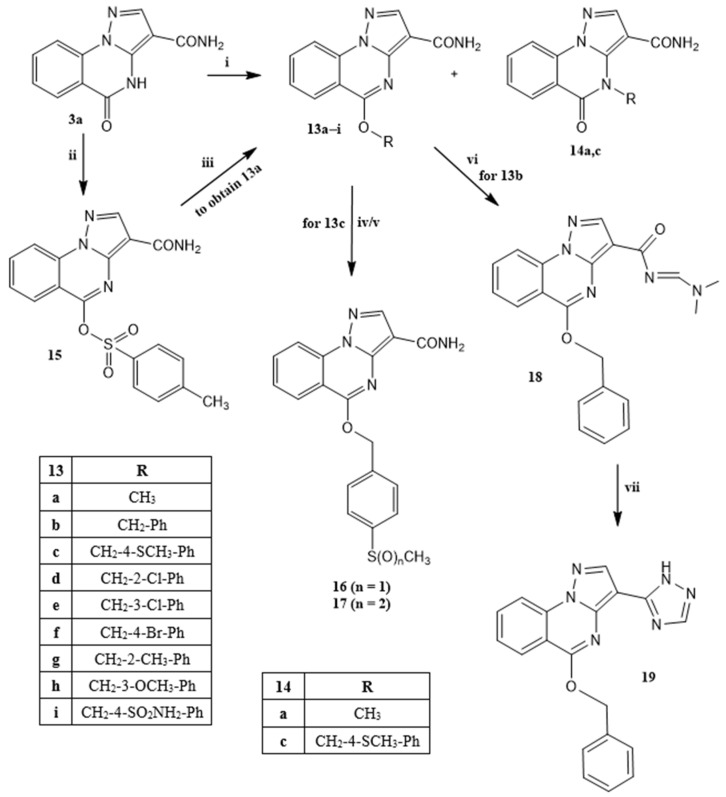
Reagents and conditions: (**i**) **13a/14a**—dry DMF, Cs_2_CO_3_, CH_3_I, 80 °C, 1 h; **13b**–**i** and **14c**—dry DMF, K_2_CO_3_, appropriate benzyl halide, 50 °C, 2 h; (**ii**) dry CH_2_Cl_2_, NEt_3_, tosyl chloride, 150 °C, 3 h; (**iii**) dry DMF, *t*-BuOK, MeOH, reflux, 1.5 h; (**iv**) **16**—acetone/H_2_O (10:1), HIO_3_, TBAB, 5 min, then 80 °C, 2 h; (**v**) **17**—MeOH, 0 °C, then H_2_O, OXONE^®^, 100 °C, 48 h; (**vi**) toluene, DMF-DMA, reflux, 2 h; (**vii**) AcOH, N_2_H_4_·H_2_O, reflux.

**Scheme 4:**
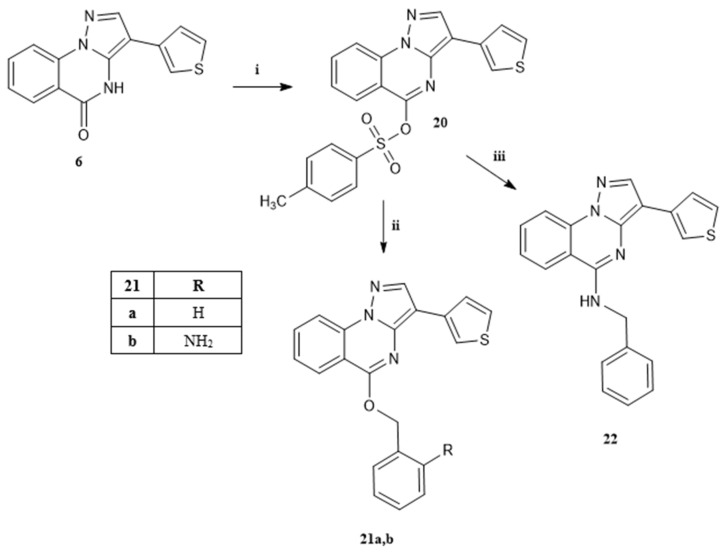
Reagents and conditions: (**i**) 4-toluensulfonylchloride, NEt_3_, CH_2_Cl_2_; (**ii**) DMF/t-BuOK, benzyl alcohol for **21a** and 2-aminobenzyl alcohol for **21b**; (**iii**) DMF, benzylamine.

**Scheme 5:**
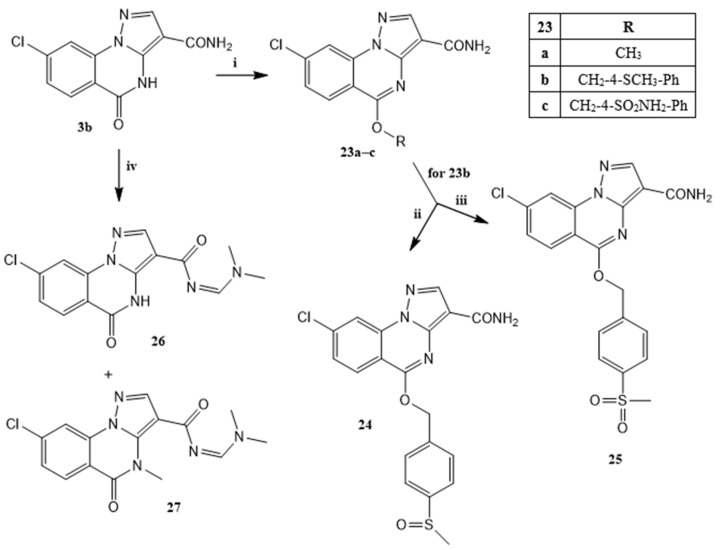
Reagents and conditions: (**i**) Suitable R-halide, dry DMF, K_2_CO_3_, 80 °C, 2 h; (**ii**) acetone/H_2_O (10:1), HIO_3_, TBAB, 5 min, then 80 °C, 2 h; (**iii**) MeOH, 0 °C; then H_2_O, OXONE^®^, 100 °C, 2.5 h; (**iv**) DMF-DMA, dry DMF, dry toluene, reflux, 2 h.

**Scheme 6:**
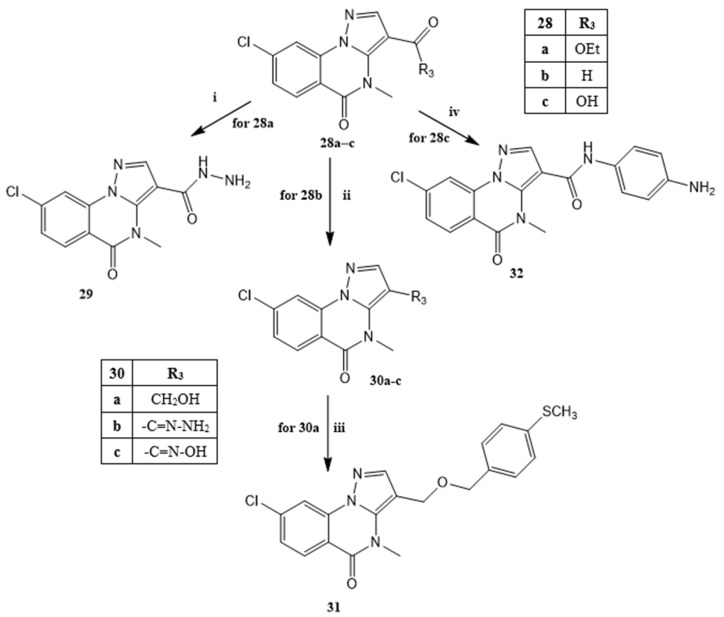
Reagents and conditions: (**i**) NH_2_NH_2_·H_2_O, EtOH 96%, reflux, 7 h; (**ii**) **30a**—MeOH/THF, NaBH_4_, r.t., 20 min; **30b**—NH_2_NH_2_·H_2_O, EtOH 96%, reflux, 1 h; **30c**—H_2_O, NH_2_OH·HCl, 60 °C, 30 min, then NaHCO_3_, 100 °C, 2.5 h; (**iii**) dry CH_3_CN, NaH (60% oil dispersion), r.t., then 4-methylthiobenzyl bromide, 70 °C, 24 h. (**iv**) Step 1: SOCl_2_, reflux, 2h; step 2: benzene-1,4-diamine, dry CH_2_Cl_2_, NEt_3_, reflux, 4 h.

**Scheme 7:**
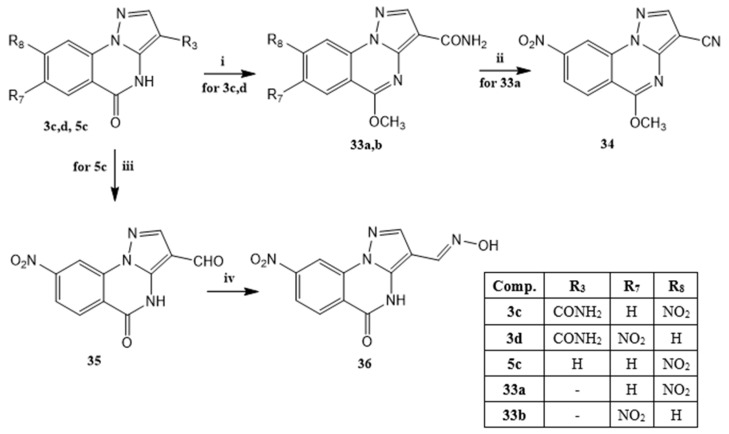
Reagents and conditions: (**i**) CH_3_I, dry DMF, K_2_CO_3_, 80 °C, 2 h (for **33a**) or 5 h (for **33b**); (**ii**) POCl_3_, 80 °C, 2 h; (**iii**) HMTA, glacial CH_3_COOH, reflux, 2 h; (i**v**) NH_2_OH·HCl, NaHCO_3_, H_2_O, 100 °C, 4 h.

**Scheme 8:**
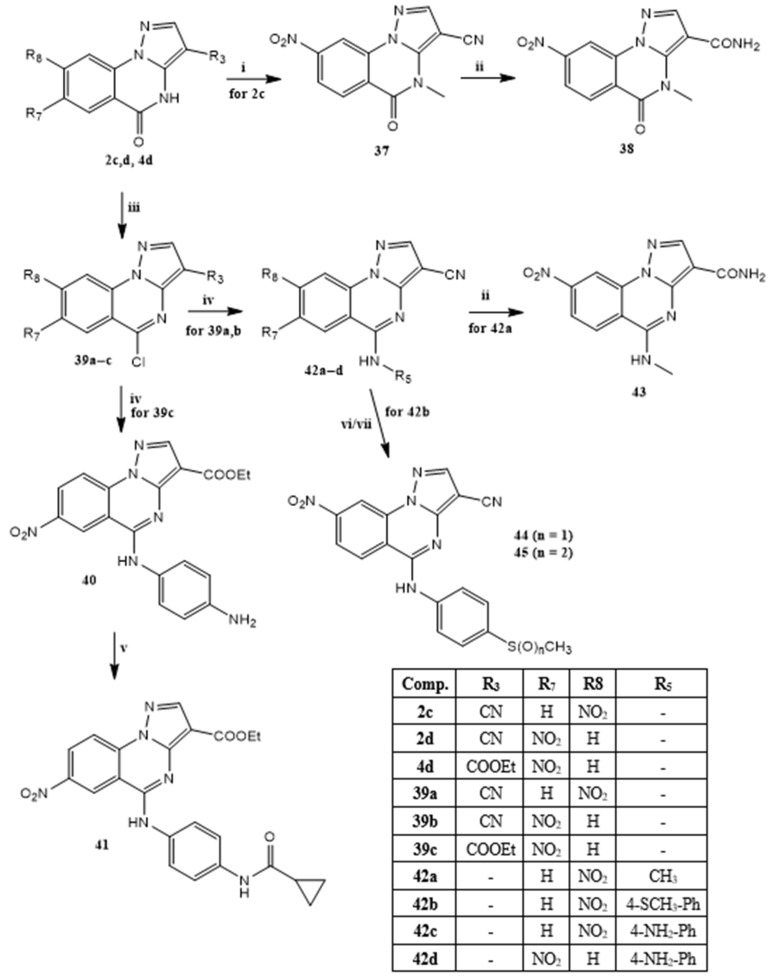
Reagents and conditions: (**i**) CH_3_I, dry DMF, K_2_CO_3_, 80 °C, 2.5 h; (**ii**) H_2_SO_4_ conc., 80 °C, 2 h; (**iii**) POCl_3_, PCl_5_, reflux, 2.5 h (for **39a**, **b**) or 4.5 h (for **39c**); (**iv**) **42a**—CH_3_NH_2_, DIPEA, 1,4-dioxane, r.t., 2 h; **40** and **42b**–**d**—isopropyl alcohol, appropriate aniline, NEt_3_, 2-20 h, reflux; (**v**) cyclopropane carbonyl chloride, dry CH_2_Cl_2_, NEt_3_, 0 °C, 2 h, then r.t., 2h; (**vi**) **43**—acetone/H_2_O (10:1), HIO_3_, TBAB, 5 min; then 80 °C, 40 min; (**vii**) **44**—MeOH, 0 °C, then H_2_O, OXONE^®^, 100 °C, 30 min.

**Scheme 9:**
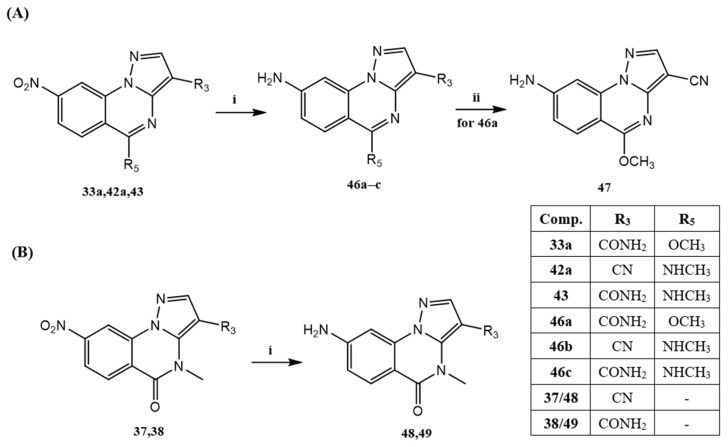
Reagents and conditions: (**A**) Sn, HCl conc., 1 h, r.t.; (**B**) POCl_3_, 80 °C, 2 h.

**Scheme 10:**
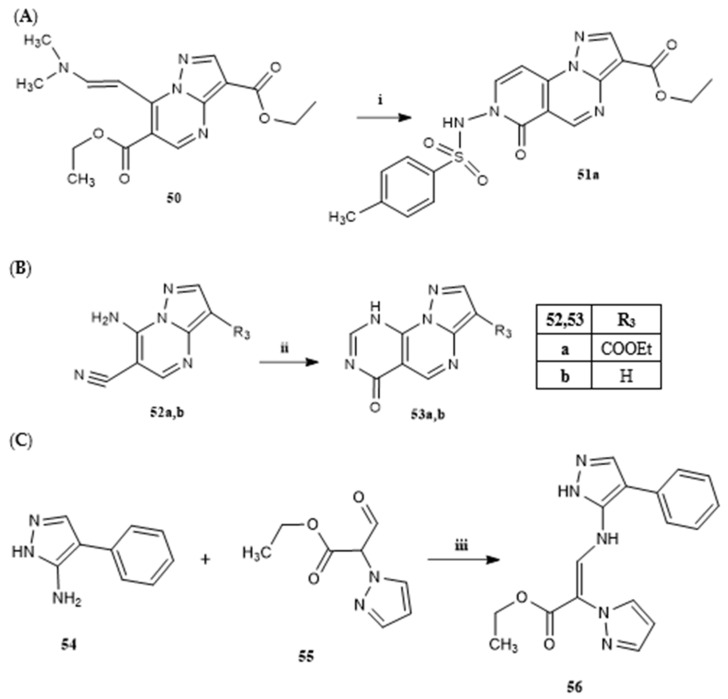
Reagents and conditions: (**A**) 4-tolylsulphonylhydrazide, glacial CH_3_COOH; (**B**) formamide; (**C**) diglyme.

We report here the procedures for the synthesis of 65 new compounds that were included in the screening library. For the 15 compounds in screening library that were already published, we provide the appropriate reference (chemical structures of all compounds and relevant references are presented in Supplementary Tables S1–S3) [29,30,31,32]. As mentioned above, most of the compounds selected for this study are tricycles with a pyrazolo[1,5-*a*]quinazoline scaffold (abbreviated below as PQ), and their synthesis is outlined in Scheme 1, Scheme 2, Scheme 3, Scheme 4, Scheme 5, Scheme 6, Scheme 7, Scheme 8 and Scheme 9. The synthesis of the other compounds with pyrazolo[1,5-*a*]pyrido[3,4*e*]pyrimidine and pyrazolo[1,5-*a*]pyrimido[5,4-*e*]pyrimidine nuclei, as well as the pyrazolo derivatives, is described in Scheme 10. 

Scheme 1 shows the chemical procedure to synthesize the 4,5-dihydropyrazolo[1,5-*a*]quinazoline-5-one (4,5-dihydro-PQ-5-one) scaffold, differently substituted at position 3 and 7 or 8, which was necessary for the synthesis of the compounds shown in the next schemes. The suitable 2-hydrazinobenzoic acid (**1a**–**e**) [33,34,35,36,37] was reacted with ethoxymethylenmalononitrile, ethyl-2-cyano-3-ethoxyacrylate, and 3-oxo-2-(3-thienyl)-proprionitrile to obtain **2a**–**d** (**2a [38]**), **4a**–**e** (**4a**, **b [38,39]**, **4e [40]**), and **6 [41]**, respectively. Compounds **2a**–**d** were transformed into the corresponding 3-carboxamides **3a**–**d** (**3a** [38]) by treatment with sulfuric acid at 80 °C. Alternatively, the 3-ethyl carboxylate derivatives **4a, c** were decarboxylated with concentrated HCl at reflux, resulting in compounds **5a**, **c** (**5a [38]**).

Scheme 2 shows the alkylation of the 4,5-dihydro-PQ-5-one 3-carbonitrile, 3-ethoxycarbonyl, and 3-unsubstituted derivatives (**2a**, **4a**, and **5a**, respectively) in dry DMF or CH_3_CN/K_2_CO_3_/Ar-Br or MeI, which always resulted in the corresponding 4-*N*-alkylated derivatives **7a**–**f** (**7a [34]**). To confirm that alkylation always occurred on the nitrogen at position 4 under these conditions, we synthesized the isomer of **7a** (i.e., the 5-*O*-methyl derivative **12** (ethyl 5-methoxy-PQ-3-carboxylate) [42]). Treatment of **4a** with POCl_3_/PCl_5_ resulted in the 5-chloro derivative **11**, which, in turn, was transformed into the easily recovered **12** with dry DMF/*t*-BuOK/methanol. Through this reaction, we assigned the correct structure to the final compound **7a**. 

Further studies using NMR techniques such as HSQC and HMBC (see Supplementary Figures S60 and S61) were performed on compound **7b**, which again allowed us to correctly assign a structure to the other *N*-alkylated compounds of type **7**. The methylthio group of compounds **7b** and **7f** was oxidized to form the sulfoxide derivatives (-SOMe) with iodic acid/acetone (compounds **9**, **10**), but in the case of the 3-unsubstituted derivative **7f**, even the iodination at position 3 occurred (**9**). Alternatively, the treatment of **7f** with OXONE^®^/water/methanol yielded the corresponding 4-sulfonylmethylbenzyl (-SO_2_Me) derivative **8**. 

In Scheme 2, we show that when COOEt, CN, or H are present in position 3 of the PQ scaffold, the alkylation reactions only yielded the corresponding 4-*N*-alkylates. In contrast, when a CONH_2_ group was present in position 3 (5-oxo-4,5-dihydro-PQ-3-carboxamide), the same reaction (dry DMF/Cs_2_CO_3_/ArBr) was generally regiospecific toward the 5-O position (see Scheme 3). In fact, a mixture of the two regioisomers (5O-R, **13a**, **c** and 4N-R, **14a**, **c**) was achieved only when methyl iodide and 4-methylthiobenzyl bromide were used as alkylating reagents. Even in this case, an in-depth spectroscopic study was performed on compound **14c** to assign the correct structures to the two isomers. The evaluation of the monodimensional and bidimensional spectra (HSQC and HMBC) is included in Supplementary Figures S62 and S63. In all other cases, the 5-*O*-isomer was exclusively obtained (compounds **13b**, **d**–**i**). Additionally, we obtained chemical confirmation of the 5-*O*-methylation in compound **13a** by transforming **3a** into the 5-[4-(methanesulphonyl)phenylmethoxy] derivative **15,** which when treated with methanol/*t*-BuOK, resulting in **13a**. The 5-(4-methylthiobenzyloxy) derivative, **13c**, was oxidized in the presence of iodic acid/acetone or OXONE^®^/methanol, and the two final products (sulfoxide) **16** and (sulfone) **17** were recovered in good yield. Likewise, the 5-benzyloxy derivative **13b** was converted into the *N*-(dimethylamino)methylidene 3-carboxamide **18** (as a mixture of *E* and *Z* isomers, as indicated by TLC) by treating it with DMF-DMA, and the further cyclization to 1,2,4-triazole with hydrazine hydrate in acetic acid resulted in compound **19**.

For the synthesis of the 3-(3-thienyl)-PQ derivatives shown in **Scheme 4**, the starting material was compound **6 [41]**, which was treated with 4-toluensulphonylchloride to obtain the 5-(4-methylbenzene-1-sulphonate derivative **20**. The next reaction with a suitable benzyl alcohol or benzylamine resulted in the final 5-benzyloxy (**21a**, **b**) and 5-benzylamine (**22**) compounds. 

Scheme 5 shows the synthetic route for the final products **24**–**27**, which contain a chlorine atom at position 8 of the PQ scaffold. Starting from **3b**, alkylation in dry DMF/K_2_CO_3_/ArBr or MeI resulted in the final 5-*O*-alkyl derivatives **23a**–**c**. The 5-(4-methylthiobenzyloxy) derivative **23b** was oxidized by iodic acid/acetone or by OXONE^®^, as described above, to obtain the corresponding sulfoxide **24** or sulfone **25**. Compound **3b** was also reacted with DMF-DMA, resulting in a mixture of the *N*-(dimethylamino)methylidene 3-carboxamide derivative **26** and the corresponding 4-*N*-methyl alkylated **27**, which were easily separated by recrystallization.

Starting from the 4-methyl-8-chloro-4,5-dihydro-PQ-5-ones recently published by us and containing ethoxycarbonyl, formyl, and carboxylic groups at position 3, respectively (**28a**–**c**) [39], we further elaborated position 3 of this scaffold, as shown in Scheme 6. In particular, the treatment of compound **28a** (3-COOEt) with hydrazine hydrate in ethanol resulted in the corresponding 3-hydrazone **29**; the 3-formyl derivative **28b** was reduced to the 3-hydroxymethyl derivative **30a** [39]; and treatment of **28b** with hydrazine hydrate or hydroxylamine hydrochloride under suitable conditions resulted in **30b** and **30c**, respectively. The 3-hydroxymethyl group of **30a** [39] was further alkylated with 4-methylthiobenzyl bromide in NaH/CH_3_CN, yielding **31**. Finally, the 3-(4-aminophenyl)carboxamide **32** was synthesized in two steps starting from the 3-carboxylic acid **28c** [39], which was first transformed into the acyl chloride intermediate with SOCl_2_ and then treated with benzene-1,4-diamine in CH_2_Cl_2_/NEt_3_.

Scheme 7 shows the alkylation and reduction reactions of the 8/7-nitro-3-carboxamides (**3c** and **3d**) and of the 8-nitro-3-unsubstituted PQ (**5c**). Compounds **3c**, **d** were alkylated with MeI in DMF/K_2_CO_3_, resulting in the corresponding 5-methoxy compounds **33a**, **b**. Compound **33a** was then dehydrated with POCl_3_ to obtain the 5-methoxy-3-carbonitrile derivative **34**. Alternatively, starting compound **5c** was formylated by HMTA to obtain **35**, which was reacted with hydroxylamine hydrochloride to obtain the corresponding 3-carbaldehyde oxime **36**. 

Scheme 8 shows synthesis of the nitro-derivatives starting from the 8/7-nitro-3-carbonitriles **2c** and **2d** and the 7-nitro-3-ethoxycarbonyl derivative **4d**. Alkylation of **2c** in MeI/DMF/K_2_CO_3_ resulted in the 4-methyl derivative **37**, which was transformed into the corresponding 3-carboxamide **38** with concentrated H_2_SO_4_. The lactams **2c**, **d** and **4d** were also converted into their corresponding 5-chloro-PQ **39a-c** with POCl_3_/PCl_5_, which were then able to undergo a nucleophilic substitution by a suitable amine in *i*-PrOH to obtain the 5-aminoderivatives **40** and **42a**–**d**. Compound **40** was further acylated with cyclopropane carbonyl chloride in CH_2_Cl_2_/NEt_3_ to obtain the final product **41**, while the 8-nitro-5-methylamino-PQ-3-carbonitrile **42a** and the 8-nitro-5-(4-methylthiophenyl)amino-PQ-3-carbonitrile **42b** were transformed into the corresponding 3-carboxamide **43** and the sulfoxide/sulfone derivatives **44** and **45**, respectively. 

Some of the 8-nitroderivatives were transformed into the corresponding 8-amino-PQ compounds (see Scheme 9). Specifically, compounds **33a**, **42a**, **43**, **37**, and **38** were subjected to chemical reduction (Sn/HCl conc.), resulting in the 8-aminoderivatives **46a**–**c**, **48**, and **49**. The 3-carboxamide group of compound **46a** was also dehydrated in POCl_3_ to obtain the 8-amino-5-methoxy-PQ-3-carbonitrile **47**.

Lastly, **Scheme 10** shows the synthesis of compounds **51a**, **53a**, **b**, and **56**, each exhibiting different scaffolds. The pyrazolo[1,5-*a*]pyrimidine **50 [43]** was reacted with 4-tolylhydrazide in glacial acetic acid to obtain the 7-[(4-methylphenyl)sulphonamido]pyrazolo[1,5-*a*]pyrido[3,4-*e*]pyrimidine **51a**. The 6-cyano-7-aminopyrazolo[1,5-*a*]pyrimidines 3-ethoxycarbonyl **52a** or 3-usubstituted **52b** (commercially available) were cyclized with formamide to form the pyrazolo[1,5-*a*]pyrimido[5,4-*e*]pyrimidine scaffold containing an ethoxycarbonyl group or hydrogen at position 3, respectively, to form **53a, b**. Finally, compound **56** was obtained starting from 3-amino-4-phenylpyrazole **54** and ethyl 2-(pyrazol-1′-yl)-2-formylacetate **55** [44] and stopping the reaction before the pyrazolopyrimidine core closure.

### 2.2. Biological Activity

**Figure 3:**
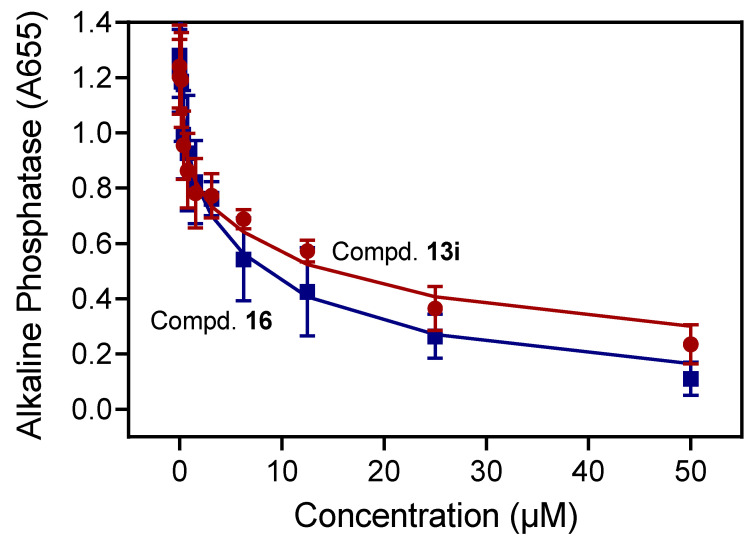
Effect of compounds **13i** and **16** on NF-κB/AP-1 activity. THP-1Blue cells were pretreated with the indicated concentrations of compounds **13i** (red symbols) and **16** (blue symbols) or DMSO control for 30 min, followed by the addition of 250 ng/mL of LPS or buffer for 24 h. NF-κB/AP-1 activity was monitored by measuring secreted alkaline phosphatase activity spectrophotometrically in the cell supernatants (absorbance at 655 nm). The data in each panel are presented as the mean ± S.D. of triplicate samples from one experiment that is representative of three independent experiments.

All compounds (see complete list in Supplementary Tables S1–S3) were screened for their ability to inhibit NF-κB/AP-1 reporter activity in THP-1Blue cells, which is a measure of their anti-inflammatory activity since this pathway is essential to the inflammatory response [45,46]. Although we evaluated a considerable number of compounds, only 13 were able to inhibit NF-κB/AP-1 activity with IC_50_ values <50 μM, and they are shown in Table 1. As an example, the dose-dependent inhibition of LPS-induced NF-κB/AP-1 reporter activity by compounds **13i** and **16** is shown in Figure 3.

Although no true structure–activity relationships could be identified, it was nevertheless possible to make general observations on some structural aspects of these compounds that seem important for activity. All compounds exhibiting some inhibitory activity in THP-1Blue cells were pyrazolo[1,5-*a*]quinazolines, while all the other tricyclics, bicyclics, and pyrazole derivatives tested were completely inactive, indicating that the pyrazolo[1,5-*a*]quinazoline scaffold was the only appropriate structure among those that we tested. Moreover, it seems that among the three possible forms of the pyrazolo[1,5-*a*]quinazoline scaffold, the heteroaromatic form was most effective, as the 5-oxo-4,5-dihydropyrazolo[1,5-*a*]quinazoline (**26**) and 4,5-dihydropyrazolo[1,5-*a*]quinazoline (**58c**) [47] nuclei were much less potent (IC_50_ = 49.3 and 39.1 µM, respectively). Indeed, the heteroaromatic scaffold was present in eleven compounds exhibiting anti-inflammatory activity (IC_50_ = 4.8-30.1 µM). For these products, the primary amide group at position 3 of the heteroaromatic scaffold appeared to be the best, although moderate activity was retained even when a CN group (compound **42a**) or a 3-thienyl (compound **20**) were present. On the other hand, ester or very bulky groups were not compatible with this biological activity. Regarding the substituent bonded to the oxygen at position 5, an increased size of the substituent was favorable for activity (e.g., **13a** versus **13b** with IC_50_ = 24.4 and 4.81 µM, respectively), while the insertion of a chlorine atom or a nitro group at R7/R8 resulted in the maintenance of or a slight increase in activity.

### 2.3. Identification of Potential Protein Targets for Compounds **13i** and **16**

We selected two of the most potent compounds for further characterization. To identify potential protein targets for **13i** and **16**, we performed reverse-pharmacophore mapping on the molecular structures of these compounds. PharmMapper compared a database of pharmacophore patterns with these compounds and generated target information, such as pharmacophoric characteristics and normalized fitness scores. The chemical structures of the compounds were submitted to the PharmMapper server, as mapping explicitly accounts for the three-dimensional structure of a molecule. The 30 top-ranked potential targets found by PharmMapper are shown in Supplementary Table S4 and only the kinase targets are shown in Table 2, as the PharmMapper analysis indicated that three MAPKs (ERK2, p38α MAPK, and JNK1/3) were among the potential targets for compounds **13i** and **16**. Indeed, MAPK signaling plays an important role in phagocyte/macrophage signal transduction cascades [48], and studies have shown that JNK and the p38 MAPK families of proteins are activated in response to phagocyte/macrophage priming/activation (reviewed in [49]). 

### 2.4. Molecular Docking

According to the PharmMapper results, ERK2, JNK3, and p38α MAPK were among the potential biotargets for the investigated compounds. Thus, we performed a more sophisticated docking study of compounds **13i** and **16** into the binding sites of these kinases using the ROSIE web server [50,51,52]. In the PharmMapper database, the retrieved enzymes are represented by the PDB structures 1PME (ERK2), 1PMV (JNK3), and 1W7H (p38α). However, the 1PME structure corresponds to a mutant of ERK2 [53]. Therefore, we used the non-mutated ERK2 (PDB code: 1TVO [54]) for the docking computations. Importantly, the Rosetta docking methodology implemented in ROSIE accounts for the flexibility of the side chains and backbone of the protein in the vicinity of the docked ligand.

**Figure 4:**
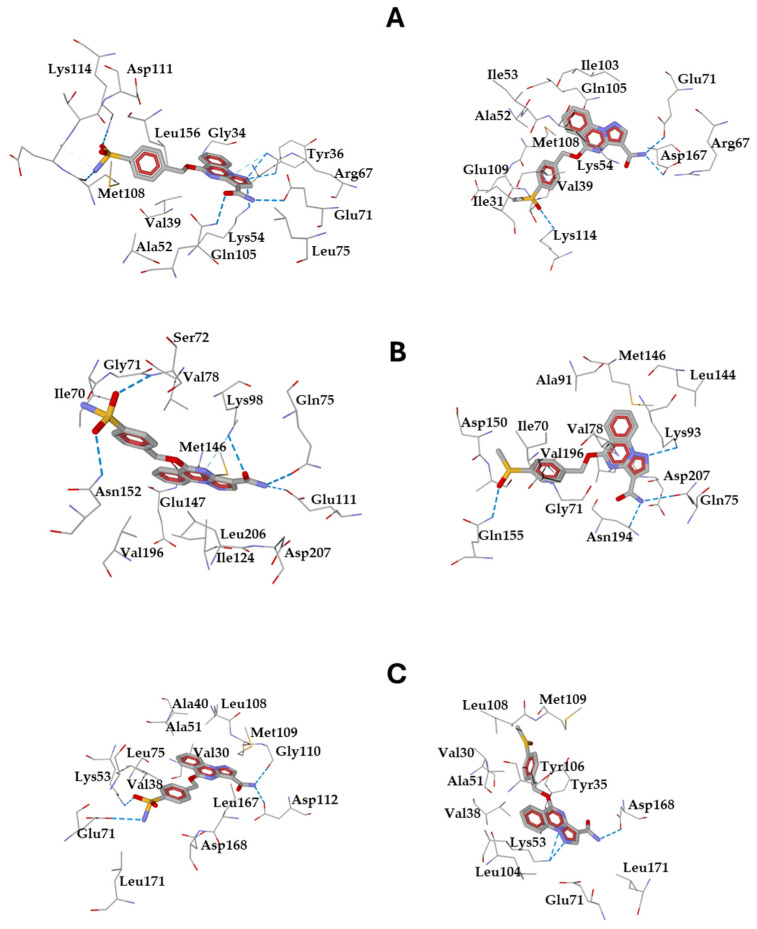
The docking poses of compounds **13i** (**left** images) and **16** (**right** images) in the binding sites of kinases ERK2 (PDB: 1TVO) (Panel (**A**)), JNK3 (PDB: 1PMV) (Panel (**B**)), and p38α (PDB: 1W7H) (Panel (**C**)). The amino acid residues within 3 Å from each pose are visible. H bonds are shown as blue dashed lines.

The docking poses obtained with the lowest interface energies in the binding sites of the kinases for molecules **13i** and **16** are shown in Figure 4. Notably, compounds **13i** and **16** are anchored to the enzymes by a number of H bonds formed with the participation of different functional groups of the ligands (Table 3).

The H-bonding patterns of molecules **13i** and **16** have similar structural features. Thus, protonated lysine residues contained in the binding sites of the investigated kinases (Lys114 in ERK2, Lys93 in JNK3, and Lys53 in p38α) form strong H bonds with the heteroatoms in both ligands. In the case of ERK2 and p38α, the deprotonated Glu71 and neutral Gln75, respectively, participate in H bonding with both **13i** and **16**. It should be noted that the sulfonamide moiety of molecule **13i** and the sulfoxide group of **16** are H bonded to ERK2 and JNK3. The sulfonamide group of **13i** also forms H bonds with p38α (Figure 3). These interactions promote the binding of the ligands to the kinases. The interface energy scores for the docking poses obtained for molecules **13i** and **16** are presented in Table 3. The noticeably negative values of the interface energy scores indicate high affinities of these compounds to ERK2, JNK3, and p38α in accordance with the predictions of PharmMapper (see above). Nevertheless, the interaction of molecules **13i** and **16** with JNK3, according to the ROSIE docking results, should be more prominent than with the other two kinases, in spite of the higher ranking of ERK2 and p38α in the more approximate PharmMapper data. 

In addition to the 1PMV structure of JNK3 retrieved by PharmMapper that was used for the comparative docking with different kinases on the ROSIE server, there is another, more recent structure of JNK3 complexed with a pyrazole-containing ligand in the Protein Data Bank (PDB: 4WHZ [55]). Therefore, this structure was also used for docking molecules **13i** and **16**. For this purpose, the compounds were inserted into the active site with the AUTODOCK 4.1 program, and the conformation with the most favorable binding energy was selected for each complex. The JNK3–inhibitor complexes were further subject to full geometry optimization with the amber99sb force-field. The results were analyzed with a focus on H-bonding interactions (see Supplementary Table S5). It should be noted that Lys93 was found to be involved in H-bonding interactions with the investigated inhibitors using the 4WHZ protein structure, analogous to the results obtained with the 1PMV biotarget (Table 3). Additionally, compounds **13i** and **16** have common H-bonding patterns with these two biotargets (Gln75, Lys93, and Asn152 for ligand **13i**; Gln75, Lys93, and Asn194 for ligand **16**).

### 2.5. Affinity of **13i** and **16** for JNK1-3

To confirm the effectiveness of the predictions based on molecular modeling, compounds **13i** and **16** were evaluated for their ability to bind to JNK1-3 using the KINOMEscan ATP site-dependent binding assay, which reflects the biologically relevant behavior of protein kinases [56]. We found that both compounds bound to JNK1, JNK2, and JNK3, with *K*_d_ values in the micromolar range (Table 4). Although compound **13b** exhibited relatively high activity in THP-1Blue cells (IC_50_ = 4.8 µM), this compound had a low solubility in DMSO and was not able to be tested in the binding assay.

## 3. Materials and Methods

All compound melting points were determined on a Büchi apparatus (New Castle, DE, USA) and are uncorrected. Extracts were dried over Na_2_SO_4_, and the solvents were removed under reduced pressure. Merck F-254 commercial plates (Merck, Durham, NC, USA) were used for analytical TLC to follow the course of the reactions. Silica gel 60 (Merck 70-230 mesh, Merck, Durham, NC, USA) was used for column chromatography. ^1^H-NMR, ^13^C-NMR, HSQC, and HMBC spectra were recorded on an Avance 400 instrument (Bruker Biospin Version 002 with SGU, Bruker Inc., Billerica, MA, USA). Chemical shifts (d) are given in parts per million (ppm), approximated to the nearest 0.01 ppm using the solvent as the internal standard. Coupling constants (*J*) are in Hz and were calculated by Top Spin 3.1 and approximated to 0.1 Hz. Data are reported as follows: chemical shift, multiplicity (exch, exchange; br, broad; s, singlet; d, doublet; t, triplet; q, quartet; m, multiplet; or a combination of those, e.g., dd), integral, assignments, and coupling constant. Mass spectra (*m*/*z*) were recorded on a Varian 1200L ESI-MS triple quadrupole (Varian Inc., Walnut Creek, CA, USA) system in positive ion mode by injecting a 10 mg/L solution of each analyte dissolved in a mixture of mQ H_2_O/acetonitrile 1:1 *v*/*v*. All new compounds exhibited a purity >95%. Microanalyses indicated by the element symbols were performed with a Perkin-Elmer 260 elemental analyzer (Perkin-Elmer, Waltham, MA, USA) for C, H, and N, and they were within ±0.4% of the theoretical values.

### 3.1. Chemistry

Below are the synthetic procedures used to synthesize the active compounds reported in Table 1 (i.e., **13a**, **b**, **i**, **16**, **20**, **23a**, **c**, **26**, **33a**, **b**, **42a**, **43**, **58c**).

#### 3.1.1. 5-Methoxypyrazolo[1,5-*a*]quinazoline-3-carboxamide (**13a**)

A suspension of 4,5-dihydro-5-oxo-pyrazolo[1,5-*a*]quinazoline-3-carboxyamide **3a** [57] (80 mg or 0.35 mmol in 2.5 mL of anhydrous DMF and 0.35 mmol of anhydrous Cs_2_CO_3_) was incubated at room temperature for 15 min. Methyl iodide (0.70 mmol) was added, and the reaction was heated to 80 °C for 1 h. After cooling, 20 mL of ice-cold water was added, and the precipitate formed was recovered by vacuum filtration to obtain the *O*-alkylated compound 13a. This compound was also obtained starting from 15 (see below). Yield 80%, mp 246–247 °C (i-PrOH); TLC eluent: toluene/ethyl acetate/methanol8/2/1.5 *v*/*v*/*v*. ^1^H-NMR (400 MHz, DMSO-d_6_) δ 4.21 (s, 3H, OCH_3_); 7.31 (exch br s, 1H, CONH_2_); 7.48 (exch br s, 1H, CONH_2_); 7.66 (t, 1H, H_7_, *J* = 7.2 Hz); 8.04 (t, 1H, H_8_, *J* = 7.2 Hz); 8.19 (d, 1H, H_9_, *J* = 8.0 Hz); 8.31 (m, 2H, H_2_, H_6_). ^13^C-NMR (100 MHz, DMSO-d_6_) δ 55.5; 106.5; 111.7; 115.0; 126.0; 126.6; 135.9; 137.0; 142.1; 143.9; 160.6; 163.4. ESI-MS calcd for C_12_H_10_N_4_O_2_, 242.24; found: *m*/*z* 243.08 [M + H]^+^. Anal. calcd for C_12_H_10_N_4_O_2_ (C, H, N): C, 59.50; H, 4.16; N, 23.13; found: C, 59.74; H, 4.18; N, 23.22.

#### 3.1.2. General Procedure for Synthesizing Compounds **13b**, **i**

A suspension of 4,5-dihydro-5-oxo-pyrazolo[1,5-*a*]quinazoline-3-carboxyamide **3a** [57] (150 mg or 0.66 mmol in 3.0 mL of anhydrous DMF and 2.64 mmol anhydrous K_2_CO_3_) was incubated at room temperature for 15 min. The appropriate substituted benzyl halide (0.99 mmol) was then added, and the reaction was heated at 50 °C for 2 h. After cooling, 20 mL of ice-cold water was added, and the precipitate formed was recovered by vacuum filtration and washed first with water, then ethanol, and finally with diethyl ether to obtain the desired compounds, which were purified by crystallization from the suitable solvent.

#### 3.1.3. 5-(Benzyloxy)pyrazolo[1,5-*a*]quinazoline-3-carboxamide (**13b**)

From **3a** and benzyl bromide. Yield 65%, mp 208–209 °C (EtOH); IR (nujol) cm^−1^: 3450, 3420, 1676, 1308; TLC eluent: toluene/ethyl acetate/acetic acid 8/2/1.5 *v*/*v*/*v*. ^1^H-NMR (400 MHz, DMSO-d_6_) δ 5.72 (s, 2H, OCH_2_); 7.31 (exch br s, 1H, NH); 7.45 (m, 4H, Ar + NH); 7.60 (d, 2H, Ar, *J* = 8.0 Hz); 7.68 (t, 1H, H_8_, *J* = 8.0 Hz); 8.07 (t, 1H, H_7_, *J* = 8.0 Hz); 8.25 (d, 1H, H_6_, *J* = 8.4 Hz); 8.34 (s, 1H, H_2_); 8.38 (d, 1H, H_9_, *J* = 8.4 Hz). ESI-MS calcd for C_18_H_14_N_4_O_2_, 318.34; found: *m*/*z* 319.12 [M + H]^+^. Anal. calcd for C_18_H_14_N_4_O_2_ (C, H, N): C, 67.92; H, 4.43; N, 17.60; found: C, 67.65; H, 4.41; N, 17.53.

#### 3.1.4. 5-[(4-Sulfamoylbenzyl)oxy]pyrazolo[1,5-*a*]quinazoline-3-carboxamide (**13i**)

Compound **3a** was treated with 4-(bromomethyl)benzenesulphonamide. Yield 90%, mp 167–168 °C (EtOH); TLC eluent: toluene/ethyl acetate/acetic acid 8/2/1 *v*/*v*/*v*. ^1^H-NMR (400 MHz, DMSO-d_6_) δ 5.81 (s, 2H, OCH_2_); 7.27 (exch br s, 1H, CONH_2_); 7.39 (exch br s, 2H, SO_2_NH_2_); 7.46 (exch br s, 1H, CONH_2_); 7.67 (t, 1H, H_7_, *J* = 7.6 Hz); 7.77 (d, 2H, H_2′_, H_6′_, *J* = 8.0 Hz); 7.86 (d, 2H, H_3′_, H_5′_, *J* = 8.4 Hz); 8.06 (t, 1H, H_8_, *J* = 7.6 Hz); 8.27 (d, 1H, H_9_, *J* = 8.0 Hz); 8.33 (d, 1H, H_6_, *J* = 8.0 Hz); 8.33 (s, 1H, H_2_). ^13^C-NMR (100 MHz, DMSO-d_6_) δ 68.7; 106.0; 111.7; 115.1; 126.2; 126.4; 127.0; 128.6; 136.3; 137.0; 140.5; 142.1; 143.7; 143.9; 160.0; 163.8. ESI-MS calcd for C_18_H_15_N_5_O_4_S, 397.41; found: *m*/*z* 398.09 [M + H]^+^. Anal. calcd for C_18_H_15_N_5_O_4_S (C, H, N): C, 54.40; H, 3.80; N, 17.62; found: C, 54.61; H, 3.81; N, 17.69.

#### 3.1.5. 5-[(4-(Methylsulfinyl)benzyloxy]pyrazolo[1,5-*a*]quinazoline-3-carboxamide (**16**)

To a solution of 176 mg (1 mmol) of HIO_3_ in 11 mL of acetone/H_2_O (10:1), a small amount of tetra-n-butylammonium bromide (TBAB) was added while stirring for 5 min. Then, 0.25 mmol of compound **13c** (the synthesis of **13c** is reported in Supplementary Materials) was added, and the mixture was stirred at 80 °C for 2 h. The reaction mixture was cooled, 10 mL of H_2_O was added, and the precipitate formed was collected by vacuum filtration. Yield 88%, mp 225–226 °C (EtOH 80%); TLC eluent: toluene/ethyl acetate/acetic acid 8/2/1 *v*/*v*/*v*. ^1^H-NMR (400 MHz, DMSO-d_6_) δ 2.73 (s, 3H, SOCH_3_); 5.77 (s, 2H, OCH_2_); 7.26 (exch brs, 1H, CONH_2_); 7.46 (exch brs, 1H, CONH_2_); 7.66 (t, 1H, H_7_, *J* = 7.2 Hz); 7.71 (d, 2H, H_2′_, H_6′_, *J* = 7.6 Hz); 7.77 (d, 2H, H_3′_, H_5′_, *J* = 7.6 Hz); 8.04 (t, 1H, H_8_, *J* = 7.2 Hz); 8.25 (d, 1H, H_9_, *J* = 7.6 Hz); 8.30 (m, 2H, H_6_, H_2_). ^13^C-NMR (100 MHz, DMSO-d_6_) δ 68.9; 106,6; 111.6; 115.0; 124.3; 126.1; 126.7; 129.1; 136.0; 137.1; 139.1; 141.9; 144.0; 146.7; 159.8; 163.3. ESI-MS calcd for C_19_H_16_N_4_O_3_S, 380.42; found: *m*/*z* 381.10 [M + H]^+^. Anal. calcd for C_19_H_16_N_4_O_3_S (C, H, N): C, 59.99; H, 4.24; N, 14.73; found: C, 59.75; H, 4.22; N, 14.67.

#### 3.1.6. 5-{[4-(Methanesulfonyl)phenyl]methoxy}-3-(thiophen-3-yl)pyrazolo[1,5-*a*]quinazoline (**20**)

Compound **6** [3-(thiophen-3-yl)pyrazolo[1,5-*a*]quinazolin-5(4*H*)-one] [41] (0.31 mmol) was suspended in 10 mL of methylene chloride and 0.70 mmol of 4-toluenesulfonyl chloride, and 0.6 mL (in excess) of triethylamine was added. The reaction was maintained at reflux temperature for 2–3 h, and then the solvent was evaporated to dryness. The residue was dissolved in isopropyl alcohol and crystallized with the same solvent. Yield 97%, mp 203 °C; TLC eluent: toluene/ethyl acetate/acetic acid 8/2/1.5 *v*/*v*/*v*. ^1^H-NMR (400 MHz, DMSO-d_6_) δ 2.45 (s, 3H, CH_3_); 7.43 (d, 1H, H_2_ thiophene, *J* = 2.8 Hz); 7.45 (d, 1H, H_4_ thiophene, *J* = 5.2 Hz); 7.53 (d, 2H, H_3′_ + H_5′_ *J* = 8.0 Hz); 7.60 (dd, 1H, H_5_ thiophene, *J* = 2.8 Hz; *J* = 5.2 Hz); 7.71 (m, 2H, H_7_ + H_9_); 8.04 (d, 2H, H_2′_ and H_6′_, *J* = 8.0 Hz); 8.08 (m, 1H, thiophene); 8.13 (t, 1H, H_7_, *J* = 8.0 Hz); 8.37 (d, 1H, H_6_, *J* = 8.4 Hz); 8.60 (s, 1H, H_2_). ESI-MS calcd for C_21_H_15_N_3_O_3_S_2_, 421.49; found: *m*/*z* 422.06 [M + H]^+^. Anal. calcd for C_21_H_15_N_3_O_3_S_2_ (C, H, N): C, 59.84; H, 3.59; N, 9.97; found: C, 59.60; H, 3.57; N, 9.93.

#### 3.1.7. General Procedure for Synthesizing Compounds **23a**, **c**

A suspension of 8-chloro-5-oxo-4,5-dihydropyrazolo[1,5-*a*]quinazoline-3-carboxyamide **3b** (the synthesis of **3b** is reported in Supplementary Materials) (150 mg or 0.66 mmol in 3.0 mL of anhydrous DMF and 2.64 mmol of anhydrous K_2_CO_3_) was incubated at room temperature for 15 min. The appropriate substituted halide (0.99 mmol) was then added, and the reaction was heated to 50 °C for 2 h. After cooling, 20 mL of ice-cold water was added, and the precipitate formed was recovered by vacuum filtration and purified by flash column chromatography using dichloromethane/methanol 10:0.5 (for **23a**) or water/acetic acid 1:1 (for **23c**) as the eluent.

#### 3.1.8. 8-Chloro-5-methoxypyrazolo[1,5-*a*]quinazoline-3-carboxamide (**23a**)

Compound **3b** was treated with methyl iodide. Yield 80%, mp 288–290 °C (EtOH); TLC eluent: dichloromethane/methanol 10/0.5 *v*/*v*. ^1^H-NMR (400 MHz, DMSO-d_6_) δ 4.21 (s, 3H, OCH_3_); 7.28 (exch br s, 1H, CONH_2_); 7.51 (exch br s, 1H, CONH_2_); 7.70 (dd, 1H, H_7_, *J*_1_ = 2.0 Hz, *J*_2_ = 8.8 Hz); 8.20 (d, 1H, H_6_, *J* = 8.8 Hz); 8.29 (d, 1H, H_9_, *J* = 2.0 Hz); 8.35 (s, 1H, H_2_). ESI-MS calcd for C_12_H_9_ClN_4_O_2_, 276.68; found: *m*/*z* 278.04 [M + H]^+^. Anal. calcd for C_12_H_9_ClN_4_O_2_ (C, H, N): C, 52.09; H, 3.28; N, 20.25; found: C, 52.30; H, 3.29; N, 20.33.

#### 3.1.9. 8-Chloro-5-(4-sulfamoylbenzyloxy)pyrazolo[1,5-*a*]quinazoline-3-carboxamide (**23c**)

Compound **3b** was treated with 4-(bromomethyl)benzene sulphonamide. Yield 61%, mp 177–180 °C (H_2_O/CH_3_COOH); TLC eluent: toluene/ethyl acetate/methanol 8/2/1.5 *v*/*v*/*v*. ^1^H-NMR (400 MHz, DMSO-d_6_) δ 5.78 (s, 2H, OCH_2_); 7.26 (exch br s, 1H, CONH_2_); 7.39 (exch br s, 2H, SO_2_NH_2_); 7.48 (exch br s, 1H, CONH_2_); 7.69 (dd, 1H, H_7_*, J*_1_ = 1.6 Hz, *J*_2_ = 8.4 Hz); 7.75 (d, 2H, H_2′_, H_6′_, *J* = 8.0 Hz); 7.85 (d, 2H, H_3′_, H_5′_, *J* = 8.0 Hz); 8.27 (d, 1H, H_6_, *J* = 8.8 Hz); 8.29 (d, 1H, H_9_, *J* = 2.0 Hz); 8.35 (s, 1H, H_2_). ^13^C-NMR (100 MHz, DMSO-d_6_) δ 68.93; 106.00; 110.68; 114.76; 126.38; 127.07; 128.51; 128.77; 137.50; 140.16; 140.69; 142.10; 143.20; 146.70; 159.20; 163.09. ESI-MS calcd for C_18_H_14_ClN_5_O_4_S, 431.85; found: *m*/*z* 433.04 [M + H]^+^. Anal. calcd for C_18_H_14_ClN_5_O_4_S (C, H, N): C, 50.06; H, 3.27; N, 16.22; found: C, 50.26; H, 3.28; N, 16.28.

#### 3.1.10. (*E*)-8-Chloro-*N*-[(dimethylamino)methylene]-5-oxo-4,5-dihydropyrazolo[1,5-*a*]quinazoline-3-carboxamide (**26**)

To a solution of 0.21 mmol of **3b** in 3 mL of anhydrous toluene and 0.3 mL of dry DMF, 0.76 mmol (0.10 mL) of DMF-DMA was added. The reaction was refluxed for 2 h. After cooling, the precipitate formed was recovered by vacuum filtration to obtain compound **26**. Yield 83%, mp 288–290 °C (EtOH); TLC eluent: toluene/ethyl acetate/methanol 8/2/2 *v*/*v*/*v*. ^1^H-NMR (400 MHz, DMSO-d_6_) δ 3.15 (s, 3H, NCH_3_); 3.23 (s, 3H, NCH_3_); 7.60 (d, 1H, H_7_, *J* = 8.4 Hz); 8.07 (s, 1H, H_9_); 8.17 (d, 1H, H_6_, *J* = 8.8 Hz); 8.19 (s, 1H, H_2_); 8.67 (s, 1H, N=CH); 10.83 (exch br s, 1H, CONH). ESI-MS calcd for C_14_H_12_ClN_5_O_2_, 317.73; found: *m*/*z* 319.07 [M + H]^+^. Anal. calcd for C_14_H_12_ClN_5_O_2_ (C, H, N): C, 52.92; H, 3.81; N, 22.04; found: C, 52.71; H, 3.79; N, 21.95.

#### 3.1.11. General Procedure for Synthesizing Compounds **33a**, **b**

Compounds **3c** or **3d** (the synthesis of **3c** and **3d** is reported in Supplementary Materials) (0.35 mmol) were treated in 2.5 mL of anhydrous DMF and 0.35 mmol of K_2_CO_3_ with stirring at room temperature for 15 min. Methyl iodide (0.70 mmol) was then added, and the reaction was heated at 80 °C for 1 h. After cooling, 20 mL of ice-cold water was added, and the precipitate formed was recovered by vacuum filtration. The crude compounds were purified by flash column chromatography using dichloromethane/methanol/acetic acid 97/3/03 *v*/*v*/*v* (for **33a**) or toluene/ethyl acetate/acetic acid 8/2/1 *v*/*v*/*v* (for **33b**) as the eluent.

#### 3.1.12. 5-Methoxy-8-nitropyrazolo[1,5-*a*]quinazoline-3-carboxamide (**33a**)

Synthesized from 8-nitro-5-oxo-4,5-dihydropyrazolo[1,5-*a*]quinazoline-3-carboxamide **3c**. Yield 58%, mp >300 °C; TLC eluent: dichloromethane/methanol/acetic acid 97/3/03 *v*/*v*/*v*. ^1^H-NMR (400 MHz, DMSO-d_6_) δ 4.25 (s, 3H, CH_3_); 7.27 (exch br s, 1H, NH_2_); 7.56 (exch br s, 1H, NH_2_); 8.36 (dd, 1H, H_2_, *J*_1_ = 2.0 Hz, *J*_2_ = 8.8 Hz); 8.42 (m, 2H, H_7_, H_6_); 8.88 (d, 1H, H_9_, *J* = 1.6 Hz). ^13^C-NMR (400 MHz, DMSO-d_6_) δ 56.1; 110.5; 120.5; 128.8; 131.2; 144.9. ESI-MS calcd for C_12_H_9_N_5_O_4_, 287.24; found: *m*/*z* 288.07 [M + H]^+^. Anal. calcd for C_12_H_9_N_5_O_4_ (C, H, N): C, 50.18; H, 3.16; N, 24.38; found: C, 50.38; H, 3.17; N, 24.47.

#### 3.1.13. 5-Methoxy-7-nitropyrazolo[1,5-*a*]quinazoline-3-carboxamide (**33b**)

Synthesized from 7-nitro-5-oxo-4,5-dihydropyrazolo[1,5-*a*]quinazoline-3-carboxamide **3d**. Yield 40%, mp 269–271 °C; TLC eluent: toluene/ethyl acetate/acetic acid 8/2/1 *v*/*v*/*v*. ^1^H-NMR (400 MHz, DMSO-d_6_) δ 4.26 (s, 3H, CH_3_); 7.28 (exch br s, 1H, NH_2_); 7.58 (exch br s, 1H, NH_2_); 8.44 (s, 1H, H_2_); 8.47 (d, 1H, H_9_, *J* = 9.2 Hz); 8.77 (dd, 1H, H_8_, *J*_1_ = 2.4 Hz, *J*_2_ = 9.2 Hz); 8.86 (d, 1H, H_6_, *J* = 2.4 Hz). ^13^C-NMR (400 MHz, DMSO-d_6_) δ 56.1; 107.4; 112.1; 117.1; 122.3; 130.1; 140.2; 144.8; 145.7; 160.4; 163.0. ESI-MS calcd for C_12_H_9_N_5_O_4_, 287.24; found: *m*/*z* 288.07 [M + H]^+^. Anal. calcd for C_12_H_9_N_5_O_4_ (C, H, N): C, 50.18; H, 3.16; N, 24.38; found: C, 50.38; H, 3.17; N, 24.47.

#### 3.1.14. 5-(Methylamino)-8-nitropyrazolo[1,5-*a*]quinazoline-3-carbonitrile (**42a**)

To a solution of 0.25 mmol of **39a** (the synthesis of **39a** is reported in Supplementary Materials) in 4.0 mL of 1,4-dioxane, 0.76 mmol of methylamine and 0.38 mmol of *N*,*N*-diisopropylethylamine (DIPEA) were added. The mixture was stirred at room temperature for 1.5 h, 20 mL of ice-cold water was added, and the precipitate obtained was recovered by vacuum filtration to obtain the desired compound. Yield 67%, mp >300 °C (EtOH); TLC eluent: toluene/ethyl acetate/methanol 8/2/2 *v*/*v*/*v*. ^1^H-NMR (400 MHz, DMSO-d_6_) δ 3.06 (d, 3H, CH_3_ *J* = 4.4 Hz); 8.37 (d, 1H, H_7_, *J* = 2.0 Hz); 8.39 (s, 1H, H_2_); 8.54 (d, 1H, H_6_, *J* = 8.8 Hz); 8.75 (d, 1H, H_9_, *J* = 2.0 Hz); 8.98 (exch br d, 1H, NH). ^13^C-NMR (400 MHz, DMSO-d_6_) δ 28.7; 66.4; 114.5; 116.1; 119.9; 121.8; 125.5; 135.7; 146.1; 150.2; 154.4; 163.2. ESI-MS calcd for C_12_H_8_N_6_O_2_, 268.24; found: *m*/*z* 269.07 [M + H]^+^. Anal. calcd for C_12_H_8_N_6_O_2_ (C, H, N): C, 53.73; H, 3.01; N, 31.33; found: C, 53.51; H, 2.99; N, 31.20.

#### 3.1.15. 5-(Methylamino)-8-nitropyrazolo[1,5-*a*]quinazoline-3-carboxamide (**43**)

Compound **42a** (0.82 mmol) was transformed into the corresponding carboxamide following the same procedure used to obtain compound **38**. Yield 70%, mp >300 °C (EtOH); TLC eluent: toluene/ethyl acetate/methanol 8/2/2 *v*/*v*/*v*. ^1^H-NMR (400 MHz, DMSO-d_6_) δ 3.04 (s, 3H, CH_3_); 7.24 (exch br s, 1H, NH); 7.48 (exch br s, 1H, NH); 8.14 (s, 1H, H_2_); 8.30 (d, 1H, H_7_, *J* = 8.4 Hz); 8.52 (d, 1H, H_6_, *J* = 8.8 Hz); 8.74 (s, 2H, H_9_ + NH). ESI-MS calcd for C_12_H_10_N_6_O_3_, 286.25; found: *m*/*z* 287.08 [M + H]^+^. Anal. calcd for C_12_H_10_N_6_O_3_ (C, H, N): C, 50.35; H, 3.52; N, 29.36; found: C, 50.55; H, 3.53; N, 29.47.

### 3.2. Biological Assays

#### 3.2.1. Analysis of AP-1/NF-κB Activation

THP-1 cells are a human monocyte cell line that was developed from a monocyte isolated from the peripheral blood of an acute monocytic leukemia patient. This cell line is used as a monocyte/macrophage model in immunology research. The THP-1Blue cells obtained from InvivoGen (San Diego, CA, USA) are THP-1 cells that were stably transfected with a secreted embryonic alkaline phosphatase gene that is under control of a NF-κB/AP-1-inducible promoter. For this study, THP-1Blue cells were cultured at 37 °C in a humidified atmosphere containing 5% CO_2_ in in RPMI 1640 medium (Mediatech Inc., Herndon, VA, USA) supplemented with 10% (*v*/*v*) fetal bovine serum (FBS), 100 μg/mL streptomycin, 100 U/mL penicillin, 100 μg/mL phleomycin (Zeocin), and 10 μg/mL blasticidin S (all from Sigma-Aldrich, St. Louis, MO, USA).

To measure the activation of AP-1/NF-κB, the THP-1Blue cells (2 × 10^5^ cells/well) were pretreated with the test compounds or dimethyl sulfoxide (DMSO; 1% final concentration) for 30 min, followed by the addition of 250 ng/mL of lipopolysaccharide (LPS; from *Escherichia coli* strain 0111:B4) for 24 h, and alkaline phosphatase activity was measured in the cell supernatants using the QUANTI-Blue mix (InvivoGen) as the absorbance at 655 nm and compared with positive control samples (LPS). The concentration of the compound that caused 50% inhibition of the NF-κB reporter activity (IC_50_) was calculated.

#### 3.2.2. Cytotoxicity Assay

Cytotoxicity was analyzed with a CellTiter-Glo Luminescent Cell Viability Assay Kit from Promega (Madison, WI, USA) according to the manufacturer’s protocol. THP-1Blue cells were treated with varying concentrations of the test compounds (up to 50 µM) and cultivated for 24 h. After treatment, the cells were allowed to equilibrate to room temperature for 30 min, the substrate was added, and the samples were analyzed with a Fluoroscan Ascent FL (Thermo Fisher Scientific, Waltham, MA, USA).

#### 3.2.3. Kinase *K*_d_ Determination

Compounds **13i** and **16** were submitted for dissociation constant (*K*_d_) determination toward JNK1-3 using KINOMEscan (Eurofins Pharma Discovery, San Diego, CA, USA), as described previously [56]. In brief, JNK1-3 were produced and displayed on T7 phages or expressed in HEK-293 cells. Binding reactions were performed at room temperature for 1 h, and the fraction of kinase not bound to the test compound was determined by capture with an immobilized affinity ligand and quantified by quantitative polymerase chain reaction. The primary screening at fixed concentrations of the compound was performed in duplicate. For dissociation constant *K*_d_ determination, a 12-point half-log dilution series (a maximum concentration of 33 μM) was used. Assays were performed in duplicate, and their average mean value is displayed.

### 3.3. Molecular Modeling

#### 3.3.1. PharmMapper Modeling

The PharmMapper Server [58] was used to identify potential protein targets for compounds **13i** and **16**. PharmMapper recognizes potential targets based on reverse pharmacophore mapping. The protein biotargets are represented by sets of pharmacophore points in reference databases incorporated in the software. The structures of **13i** and **16** were uploaded in SDF format into PharmMapper. The system automatically generated up to 300 conformers of each compound based on the software option. We performed pharmacophore mapping using the “Human Protein Targets Only” database, which contained 2241 targets. We retrieved the top 250 potential targets for each compound evaluated. The potential targets were sorted by normalized fit score.

#### 3.3.2. Molecular Docking

Docking of compounds **13i** and **16** into the binding sites of kinases ERK2, JNK3, and p38α MAPK (structures 1TVO [54], 1PMV [59], and 1W7H [60], respectively, from Protein Data Bank) was performed with the use of the ROSIE server [52]. The docking areas were chosen around the geometric centers of the co-crystallized ligands, each occupying the binding site of the corresponding enzyme in the 1TVO, 1PMV, or 1W7H structure. For each of the docked compounds, generation of up to 1000 ligand conformers with the BCL algorithm [61] was switched on. The number of intermediately generated docking poses was set to 2000. Other options were set to the default settings within the ROSIE ligand docking protocol, which accounts for the full flexibility of the main chain and side chains of residues in the vicinity of the docking area [50]. Upon finishing the computation jobs, PDB files containing the best poses obtained for compounds **13i** and **16** docked into ERK2, JNK3, and p38α were downloaded from the server, and imported into the Molegro Virtual Docker (MVD) program for visualization and analysis using the built-in “Pose Organizer” tool of MVD.

The docking of compounds **13i** and **16** into the JNK3 binding site (PDB structure 4WHZ) was performed with AUTODOCK 4.1. For each investigated complex, the conformation with the most favorable binding energy was selected. The JNK3–ligand complex was further minimized in a vacuum using the amber99sb force-field implemented in GROMACS 5.1. No constraints were applied, and a conjugate gradient algorithm for energy minimization was used. The minimization was converged when the maximum force was smaller than 10.0 kJ·mol^−1^·nm^−1^.

## 4. Conclusions

In this manuscript, we report the biological screening of 80 pyrazolo[1,5-*a*]quinazoline compounds and related derivatives (most being new and unpublished compounds) to investigate their potential anti-inflammatory effects. All compounds were screened for their ability to inhibit NF-κB/AP-1 reporter activity in THP-1Blue cells since this pathway is fundamental in inflammatory processes. Of the screened compounds, 13 were able to inhibit NF-κB/AP-1 activity with IC_50_ values <50 μM. Considering that this was a library of non-homogeneous compounds, only a few observations could be made to highlight several structural features that correlated with anti-inflammatory activity. The PharmMapper analysis indicated that the most potent compounds may be MAPK kinase ligands. This conclusion was supported by molecular modeling studies showing that the selected compounds **13i** and **16** could effectively bind to ERK2, p38α, and JNK3 and the KINOMEscan studies showing that these compounds can bind to JNK1, JNK2, and JNK3 with *K*_d_ values in the micromolar range. Thus, pyrazolo[1,5-*a*]quinazoline and related scaffolds may be novel structures to explore for the development of new anti-inflammatory therapeutics targeted toward MAPKs. Future studies will be important to evaluate these lead compounds in in vivo models of inflammation.

## Figures and Tables

**Table 1:**
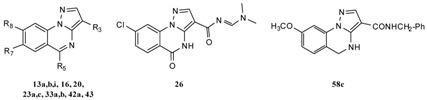
Summary of compound cytotoxic activity and inhibitory effects on LPS-induced NF-κB/AP-1 transcriptional activity in THP-1Blue cells.

	R_3_	R_5_	R_7_	R_8_	AP Production IC_50_ (µM)	Cytotoxicity
**13a**	CONH_2_	OCH_3_	H	H	24.4 ± 3.1	N.T.
**13b**	CONH_2_	OCH_2_Ph	H	H	4.8 ± 1.2	N.T.
**13i**	CONH_2_	OCH_2_(4-SO_2_NH_2_)-Ph	H	H	9.7 ± 2.4	N.T.
**16**	CONH_2_	OCH_2_(4-SOCH_3_)Ph	H	H	7.9 ± 1.7	N.T.
**20**	3-tienyl	OSO_2_(4-Me)Ph	H	H	13.3 ± 2.6	N.T.
**23a**	CONH_2_	OCH_3_	H	Cl	7.9 ± 1.6	N.T.
**23c**	CONH_2_	OCH_2_(4-SO_2_NH_2_)-Ph	H	Cl	12.2 ± 2.1	N.T.
**33a**	CONH_2_	OCH_3_	H	NO_2_	14.1 ± 2.9	N.T.
**33b**	CONH_2_	OCH_3_	NO_2_	H	18.2 ± 2.5	N.T.
**42a**	CN	NHCH_3_	H	NO_2_	30.1 ± 3.3	N.T.
**43**	CONH_2_	NHCH_3_	H	NO_2_	11.8 ± 2.2	N.T.
**26**					49.3 ± 4.6	N.T.
**58c** [42]					34.1 ± 4.1	N.T.

N.T., no cytotoxicity was found in THP-1Blue cells at concentrations up to 50 µM. AP, alkaline phosphatase.

**Table 2:** Potential human protein kinase targets for compounds **13i** and **16** identified by PharmMapper.

**PDB**	**Kinase Target for Compound 13i**	**Fit Score**	**Normalized Fit Score**
1PME	Mitogen-activated protein kinase 1 (ERK2)	2.976	0.9919
1MUO	Serine/threonine protein kinase 6 (PAK6)	2.949	0.983
1W7H	Mitogen-activated protein kinase 14 (p38α)	2.902	0.9672
3HVC	Mitogen-activated protein kinase 14 (p38α)	2.876	0.9588
1PMV	Mitogen-activated protein kinase 10 (JNK3)	2.81	0.9368
1UKI	Mitogen-activated protein kinase 8 (JNK1)	2.807	0.9358
2P3G	MAP kinase-activated protein kinase 2	2.799	0.9329
**PDB**	**Kinase Target for Compound 16**	**Fit Score**	**Normalized Fit Score**
1PME	Mitogen-activated protein kinase 1 (ERK2)	2.991	0.9972
1W7H	Mitogen-activated protein kinase 14 (p38α)	2.969	0.9896
3HVC	Mitogen-activated protein kinase 14 (p38α)	2.893	0.9645
2VTA	Cell division protein kinase 2	2.873	0.9577
2BRG	Serine/threonine protein kinase Chk1	2.862	0.9539
1PMV	Mitogen-activated protein kinase 10 (JNK3)	2.811	0.9372
1UKI	Mitogen-activated protein kinase 8 (JNK1)	2.809	0.9364
2A4Z	Phosphatidylinositol-4,5-bisphosphate 3-kinase catalytic subunit γ-isoform	2.796	0.9321
2C3I	Serine/threonine protein kinase Pim-1	2.725	0.9082

**Table 3:** Interface energy scores and H-bonding interactions obtained from the docking of ligands **13i** and **16** into the binding sites of ERK2, JNK3, and p38α obtained using the ROSIE web server.

Ligand	Interface Energy Scores and H-Bonded Groups in the Protein and Ligand		
	ERK2 (1TVO)	JNK3 (1PMV)	p38α (1W7H)
**13i**	−16.49	−21.00	−16.53
	Lys54, Arg67 (pyrazole nitrogen); **Glu71** (amide NH_2_); Gln105 (amide oxygen); Met108 (sulfonamide NH_2_); **Lys114** (sulfonamide oxygen)	**Gln75**, Glu111 (amide NH_2_); **Lys93** (amide oxygen, pyrimidine nitrogen); Ser72 (sulfonamide oxygen); Asn152 (sulfonamide oxygen)	Gly110, Asp112 (amide NH_2_); **Lys53** (sulfonamide oxygen); Glu71 (sulfonamide NH_2_)
**16**	−17.45	−19.36	−17.29
	**Glu71**, Asp167 (amide NH_2_); **Lys114** (sulfoxide oxygen)	**Gln75**, Asn194 (amide NH_2_); **Lys93** (pyrazole nitrogen); Gln155 (sulfoxide oxygen)	**Lys53** (pyrazole nitrogen); Asp168 (amide NH_2_)

Common amino acid residues participating in the interactions with both ligands **13i** and **16** are shown in bold.

**Table 4:** JNK binding affinity of the compounds **13i** and **16**.

Compound	JNK1	JNK2	JNK3
	*K*_d_ (µM)		
**13i**	10.9 ± 1.4	18.5 ± 2.3	9.0 ± 1.2
**16**	17.0 ± 2.1	21.0 ± 2.4	10.4 ± 1.5

## Data Availability

The data are contained within the article and Supplementary Materials.

## Supplementary Materials

The following supporting information can be downloaded at: https://www.mdpi.com/article/10.3390/molecules29112421/s1, Tables S1–S3: Synthetic procedures for all new compounds; Chemical structures of all tested compounds; Figures S1–S59: ^1^H-NMR and ^13^C-NMR spectra of representative compounds; Figures S60–S63: HSQC and HMBC of compounds **7b** and **14c**; Table S4: Potential human protein targets for compounds **13i** and **16** identified by PharmMapper; Figures S64 and S65: Molecular modeling for complexes of compounds **13i** and **16** with JNK3 (**PDB:** 4WHZ); Table S5: H-bonding interactions obtained from the docking of ligands **13i** and **16** into the binding sites of JNK3.
