# Supplementary material for: Anti-Inflammatory Activity of Pyrazolo[1,5-a]quinazolines
Source: Molecules. 2024 May 21;29(11):2421. doi: 10.3390/molecules29112421 (PMC11173647; doi:10.3390/molecules29112421)
Supplement: Supplementary file 1 [file molecules-29-02421-s001.zip › molecules-2981589-supplementary.pdf]

Supplementary Materials

for

Anti-Inflammatory Activity of Pyrazolo[1,5-*a*]quinazolines

Letizia Crocetti[a], Andrei I. Khlebnikov[b], Gabriella Guerrini[a*], Igor A. Schepetkin[c], Fabrizio Melani[a], Maria Paola Giovannoni[a] and Mark T. Quinn[c*]

[a]*NEUROFARBA, Pharmaceutical and Nutraceutical Section, University of Florence, Via Ugo Schiff 6, 50019 Sesto Fiorentino, Italy*

[b]*National Research Tomsk Polytechnic University, Tomsk 634050, Russia*

[c]*Department of Microbiology and Cell Biology, Montana State University, Bozeman, MT 59717, USA*

[*]**Corresponding Authors:** Gabriella Guerrini: email: gabriella.guerrini@unifi.it
Mark T. Quinn, Ph.D.: email: mquinn@montana.edu

Table of contents (references are as listed in the main manuscript).

1. **Synthetic procedures for all new compounds**
2. **Chemical structures of all tested compounds (Tables S1-S3)**
3. **^{1}H-NMR and ^{13}C-NMR of representative compounds (Figures S1-S59)**
4. **HSQC and HMBC of compounds 7b and 14c (Figures S60-S63)**
5. **Potential human protein targets for compounds 13i and 16 identified by PharmMapper (Table S4)**
6. **Molecular modelling for complexes of compounds 13i and 16 with JNK3 (PDB: 4WHZ) (Figures S64-S65)**
7. **H-bonding interactions obtained from the docking of ligands 13i and 16 into the binding sites of JNK3 (Table S5)**

1. Synthetic procedures for all new compounds

General procedure for compounds 2b-d. To a solution of the appropriate R_4/R_5-hydrazino benzoic acid **1a-d** (0.80 mmol) (**1a** [29], **1b** [30], **1c** [31], **1d** [32]) in 2.5 mL of dry DMF, 0.80 mmol of (ethoxymethylene)malononitrile and 0.96 mmol of anhydrous sodium acetate were added. The mixture was stirred at reflux temperature for 2 hours. After cooling, ice-cold water was added (20 mL) and the precipitate formed was recovered by vacuum filtration to obtain the desired compounds which were purified by crystallisation from isopropyl alcol (for **2b**) or ethanol (**for 2c,d**).

8-Chloro-5-oxo-4,5-dihydropyrazolo[1,5-*a*]quinazoline-3-carbonitrile (2b). Yield 91%, mp >300 °C (*i*-PrOH); TLC eluent: toluene/ethyl acetate/methanol 8/2/1.5 v/v/v. ^{1}H-NMR (400 MHz, DMSO-d_6) δ 7.62 (d, 1H, H_7, J = 8.4 Hz); 8.07 (s, 1H, H_9); 8.14 (d, 1H, H_6, J = 8.4 Hz); 8.37 (s, 1H, H_2); 13.38 (exch br s, 1H, CONH). ESI-MS calcd. for $C_{11}H_5ClN_4O$, 244.64; found: m/z 246.01 $[M + H]^+$. Anal. calcd for $C_{11}H_5ClN_4O$ (C, H, N): C, 54.01; H, 2.06; N, 22.90; found: C, 54.22; H, 2.07; N, 22.99.

8-Nitro-5-oxo-4,5-dihydropyrazolo[1,5-*a*]quinazoline-3-carbonitrile (2c). Yield 70%, mp 280-283 °C (EtOH); TLC eluent: toluene/ethyl acetate/methanol 8/2/2 v/v/v. ^{1}H-NMR (400 MHz, DMSO-d_6) δ 8.29 (dd, 1H, H_9, J_1 = 2,0 Hz, J_2 = 8,8 Hz); 8.37 (d, 1H, H_6, J = 8,8 Hz); 8.43 (s, 1H, H_2); 8.64 (d, 1H, H_9, J = 2,0 Hz); 13.61 (exch br s, 1H, CONH). ESI-MS calcd. for $C_{11}H_5N_5O_3$, 255.19; found: m/z 256.04 $[M + H]^+$. Anal. calcd for $C_{11}H_5N_5O_3$ (C, H, N): C, 51.77; H, 1.97; N, 27.44; found: C, 51.56; H, 1.96; N, 27.33.

7-Nitro-5-oxo-4,5-dihydropyrazolo[1,5-*a*]quinazoline-3-carbonitrile (2d). Yield 71%, mp >295 °C (EtOH); TLC eluent: toluene/ethyl acetate/methanol 8/2/2 v/v/v. ^{1}H-NMR (400 MHz, DMSO-d_6) δ 8.27 (d, 1H, H_9, J= 9,2 Hz); 8.45 (s, 1H, H_2); 8.68 (dd, 1H, H_8, J_1 = 2,4 Hz, J_2 = 9,2 Hz); 8.78 (d, 1H, H_6, J = 2,0 Hz); 13.66 (exch br s, 1H, NH). IR (KBr) ν (cm^{-1}): 3120 (NH); 2240 (CN); 1700 (CO); 1520-1350 (NO_2). ESI-MS calcd. for $C_{11}H_5N_5O_3$, 255.19; found: m/z 256.04 $[M + H]^+$. Anal. calcd for $C_{11}H_5N_5O_3$ (C, H, N): C, 51.77; H, 1.97; N, 27.44; found: C, 51.56; H, 1.96; N, 27.33.

General procedure for compounds 3b-d. A solution of intermediates **2b-d** (0.82 mmol) in 2 mL of H_2SO_4 conc. was stirred at 80 °C for 2 hours. After cooling, the mixture was slowly poured into water and ice. The precipitate formed was isolated by vacuum filtration to obtain the desired product.

8-Chloro-5-oxo-4,5-dihydropyrazolo[1,5-*a*]quinazoline-3-carboxamide (3b). Yield 88%, mp > 300 °C (2-methoxyethanol); TLC eluent: toluene/ethyl acetate/methanol 8/2/1.5 v/v/v. ^{1}H-NMR (400 MHz, DMSO-d$_6$) δ 7.35 (exch br s, 1H, CONH$_2$); 7.59 (dd, 1H, H$_7$, J_1 = 2.0 Hz, J_2 = 8.4 Hz); 7.87 (exch br s, 1H, CONH$_2$); 8.05 (d, 1H, H$_9$, J = 2.0 Hz); 8.15 (d, 1H, H$_6$, J = 8.4 Hz); 8.30 (s, 1H, H$_2$); 10.72 (exch br s, 1H, CONH). ESI-MS calcd. for $C_{11}H_7ClN_4O_2$, 262.65; found: m/z 264.02 $[M + H]^+$. Anal. calcd for $C_{11}H_7ClN_4O_2$ (C, H, N): C, 50.30; H, 2.69; N, 21.33; found: C, 50.50; H, 2.70; N, 21.41.

8-Nitro-5-oxo-4,5-dihydropyrazolo[1,5-*a*]quinazoline-3-carboxamide (3c). Yield 74%, mp > 295 °C (EtOH); TLC eluent: dichloromethane/methanol 9/1 v/v. ^{1}H-NMR (400 MHz, DMSO-d$_6$) δ 7.39 (exch br s, 1H, CONH$_2$); 7.88 (exch br s, 1H, CONH$_2$); 8.26 (dd, 1H, H$_7$, J_1 = 2,0 Hz, J_2 = 8,8 Hz); 8.38 (d, 1H, H$_6$, J = 8,4 Hz); 8.34 (s, 1H, H$_2$); 8.65 (d, 1H, H$_9$, J = 2,4 Hz); 11.00 (exch br s, 1H, NH). ESI-MS calcd. for $C_{11}H_7N_5O_4$, 273.21; found: m/z 274.05 $[M + H]^+$. Anal. calcd for $C_{11}H_7N_5O_4$ (C, H, N): C, 48.36; H, 2.58; N, 25.63; found: C, 48.55; H, 2.59; N, 25.73.

7-Nitro-5-oxo-4,5-dihydropyrazolo[1,5-*a*]quinazoline-3-carboxamide (3d). Yield 75%, mp >295 °C (EtOH); TLC eluent: toluene/ethyl acetate/acetic acid 8/2/1 v/v/v. ^{1}H-NMR (400 MHz, DMSO-d$_6$) 7.40 (exch br s, 1H, CONH$_2$); 7.88 (exch br s, 1H, CONH$_2$); 8.24 (d, 1H, H$_9$, J = 9,2 Hz); 8.36 (s, 1H, H$_2$); 8.67 (dd, 1H, H$_8$, J_1 = 2,8 Hz, J_2 = 9,2 Hz); 8.79 (d, 1H, H$_6$, J = 2,4 Hz); 11.06 (exch br s, 1H, NH). ESI-MS calcd. for $C_{11}H_7N_5O_4$, 273.21; found: m/z 274.05 $[M + H]^+$. Anal. calcd for $C_{11}H_7N_5O_4$ (C, H, N): C, 48.36; H, 2.58; N, 25.63; found: C, 48.55; H, 2.59; N, 25.73.

General procedure for compounds 4c,d. To a solution of 2-hydrazineyl-4-nitrobenzoic acid **1c** [31] or 2-hydrazineyl-5-nitrobenzoic acid **1d** [32] (0.42 mmol) in 3 mL of dry DMF, 0.43 mmol of ethyl 2-cyano-3-ethoxyacrylate and 0.42 mmol of anhydrous sodium acetate were added. The mixture was stirred at reflux temperature for 2 hours. After cooling, ice-cold water was added (20 mL) and the

precipitate formed was recovered by vacuum filtration to obtain the desired compounds which were purified by crystallisation.

Ethyl 8-nitro-5-oxo-4,5-dihydropyrazolo[1,5-*a*]quinazoline-3-carboxylate (4c). Yield 59%, mp 195-197 °C (EtOH); TLC eluent: toluene/ethyl acetate/methanol 8/2/2 v/v/v. ^{1}H-NMR (400 MHz, DMSO-d$_6$) δ 1.27 (t, 3H, CH_3, J = 7,0 Hz); 4.29 (q, 2H, CH_2, J = 7,0 Hz); 8.26 (m, 2H, H_7, H_2); 8.38 (d, 1H, H_6, J = 8,8 Hz); 8.66 (s, 1H, H_9); 12.00 (exch br s, 1H, NH). ESI-MS calcd. for $C_{13}H_{10}N_4O_5$, 302.25; found: m/z 303.07 [M + H]$^+$. Anal. calcd for $C_{13}H_{10}N_4O_5$ (C, H, N): C, 51.66; H, 3.34; N, 18.54; found: C, 51.45; H, 3.32; N, 18.46.

Ethyl 7-nitro-5-oxo-4,5-dihydropyrazolo[1,5-*a*]quinazoline-3-carboxylate (4d). Yield 78%, mp 217-220 °C (EtOH); TLC eluent: toluene/ethyl acetate/methanol 8/2/2 v/v/v. ^{1}H-NMR (400 MHz, DMSO-d$_6$) δ 1.29 (t, 3H, CH_3, J = 7,0 Hz); 4.30 (q, 2H, CH_2, J = 7,0 Hz); 8.27 (m, 2H, H_9, H_2); 8.68 (dd, 1H, H_8, J_1 = 2,8 Hz, J_2 = 9,2 Hz); 8.81 (d, 1H, H_6, J = 2,4 Hz); 12.07 (exch br s, 1H, NH). ESI-MS calcd. for $C_{13}H_{10}N_4O_5$, 302.25; found: m/z 303.07 [M + H]$^+$. Anal. calcd for $C_{13}H_{10}N_4O_5$ (C, H, N): C, 51.66; H, 3.34; N, 18.54; found: C, 51.45; H, 3.33; N, 18.46.

8-Nitropyrazolo[1,5-*a*]quinazolin-5(4*H*)-one (5c). A solution of compound **4c** (0.24 mmol) in 5 mL of HCl 37% was stirred at reflux for 4 hours. After cooling, the mixture was extracted with ethyl acetate (3 x 15 mL). The organic layer was evaporated in vacuum, and the residue was purified by crystallisation. Yield 70%, mp >300 °C (EtOH); TLC eluent: toluene/ethyl acetate/ methanol 8/2/2 v/v/v. ^{1}H-NMR (400 MHz, DMSO-d$_6$) δ 5.95 (s, 1H, H_3); 7.85 (s, 1H, H_2); 8.20 (m, 1H, H_6); 8.33 (d,1H, H_7, J = 7.6 Hz); 8.63 (s,1H, H_9); 12.48 (exch br s, 1H, NH). ESI-MS calcd. for $C_{10}H_6N_4O_3$, 230.18; found: m/z 231.05 [M + H]$^+$. Anal. calcd for $C_{10}H_6N_4O_3$ (C, H, N): C, 52.18; H, 2.63; N, 24.34; found: C, 52.39; H, 2.64; N, 24.43.

General procedure for compounds 7b-f. A solution of **4a** [34], **5a** [34] or **2a** [52] (0.23 mmol) in 2.5 mL of dry CH_3CN (or dry DMF for **7e,f**) and anhydrous K_2CO_3 (0.92 mmol) was maintained at room temperature for 15 min., and then the appropriate substituted benzyl bromide (0.35 mmol) was added. The reaction was refluxed, and when the starting material disappeared in TLC (2-24 hours),

the addition of water yielded a precipitate that was filtered, washed with water and diethyl ether and finally purified by crystallisation. (Compound **7a** [30])

Ethyl 4-(4-(methylthio)benzyl)-5-oxo-4,5-dihydropyrazolo[1,5-*a*]quinazoline-3-carboxylate (7b). Starting from **4a** and 4-(methylthio)benzyl bromide.Yield 70%, mp 156-159 °C (EtOH); TLC: toluene/ethyl acetate/acetic acid 8/2/1 v/v/v. ^{1}H-NMR (400 MHz, DMSO-d$_6$) δ 1.20 (t, 3H, CH$_2$*CH$_3$*, *J* = 6.8 Hz); 2.39 (s, 3H, SCH$_3$); 4.17 (q, 2H, *CH$_2$*CH$_3$, *J* = 6.8 Hz); 5.87 (s, 2H, NCH$_2$); 7.09 (d, 2H, H$_{3'}$, H$_{5'}$, *J* = 7.6 Hz); 7.14 (d, 2H, H$_{2'}$, H$_{6'}$, *J* = 8.0 Hz); 7.60 (t, 1H, H$_7$, *J* = 7.6 Hz); 7.96 (t, 1H, H$_8$, *J* = 7.6 Hz); 8.20 (m, 2H, H$_6$ and H$_9$); 8.25 (s, 1H, H$_2$). ^{13}C-NMR (100 MHz, DMSO-d$_6$) δ 14.5; 15.2; 47.7; 61.0; 99.3; 115.7; 115.9; 126.4; 127.1; 127.7; 128.8; 133.9; 136.1; 137.1; 140.4; 145.3; 159.2; 161.9. ESI-MS calcd. for $C_{21}H_{19}N_3O_3S$, 393.46; found: m/z 394.11 [M + H]$^+$. Anal. calcd for $C_{21}H_{19}N_3O_3S$ (C, H, N): C, 64.11; H, 4.87; N, 10.68; found: C, 64.37; H, 4.89; N, 10.72.

Ethyl 5-oxo-4-(4-sulfamoylbenzyl)-4,5-dihydropyrazolo[1,5-*a*]quinazoline-3-carboxylate (7c). Starting from **4a** and 4-(bromomethyl)benzene sulphonamide. Yield 71%, mp 224-226 °C (EtOH); TLC eluent: dichloromethane/methanol 10/0.5 v/v. ^{1}H-NMR (400 MHz, DMSO-d$_6$) δ 1.16 (t, 3H, CH$_2$*CH$_3$*, *J* = 7.2 Hz); 4.11 (q, 2H, *CH$_2$*CH$_3$, *J* = 7.2 Hz); 5.92 (s, 2H, NCH$_2$); 7.29 (exch br s, 2H, SONH$_2$); 7.38 (d, 2H, H$_{2'}$, H$_{6'}$, *J* = 8.0 Hz); 7.60 (t, 1H, H$_7$, *J* = 8.0 Hz); 7.69 (d, 2H, H$_{3'}$, H$_{5'}$, *J* = 8.0 Hz); 7.98 (t, 1H, H$_8$, *J* = 8.0 Hz); 8.20 (m, 2H, H$_6$, H$_9$); 8.27 (s, 1H, H$_2$). ^{13}C-NMR (100 MHz, DMSO-d$_6$) δ 14.5; 48.8; 60.9; 99.2; 115.7; 116.0; 126.1; 127.1; 128.8; 136.1; 137.4; 140.7; 141.7; 143.0; 145.3; 159.2; 161.9. ESI-MS calcd. for $C_{20}H_{18}N_4O_5S$, 426.45; found: m/z 427.10 [M + H]$^+$. Anal. calcd for $C_{20}H_{18}N_4O_5S$ (C, H, N): C, 56.33; H, 4.25; N, 13.14; found: C, 56.55; H, 4.27; N, 13.19.

Ethyl 4-[4-(methylsulfonyl)benzyl]-5-oxo-4,5-dihydropyrazolo[1,5-*a*]quinazoline-3-carbo xylate (7d). Starting from **4a** and 4-(methylsulphonyl)benzyl bromide. Yield 80%, mp 229-232 °C (EtOH 80%); TLC eluent: toluene/ethyl acetate/acetic acid 8/2/1 v/v/v. ^{1}H-NMR (400 MHz, DMSO-d$_6$) δ 1.14 (t, 3H, CH$_2$*CH$_3$*, *J* = 7.2 Hz); 3.16 (s, 3H, SO$_2$CH$_3$); 4.10 (q, 2H, *CH$_2$*CH$_3$, *J* = 7.2 Hz); 5.95 (s, 2H, NCH$_2$); 7.49 (d, 2H, H$_{3'}$, H$_{5'}$, *J* = 8.0 Hz); 7,60 (t, 1H, H$_7$, *J* = 7.6 Hz); 7.81 (d, 2H, H$_{2'}$, H$_{6'}$, *J* = 8.4 Hz); 7.98 (t, 1H, H$_8$, *J* = 7.6 Hz); 8.18 (d, 1H, H$_9$, *J* = 8.0 Hz); 8.22 (d, 1H, H$_6$, *J* = 8.4

Hz); 8.28 (s, 1H, H_2). ^{13}C-NMR (100 MHz, DMSO-d_6) δ 14.4; 43.9; 49.0; 60.9; 99.2; 115.7; 116.0; 127.1; 127.5; 128.7; 129.6; 136.1; 137.4; 139.7; 140.7; 143.8; 145.3; 159.2; 161.9. ESI-MS calcd. for $C_{21}H_{19}N_3O_5S$, 425.46; found: m/z 426.11 $[M + H]^+$. Anal. calcd for $C_{21}H_{19}N_3O_5S$ (C, H, N): C, 59.28; H, 4.50; N, 9.88; found: C, 59.52; H, 4.52; N, 9.92.

4-[4-(Methylthio)benzyl]-5-oxo-4,5-dihydropyrazolo[1,5-*a*]quinazoline-3-carbonitrile (7e). Starting from **2a** and 4-(methylthio)benzyl bromide. Yield 70%, mp 189-192 °C (EtOH/H_2O 9:1); TLC eluent: toluene/ethyl acetate/acetic acid 8/2/1 v/v/v. ^{1}H-NMR (400 MHz, DMSO-d_6) δ (s, 3H, SCH_3); 5.44 (s, 2H, NCH_2); 7.20 (d, 2H, $H_{3'}$, $H_{5'}$, J = 8.4 Hz); 7.27 (d, 2H, $H_{2'}$, $H_{6'}$, J = 8.4 Hz); 7.64 (t, 1H, H_7, J = 7.6 Hz); 7.99 (t, 1H, H_8, J = 7.6 Hz); 8.18 (d, 1H, H_9, J = 8.4 Hz); 8.24 (d, 1H, H_6, J = 8.0 Hz); 8.44 (s, 1H, H_2). ^{13}C-NMR (100 MHz, DMSO-d_6) δ 15.1; 47.2; 113.9; 115.6; 116.4; 126.5; 127.5; 127.6; 129.0; 132.2; 136.3; 136.9; 137.8; 142.9; 145.4; 158.5. ESI-MS calcd. for $C_{19}H_{14}N_4OS$, 346.41; found: m/z 347.09 $[M + H]^+$. Anal. calcd for $C_{19}H_{14}N_4OS$ (C, H, N): C, 65.88; H, 4.07; N, 16.17; found: C, 65.62; H, 4.05; N, 16.10.

4-[4-(Methylthio)benzyl]pyrazolo[1,5-*a*]quinazolin-5(4*H*)-one (7f). Starting from **5a** and 4-(methylthio)benzyl bromide. Yield 90%, mp 137-139 °C (EtOH/H_2O 9:1); TLC eluent: toluene/ethyl acetate/acetic acid 8/2/1 v/v/v. ^{1}H-NMR (400 MHz, DMSO-d_6) δ 2.42 (s, 3H, SCH_3); 5.23 (s, 2H, NCH_2); 6.26 (s, 1H, H_3); 7.19 (d, 2H, $H_{3'}$, $H_{5'}$, J = 8.0 Hz); 7.34 (d, 2H, $H_{2'}$, $H_{6'}$, J = 8.0 Hz); 7.52 (t, 1H, H_7, J = 7.6 Hz); 7.83 (s, 1H, H_2); 7.90 (t, 1H, H_8, J = 7.6 Hz); 8.10 (d, 1H, H_9, J = 8.4 Hz); 8.22 (d, 1H, H_6, J = 8.0 Hz). ^{13}C-NMR (100 MHz, DMSO-d_6) δ 15.1; 47.3; 91.0; 114.8; 115.9; 126.1; 126.5; 128.7; 129.1; 132.9; 135.7; 137.4; 137.9; 140.4; 142.7. ESI-MS calcd. for $C_{18}H_{15}N_3OS$, 321.40; found: m/z 322.10 $[M + H]^+$. Anal. calcd for $C_{18}H_{15}N_3OS$ (C, H, N): C, 67.27; H, 4.70; N, 13.07; found: C, 67.54; H, 4.72; N, 13.12.

4-[4-(Methylsulfonyl)benzyl]pyrazolo[1,5-*a*]quinazolin-5(4H)-one (8). Compound **7f** (0.16 mmol) was dissolved in 4 mL of MeOH. To this solution, at 0 °C, a previously prepared solution composed of 0.64 mmol of OXONE® in 4 mL of H_2O was added. The mixture was stirred at 100 °C for 2 hours. After cooling, 10 mL of H_2O was added and the precipitate formed was recovered by

vacuum filtration to obtain the desired compound. Yield 88%, mp 186-188 °C (EtOH); TLC eluent: toluene/ethyl acetate/acetic acid 8/2/1 v/v/v. ^{1}H-NMR (400 MHz, DMSO-d$_6$) δ 3.16 (s, 3H, SO_2CH_3); 5.39 (s, 2H, NCH_2); 6.25 (s, 1H, H_3); 7.53 (t, 1H, H_7, J = 7.2 Hz); 7.63 (d, 2H, $H_{2'}$, $H_{6'}$, J = 7.6 Hz); 7.85 (m, 3H, $H_{3'}$, $H_{5'}$, H_2); 7.92 (t, 1H, H8, J = 7.2 Hz); 8.12 (d, 1H, H_9, J = 8.0 Hz); 8.22 (d, 1H, H_6, J = 7.6 Hz). ^{13}C-NMR (100 MHz, DMSO-d$_6$) δ 43.9; 47.5; 90.9; 114.8; 115.8; 126.1; 127.7; 128.5; 129.1; 135.8; 137.5; 140.4; 140.5; 142.3; 142.7; 158.5. ESI-MS calcd. for $C_{18}H_{15}N_3O_3S$, 353.40; found: m/z 354.09 $[M + H]^+$. Anal. calcd for $C_{18}H_{15}N_3O_3S$ (C, H, N): C, 61.18; H, 4.28; N, 11.89; found: C, 61.42; H, 4.30; N, 11.94.

General procedure for compounds 9 and 10. To a solution of 176 mg (1 mmol) of HIO_3 in 11 mL of acetone/H_2O (10:1), a small amount of tetra-n-butylammonium bromide (TBAB) was added while stirring for 5 minutes. Then, 0.25 mmol of compound **7b** or **7f** were added, and the mixture was stirred at 80 °C for 2 hours. The reaction mixture was cooled, 10 mL of H_2O was added, and the precipitate was collected by vacuum filtration.

3-Iodo-4-[4-(methylsulfinyl)benzyl]pyrazolo[1,5-*a*]quinazolin-5(4*H*)-one (9). Yield 52%, mp 198-201 °C (EtOH); TLC eluent: toluene/ethyl acetate/acetic acid 8/2/1 v/v/v. ^{1}H-NMR (400 MHz, DMSO-d$_6$) δ 2.71 (s, 3H, $SOCH_3$); 5.71 (s, 2H, NCH_2); 7.45 (d, 2H, $H_{2'}$, $H_{6'}$, J = 8.4 Hz); 7.53 (t, 1H, H_7, J = 7.6 Hz); 7.63 (d, 2H, $H_{3'}$, $H_{5'}$, J = 8.0 Hz); 7.90 (s, 1H, H_2); 7.92 (t, 1H, H_8, J = 7.2 Hz); 8.14 (d, 1H, H_9, J = 4.0 Hz); 8.16 (d, 1H, H_6, J = 4.0 Hz). ^{13}C-NMR (100 MHz, DMSO-d$_6$) δ 43.6; 46.8; 114.7; 115.7; 124.3; 126.4; 127.4; 128.9; 135.8; 137.3; 138.4; 140.2; 145.5; 149.2; 158.7. ESI-MS calcd. for $C_{18}H_{14}IN_3O_2S$, 463.29; found: m/z 463.99 $[M + H]^+$. Anal. calcd for $C_{18}H_{14}IN_3O_2S$ (C, H, N): C, 46.67; H, 3.05; N, 9.07; found: C, 46.48; H, 3.04; N, 9.03.

Ethyl 4-[4-(methylsulfinyl)benzyl]-5-oxo-4,5-dihydropyrazolo[1,5-*a*]quinazoline-3-carboxylate (10). Yield 75%, mp 179-181 °C (EtOH); TLC eluent: toluene/ethyl acetate/acetic acid 8/2/1 v/v/v. ^{1}H-NMR (400 MHz, DMSO-d$_6$) δ 1.14 (t, 3H, $CH_2\mathit{CH_3}$, J = 7.2 Hz); 2.68 (s, 3H, $SOCH_3$); 4.12 (q, 2H, $\mathit{CH_2}CH_3$, J = 7.2 Hz); 5.94 (s, 2H, NCH_2); 7.39 (d, 2H, $H_{2'}$, $H_{6'}$, J = 7.6 Hz); 7.57 (d, 2H, $H_{3'}$, $H_{5'}$, J = 7.6 Hz); 7.60 (t, 1H, H_7, J = 7.2 Hz); 7.97 (t, 1H, H_8, J = 7.2 Hz); 8.20 (m, 2H, H_6, H_9); 8.27

(s, 1H, H_2). ^{13}C-NMR (100 MHz, DMSO-d_6) δ 14.4; 43.6; 48.6; 60.9; 99.3; 115.7; 116.0; 124.1; 127.1; 127.6; 128.8; 136.1; 137.3; 140.5; 140.6; 145.2; 145.3; 159.2; 161.9. ESI-MS calcd. for $C_{21}H_{19}N_3O_4S$, 409.46; found: m/z 410.11 $[M + H]^+$. Anal. calcd for $C_{21}H_{19}N_3O_4S$ (C, H, N): C, 61.60; H, 4.68; N, 10.26; found: C, 61.85; H, 4.70; N, 10.30.

Ethyl 5-methoxypyrazolo[1,5-*a*]quinazoline-3-carboxylate (12). To a solution of **11** (0.11 mmol) [42] in 4 mL of methanol, 0.22 mmol of K_2CO_3 was added and the mixture was stirred at reflux for 1.5 hour. After cooling and evaporation of the solvent, 10 mL of water was added and the precipitate was recovered by vacuum filtration to obtain compound **12**. Yield 71%, mp 182-183 °C (EtOH 80%); TLC eluent: toluene/ethyl acetate/methanol 8/2/1.5 v/v/v. ^{1}H-NMR (400 MHz, DMSO-d_6) δ 1.33 (t, 3H, CH_2CH_3, J = 7.2 Hz); 4.20 (s, 3H, OCH_3); 4.27 (q, 2H, CH_2CH_3, J = 7.2 Hz); 7.67 (t, 1H, H_7, J = 7.2 Hz); 8.04 (t, 1H, H_8, J = 7.2 Hz); 8.19 (d, 1H, H_9, J = 7.6 Hz); 8.33 (d, 1H, H_6, J = 8.4 Hz); 8.40 (s, 1H, H_2). ^{13}C-NMR (100 MHz, DMSO-d_6) δ 14.1; 54.5; 60.9; 99.3; 115.7; 116.0; 124.1; 127.1; 127.6; 128.8; 136.1; 145.3; 159.2; 161.8. ESI-MS calcd. for $C_{14}H_{13}N_3O_3$, 271.28; found: m/z 272.10 $[M + H]^+$. Anal. calcd for $C_{14}H_{13}N_3O_3$ (C, H, N): C, 61.99; H, 4.83; N, 15.49; found: C, 61.74; H, 4.81; N, 15.43.

General procedure for isomers 13a/14a. A suspension of 4,5-dihydro-5-oxo-pyrazolo[1,5-*a*]quinazoline-3-carboxyamide **3a** [52] (80 mg, 0.35 mmol) in anhydrous DMF (2.5 mL) and anhydrous Cs_2CO_3 (0.35 mmol) was maintained at room temperature for 15 min. and then methyl iodide (0.70 mmol) was added. The reaction was heated at 80 °C for 1 h. After cooling, ice-cold water was added (20 mL) and the precipitate formed was recovered by vacuum filtration to obtain the O-alkylated compound **13a**. This compound was also obtained starting from **15** (see below). The aqueous phase was extracted with ethyl acetate (3 x 15 mL), then the organic fraction was dried over sodium sulphate, filtered, and the solvent was removed under reduced pressure to obtain the N-alkylated compound **14a**.

5-Methoxypyrazolo[1,5-*a*]quinazoline-3-carboxamide (13a). Yield 80%, mp 246-247 °C (*i*-PrOH); TLC eluent: toluene/ethyl acetate/methanol8/2/1.5 v/v/v. ^{1}H-NMR (400 MHz, DMSO-d_6) δ

4.21 (s, 3H, OCH_3); 7.31 (exch br s, 1H, $CONH_2$); 7.48 (exch br s, 1H, $CONH_2$); 7.66 (t, 1H, H_7, J = 7.2 Hz); 8.04 (t, 1H, H_8, J = 7.2 Hz); 8.19 (d, 1H, H_9, J = 8.0 Hz); 8.31 (m, 2H, H_2, H_6). ^{13}C-NMR (100 MHz, DMSO-d_6) δ 55.5; 106.5; 111.7; 115.0; 126.0; 126.6; 135.9; 137.0; 142.1; 143.9; 160.6; 163.4. ESI-MS calcd. for $C_{12}H_{10}N_4O_2$, 242.24; found: m/z 243.08 $[M + H]^+$. Anal. calcd for $C_{12}H_{10}N_4O_2$ (C, H, N): C, 59.50; H, 4.16; N, 23.13; found: C, 59.74; H, 4.18; N, 23.22.

4-Methyl-5-oxo-4,5-dihydropyrazolo[1,5-*a*]quinazoline-3-carboxamide (14a). Yield 5%, mp 287-289 °C (*i*-PrOH); TLC eluent: toluene/ethyl acetate/methanol8/2/1.5 v/v/v. ^{1}H-NMR (400 MHz, DMSO-d_6) δ 3.74 (s, 3H, NCH_3); 7.34 (exch br s, 1H, $CONH_2$); 7.54 (t, 1H, H_7, J = 7.6 Hz); 7.90 (t, 1H, H_8, J = 7.6 Hz); 7.95 (exch br s, 1H, $CONH_2$); 8.09 (s, 1H, H_2); 8.11 (d, 1H, H_9, J = 8.4 Hz); 8.17 (d, 1H, H_6, J = 7.6 Hz). ^{13}C-NMR (100 MHz, DMSO-d_6) δ 33.2; 115.2; 126.5; 128.7; 135.6; 143.3. ESI-MS calcd. for $C_{12}H_{10}N_4O_2$, 242.24; found: m/z 243.08 $[M + H]^+$. Anal. calcd for $C_{12}H_{10}N_4O_2$ (C, H, N): C, 59.50; H, 4.16; N, 23.13; found: C, 59.74; H, 4.18; N, 23.22.

General procedure for compounds 13b-i and 14c. A suspension of 4,5-dihydro-5-oxo-pyrazolo[1,5-a]quinazoline-3-carboxyamide 3a [52] (150 mg, 0.66 mmol) in anhydrous DMF (3.0 mL) and anhydrous K_2CO_3 (2.64 mmol) was maintained at room temperature for 15 min. and then appropriate substituted benzyl halide (0.99 mmol) was added. The reaction was heated at 50 °C for 2 h. After cooling, ice-cold water was added (20 mL) and the precipitate formed was recovered by vacuum filtration and washed in sequence first with water, then ethanol and diethyl ether to obtained the desired compounds which was purified by crystallization from the suitable solvent.

5-(Benzyloxy)pyrazolo[1,5-*a*]quinazoline-3-carboxamide (13b). From **3a** and benzyl bromide. Yield 65%, mp 208-209 °C (EtOH); IR (nujol) cm^{-1}: 3450, 3420, 1676, 1308; TLC eluent: toluene/ethyl acetate/acetic acid 8/2/1.5 v/v/v. ^{1}H-NMR (400 MHz, DMSO-d_6) δ 5.72 (s, 2H, OCH_2); 7.31 (exch br s, 1H, NH); 7.45 (m, 4H, Ar + NH); 7.60 (d, 2H, Ar, J = 8.0 Hz); 7.68 (t, 1H, H_8, J = 8.0 Hz); 8.07 (t, 1H, H_7, J = 8.0 Hz); 8.25 (d, 1H, H_6, J = 8.4 Hz); 8.34 (s, 1H, H_2); 8.38 (d, 1H, H_9, J = 8.4 Hz). ESI-MS calcd. for $C_{18}H_{14}N_4O_2$, 318.34; found: m/z 319.12 $[M + H]^+$. Anal. calcd for $C_{18}H_{14}N_4O_2$ (C, H, N): C, 67.92; H, 4.43; N, 17.60; found: C, 67.65; H, 4.41; N, 17.53.

5-[(4-(Methylthio)benzyl)oxy]pyrazolo[1,5-*a*]quinazoline-3-carboxamide (13c). From **3a** and 4-(methylthio)benzyl bromide. Yield 94%, mp 225-226 °C (EtOH); TLC: toluene/ethyl acetate/methanol 8/2/1.5 v/v/v. ^{1}H-NMR (400 MHz, DMSO-d$_6$) δ 2.43 (s, 3H, SCH_3); 5.68 (s, 2H, OCH_2); 7.31 (m, 3H, $H_{3'}$, $H_{5'}$, $CONH_2$); 7.44 (exch br s, 1H, $CONH_2$); 7.53 (d, 2H, $H_{2'}$, $H_{6'}$, J = 8.0 Hz); 7.65 (t, 1H, H_7, J = 8.0 Hz); 8.05 (t, 1H, H_8, J = 7.6 Hz); 8.20 (d, 1H, H_9, J = 8.0 Hz); 8.32 (m, 2H, H_2, H_6). ^{13}C-NMR (100 MHz, DMSO-d$_6$) δ 15.0; 69.3; 106.6; 111.8; 115.1; 126.1; 126.3; 126.7; 129.4; 132.7; 136.0; 137.1; 138.9; 142.0; 144.1; 159.9; 163.3. ESI-MS calcd. for $C_{19}H_{16}N_4O_2S$, 364.42; found: m/z 365.10 $[M + H]^+$. Anal. calcd for $C_{19}H_{16}N_4O_2S$ (C, H, N): C, 62.62; H, 4.43; N, 15.37; found: C, 62.37; H, 4.41; N, 15.31.

5-[(2-Chlorobenzyl)oxy]pyrazolo[1,5-*a*]quinazoline-3-carboxamide (13d). From **3a** and 2-chlorobenzyl chloride. Yield 13%, mp 227-229 °C (*i*-PrOH); TLC eluent: toluene/ethyl acetate/acetic acid 9/1/0.5 v/v/v. ^{1}H-NMR (400 MHz, DMSO-d$_6$) δ 5.81 (s, 2H, OCH_2); 7.29 (exch br s, 1H, $CONH_2$); 7.43 (m, 2H, Ar); 7.51 (exch br s, 1H, $CONH_2$); 7.56 (d, 1H, Ar, J = 6.0 Hz); 7.66 (t, 1H, H_7, J = 7.2 Hz); 7.75 (m, 1H, Ar); 8.06 (t, 1H, H_8, J = 7.6 Hz); 8.22 (d, 1H, H_9, J = 7.6 Hz); 8.34 (m, 2H, H_2, H_6). ESI-MS calcd. for $C_{18}H_{13}ClN_4O_2$, 352.78; found: m/z 354.07 $[M + H]^+$. Anal. calcd for $C_{18}H_{13}ClN_4O_2$ (C, H, N): C, 61.28; H, 3.71; N, 15.88; found: C, 61.52; H, 3.72; N, 15.94.

5-[(3-Chlorobenzyl)oxy]pyrazolo[1,5-*a*]quinazoline-3-carboxamide (13e). From **3a** and 2-chlorobenzyl bromide. Yield 81%, mp 242-245 °C (EtOH); TLC eluent: toluene/ethyl acetate/acetic acid 9/1/0.5 v/v/v. ^{1}H-NMR (400 MHz, DMSO-d$_6$) δ 5.73 (s, 2H, OCH_2); 7.28 (exch br s, 1H, $CONH_2$); 7.45 (m, 3H, Ar + $CONH_2$); 7.57 (d, 1H, Ar, J = 6.8 Hz); 7.66 (m, 2H, Ar + H_7); 8.05 (t, 1H, H_8, J = 7.6 Hz); 8.24 (d, 1H, H_9, J = 7.6 Hz); 8.33 (m, 2H, H_2, H_6). ^{13}C-NMR (100 MHz, DMSO-d$_6$) δ 68.6; 115.1; 126.1; 126.7; 127.1; 128.3; 128.6; 130.9; 136.0; 138.8. ESI-MS calcd. for $C_{18}H_{13}ClN_4O_2$, 352.78; found: m/z 354.07 $[M + H]^+$. Anal. calcd for $C_{18}H_{13}ClN_4O_2$ (C, H, N): C, 61.28; H, 3.71; N, 15.88; found: C, 61.52; H, 3.72; N, 15.94.

5-[(4-Bromobenzyl)oxy]pyrazolo[1,5-*a*]quinazoline-3-carboxamide (13f). From **3a** and 4-bromobenzyl bromide. Yield 86%, mp 243-244 °C (EtOH); TLC eluent: toluene/ethyl acetate/acetic

acid 9/1/0.5 v/v/v. ^{1}H-NMR (400 MHz, DMSO-d_6) δ 5.70 (s, 2H, OCH_2); 7.27 (exch br s, 1H, $CONH_2$); 7.44 (exch br s, 1H, $CONH_2$); 7.55 (d, 2H, $H_{2'}$, $H_{6'}$, J = 8.4 Hz); 7.62 (d, 2H, $H_{3'}$, $H_{5'}$, J = 8.4 Hz); 7.66 (t, 1H, H_7, J = 7.6 Hz); 8.05 (t, 1H, H_8, J = 7.6 Hz); 8.23 (d, 1H, H_9, J = 8.0 Hz); 8.32 (m, 2H, H_2, H_6). ^{13}C-NMR (100 MHz, DMSO-d_6) δ 68.8; 106.6; 111.6; 115.0; 121.9; 126.0; 126.7; 129.5; 130.7; 131.7; 131.9; 135.8; 136.0; 137.1; 141,9; 144.0; 159.8; 163.3. ESI-MS calcd. for $C_{18}H_{13}BrN_4O_2$, 397.23; found: m/z 398.02 $[M + H]^+$. Anal. calcd for $C_{18}H_{13}BrN_4O_2$ (C, H, N): C, 54.43; H, 3.30; N, 14.10; found: C, 54.21; H, 3.28; N, 14.04.

5-[(2-Methylbenzyl)oxy]pyrazolo[1,5-*a*]quinazoline-3-carboxamide (13g). From **3a** and 2-methylbenzyl chloride. Yield 34%, mp 226-227 °C (EtOH); TLC eluent: toluene/ethyl acetate/acetic acid 8/2/1 v/v/v. ^{1}H-NMR (400 MHz, DMSO-d_6) δ 2.41 (s, 3H, CH_3); 5.74 (s, 2H, OCH_2); 7.26 (m, 3H, Ar); 7.33 (exch br s, 1H, $CONH_2$); 7.48 (exch br s, 1H, $CONH_2$); 7.54 (d, 1H, Ar, J = 7.2 Hz); 7.65 (t, 1H, H_7, J = 7.6 Hz); 8.05 (t, 1H, H_8, J = 7.6 Hz); 8.18 (d, 1H, H_9, J = 8.0 Hz); 8.33 (m, 2H, H_2, H_6). ^{13}C-NMR (100 MHz, DMSO-d_6) δ 19.1; 68.4; 115.1; 126.0; 126.4; 126.8; 129.1; 129.9; 130.8; 136.0. ESI-MS calcd. for $C_{19}H_{16}N_4O_2$, 332.36; found: m/z 333.13 $[M + H]^+$. Anal. calcd for $C_{19}H_{16}N_4O_2$ (C, H, N): C, 68.66; H, 4.85; N, 16.86; found: C, 68.38; H, 4.83; N, 16.79.

5-[(3-Methoxylbenzyl)oxy]pyrazolo[1,5-*a*]quinazoline-3-carboxamide (13h). From **3a** and 3-methoxylbenzyl bromide. Yield 90%, mp 210-212 °C (EtOH); TLC eluent: toluene/ethyl acetate/acetic acid 8/2/1 v/v/v. ^{1}H-NMR (400 MHz, DMSO-d_6) δ 3.76 (s, 3H, OCH_3); 5.70 (s, 2H, OCH_2); 6.94 (d, 1H, Ar, J = 8.0 Hz); 7.16 (m, 2H, Ar); 7.30 (exch br s, 1H, $CONH_2$); 7.34 (t, 1H, Ar, J = 8.0 Hz); 7.46 (exch br s, 1H, $CONH_2$); 7.67 (t, 1H, H_7, J = 7.6 Hz); 8.05 (t, 1H, H_8, J = 8.0 Hz); 8.24 (d, 1H, H_9, J = 8.0 Hz); 8.33 (m, 2H, H_2, H_6). ^{13}C-NMR (100 MHz, DMSO-d_6) δ 55.5; 69.4; 106.6; 111.8; 114.0; 114.2; 115.2; 120.6; 126.1; 126.7; 130.1; 136.0; 137.1; 137.8; 142.0; 144.1; 159.9; 163.3. ESI-MS calcd. for $C_{19}H_{16}N_4O_3$, 348.36; found: m/z 349.13 $[M + H]^+$. Anal. calcd for $C_{19}H_{16}N_4O_3$ (C, H, N): C, 65.51; H, 4.63; N, 16.08; found: C, 65.77; H, 4.65; N, 16.14.

5-[(4-Sulfamoylbenzyl)oxy]pyrazolo[1,5-*a*]quinazoline-3-carboxamide (13i). From **3a** and 4-(bromomethyl)benzenesulphonamide. Yield 90%, mp 167-168 °C (EtOH); TLC eluent: toluene/ethyl

acetate/acetic acid 8/2/1 v/v/v. ^{1}H-NMR (400 MHz, DMSO-d$_6$) δ 5.81 (s, 2H, OCH$_2$); 7.27 (exch br s, 1H, CONH$_2$); 7.39 (exch br s, 2H, SO$_2$NH$_2$); 7.46 (exch br s, 1H, CONH$_2$); 7.67 (t, 1H, H$_7$, J = 7.6 Hz); 7.77 (d, 2H, H$_{2'}$, H$_{6'}$, J = 8.0 Hz); 7.86 (d, 2H, H$_{3'}$, H$_{5'}$, J = 8.4 Hz); 8.06 (t, 1H, H$_8$, J = 7.6 Hz); 8.27 (d, 1H, H$_9$, J = 8.0 Hz); 8.33 (d, 1H, H$_6$, J = 8.0 Hz); 8.33 (s, 1H, H$_2$). ^{13}C-NMR (100 MHz, DMSO-d$_6$) δ 68.7; 106.0; 111.7; 115.1; 126.2; 126.4; 127.0; 128.6; 136.3; 137.0; 140.5; 142.1; 143.7; 143.9; 160.0; 163.8. ESI-MS calcd. for $C_{18}H_{15}N_5O_4S$, 397.41; found: m/z 398.09 [M + H]$^+$. Anal. calcd for $C_{18}H_{15}N_5O_4S$ (C, H, N): C, 54.40; H, 3.80; N, 17.62; found: C, 54.61; H, 3.81; N, 17.69.

4-[4-(Methylthio)benzyl]-5-oxo-4,5-dihydropyrazolo[1,5-*a*]quinazoline-3-carboxamide (14c). From **3a** and 4-(methylthio)benzyl bromide. Yield 6%, mp 276-279 °C (EtOH); TLC: toluene/ethyl acetate/methanol 8/2/1.5 v/v/v. ^{1}H-NMR (400 MHz, DMSO-d$_6$) δ 2.39 (s, 3H, SCH$_3$); 5.93 (s, 2H, NCH$_2$); 7.10 (m, 4H, Ar); 7.34 (exch br s, 1H, CONH$_2$); 7.57 (t, 1H, H$_7$, J = 8.0 Hz); 7.85 (exch br s, 1H, CONH$_2$); 7.94 (t, 1H, H$_8$, J = 7.2 Hz); 8.12 (s, 1H, H$_2$); 8.16 (d, 1H, H$_9$, J = 8.4 Hz); 8.21 (d, 1H, H$_6$, J = 7.6 Hz). ^{13}C-NMR (100 MHz, DMSO-d$_6$) δ 15.1; 46.5; 115.4; 115.7; 126.4; 126.8; 128.2; 129.0; 134.0; 136.0; 161.1; 164.2. ESI-MS calcd. for $C_{19}H_{16}N_4O_2S$, 364.42; found: m/z 365.10 [M + H]$^+$. Anal. calcd for $C_{19}H_{16}N_4O_2S$ (C, H, N): C, 62.62; H, 4.43; N, 15.37; found: C, 62.37; H, 4.41; N, 15.31.

3-Carbamoylpyrazolo[1,5-*a*]quinazolin-5-yl-4-methylbenzenesulfonate (15). Starting material **3a** [52] (0.31 mmol) was suspended in methylene chloride (10 mL) and 0.70 mmol of 4-toluenesulfonyl chloride and 0.6 mL (in excess) of triethylamine were added. The reaction was maintained at reflux temperature for 2-3 hours, then the solvent was evaporated to dryness. The residue was treated with diethyl ether, filtered and used without further purification to obtain compound **13a** (see above). Yield 85%, mp 246-247 °C (*i*-PrOH); TLC eluent: toluene/ethyl acetate/acetic acid 8/2/1 v/v/v. ^{1}H-NMR (400 MHz, DMSO-d$_6$) δ 2.43 (s, 3H, CH$_3$); 6.72 (exch br s, 1H, CONH$_2$); 7.53 (d, 2H, H$_{3'}$, H$_{5'}$, J = 8.0 Hz); 7.58 (exch br s, 1H, CONH$_2$); 7.76 (t, 1H, H$_7$, J = 7.6 Hz); 8.09 (d, 2H, H$_{2'}$, H$_{6'}$, J = 8.0 Hz); 8.15 (m, 2H, H$_8$, H$_9$); 8.41 (d, 1H, H$_6$, J = 8.4 Hz); 8.47 (s, 1H, H$_2$). Anal. calcd for $C_{18}H_{14}N_4O_4S$ (C, H, N): C, 56.54; H, 3.69; N, 14.65; found: C, 56.76; H, 3.70; N, 14.71.

5-[(4-(Methylsulfinyl)benzyloxy]pyrazolo[1,5-*a*]quinazoline-3-carboxamide (16). To a solution of 176 mg (1 mmol) of HIO_3 in 11 mL of acetone/H_2O (10:1), a small amount of tetra-n-butylammonium bromide (TBAB) was added while stirring for 5 minutes. Then, 0.25 mmol of compound **13c** was added and the mixture was stirred at 80 °C for 2 hours. The reaction mixture was cooled, 10 mL of H_2O was added and the precipitate formed is collected by vacuum filtration. Yield 88%, mp 225-226 °C (EtOH 80%); TLC eluent: toluene/ethyl acetate/acetic acid 8/2/1 v/v/v. ^{1}H-NMR (400 MHz, DMSO-d_6) δ 2.73 (s, 3H, $SOCH_3$); 5.77 (s, 2H, OCH_2); 7.26 (exch brs, 1H, $CONH_2$); 7.46 (exch brs, 1H, $CONH_2$); 7.66 (t, 1H, H_7, J = 7.2 Hz); 7.71 (d, 2H, $H_{2'}$, $H_{6'}$, J = 7.6 Hz); 7.77 (d, 2H, $H_{3'}$, $H_{5'}$, J = 7.6 Hz); 8.04 (t, 1H, H_8, J = 7.2 Hz); 8.25 (d, 1H, H_9, J = 7.6 Hz); 8.30 (m, 2H, H_6, H_2). ^{13}C-NMR (100 MHz, DMSO-d_6) δ 68.9; 106,6; 111.6; 115.0; 124.3; 126.1; 126.7; 129.1; 136.0; 137.1; 139.1; 141.9; 144.0; 146.7; 159.8; 163.3. ESI-MS calcd. for $C_{19}H_{16}N_4O_3S$, 380.42; found: m/z 381.10 $[M + H]^+$. Anal. calcd for $C_{19}H_{16}N_4O_3S$ (C, H, N): C, 59.99; H, 4.24; N, 14.73; found: C, 59.75; H, 4.22; N, 14.67.

5-[(4-(Methylsulfonyl)benzyloxy]pyrazolo[1,5-*a*]quinazoline-3-carboxamide (17). Compound **13c** (0.16 mmol) was dissolved in 4 mL of MeOH. To this solution, at 0 °C, a previously prepared solution composed of 0.64 mmol of OXONE® in 4 mL of H_2O was added. The mixture was stirred at 100 °C for 2 hours. After cooling, 10 mL of H_2O was added and the precipitate formed was recovered by vacuum filtration to obtain the desired compound. Yield 72%, mp 280-281 °C (EtOH); TLC eluent: toluene/ethyl acetate/acetic acid 8/2/1 v/v/v. ^{1}H-NMR (400 MHz, DMSO-d_6) δ 3.23 (s, 3H, SO_2CH_3); 5.85 (s, 2H, OCH_2); 7.23 (exch br s, 1H, $CONH_2$); 7.46 (exch br s, 1H, $CONH_2$); 7.68 (t, 1H, H_7, J = 7.2 Hz); 7.86 (d, 2H, $H_{2'}$, $H_{6'}$, J = 7.6 Hz); 7.97 (d, 2H, $H_{3'}$, $H_{5'}$, J = 7.6 Hz); 8.06 (t, 1H, H_8, J = 7.2 Hz); 8.29 (d, 1H, H_9, J = 8.0 Hz); 8.33 (m, 2H, H_6, H_2). ^{13}C-NMR (100 MHz, DMSO-d_6) δ 47.8; 68.5; 115.1; 126.2; 127.0; 127.7; 128.8; 136.3; 142.4. ESI-MS calcd. for $C_{19}H_{16}N_4O_4S$, 396.42; found: m/z 397.09 $[M + H]^+$. Anal. calcd for $C_{19}H_{16}N_4O_4S$ (C, H, N): C, 57.57; H, 4.07; N, 14.13; found: C, 57.80; H, 4.09; N, 14.19.

(E)-5-(Benzyloxy)-N-[(dimethylamino)methylene]pyrazolo[1,5-*a*]quinazoline-3-carboxamide (18). A suspension of **13b** (150 mg, 0.47 mmol) in toluene (8 mL) was added of DMF-DMA (1.6 mmol, 0.5 mL) and refluxed for 2 hours. The final solution was evaporated to dryness and the yellow residue was recovered with ethyl ether and filtered. The product was pure enough to use in the next reaction. Yield 70%, mp 148-150 °C; IR (nujol) cm^{-1}: 1649, 1305; TLC eluent: toluene/ethyl acetate/acetic acid 8/2/1.5 v/v/v. ^{1}H-NMR (400 MHz, DMSO-d_6) δ 3.17 (s, 3H, N-CH_3); 3.19 (s, 3H, N-CH_3); 5.73 (s, 2H, OCH_2); 7.45 (m, 3H, Ar); 7.63 (d, 2H, Ar, J = 8.0 Hz); 7.68 (t, 1H, H_8, J = 8.0 Hz); 8.04 (t, 1H, H_7, J = 8.0 Hz); 8.20 (d, 1H, H_6, J = 8.4 Hz); 8.35 (d, 1H, H_9, J = 8.4 Hz); 8.46 (s, 1H, H_2); 8.59 (s 1H, CH). ESI-MS calcd. for $C_{21}H_{19}N_5O_2$, 373.42; found: m/z 374.16 $[M + H]^+$. Anal. calcd for $C_{21}H_{19}N_5O_2$ (C, H, N): C, 67.55; H, 5.13; N, 18.76; found: C, 67.82; H, 5.15; N, 18.83.

5-(Benzyloxy)-3-(1H-1,2,4-triazol-5-yl)pyrazolo[1,5-*a*]quinazoline (19). A suspension of **18** (50 mg, 0.13 mmol) in glacial acetic acid (3.0 mL) was added of 60% hydrazine hydrate solution (0.26 mmol, 0.1 mL) and refluxed until a solution was obtained which indicate the end of the reaction. After cooling at room temperature, the precipitate was filtered and washed with water. The residue was purified by recrystallisation. Yield 77%, mp >300 °C (EtOH); IR (nujol) cm^{-1}: 3237, 1310; TLC eluent: toluene/ethyl acetate/acetic acid 8/2/1.5 v/v/v. 1H-NMR (400 MHz, DMSO-d_6) δ 5.83 (s, 2H, OCH_2); 7.45 (m, 3H, Ar); 7.63 (d, 2H, Ar, J = 8.0 Hz); 7.68 (t, 1H, H_8, J = 8.0 Hz); 8.05 (t, 1H, H_7, J = 8.0 Hz); 8.22 (m, 2H, H_6 + CH triazole); 8.36 (d, 1H, H_9, J = 8.4 Hz); 8.49 (s, 1H, H_2); 13.76 (exch br s, 1H, NH). ESI-MS calcd. for $C_{19}H_{14}N_6O$, 342.36; found: m/z 343.13 $[M + H]^+$. Anal. calcd for $C_{19}H_{14}N_6O$ (C, H, N): C, 66.66; H, 4.12; N, 24.55; found: C, 66.39; H, 4.10; N, 24.45.

5-{[4-(Methanesulfonyl)phenyl]methoxy}-3-(thiophen-3-yl)pyrazolo[1,5-*a*]quinazoline (20). Compound 3-(thiophen-3-yl)pyrazolo[1,5-*a*]quinazolin-5(4*H*)-one **6** [37] (0.31 mmol) was suspended in methylene chloride (10 mL) and 0.70 mmol of 4-toluenesulfonyl chloride and 0.6 mL (in excess) of triethylamine were added. The reaction was maintained at reflux temperature for 2-3 hours, then the solvent was evaporated to dryness. The residue was recovered by isopropyl alcohol and crystallised with the same solvent; yield 97%, mp 203 °C; TLC eluent: toluene/ethyl

acetate/acetic acid 8/2/1.5 v/v/v. ^{1}H-NMR (400 MHz, DMSO-d$_6$) δ 2.45 (s, 3H, CH_3); 7.43 (d, 1H, H_2 thiophene, J = 2.8 Hz); 7.45 (d, 1H, H_4 thiophene, J = 5.2 Hz); 7.53 (d, 2H, $H_{3'}$ + $H_{5'}$ J = 8.0 Hz); 7.60 (dd, 1H, H_5 thiophene, J = 2.8 Hz; J = 5.2 Hz); 7.71 (m, 2H, H_7 + H_9); 8.04 (d, 2H, $H_{2'}$ and $H_{6'}$, J = 8.0 Hz); 8.08 (m, 1H, thiophene); 8.13 (t, 1H, H_7, J = 8.0 Hz); 8.37 (d, 1H, H_6, J = 8.4 Hz); 8.60 (s, 1H, H_2). ESI-MS calcd. for $C_{21}H_{15}N_3O_3S_2$, 421.49; found: m/z 422.06 [M + H]$^+$. Anal. calcd for $C_{21}H_{15}N_3O_3S_2$ (C, H, N): C, 59.84; H, 3.59; N, 9.97; found: C, 59.60; H, 3.57; N, 9.93.

General procedure for obtaining compounds 21a,b and 22. To a solution of anhydrous DMF (3 mL), *t*-BuOK (0.28 mmol), the suitable alcohol (0.30 mmol) (benzyl alcohol, for **21a** and 2-aminobenzyl alcohol, for **21b**) was added, and after 15 min. also compound **20** (0.28 mmol), maintaining at reflux temperature for 3 hours. For obtaining compound **22**, to the solution of starting product **20** in DMF was added 0.02 mL of benzylamine and refluxed for 1.30 hours. When starting material disappeared, evaluated by TLC (toluene/ethyl acetate/methanol 8/2/1.5 v/v/v), water and ice were added, and the precipitate was filtered and purified by crystallisation.

5-Benzyloxy-3-(thiophen-3-yl)pyrazolo[1,5-*a*]quinazoline (21a). Starting from compound **20** and benzyl alcohol; yield 36%, mp 179-181 °C (EtOH). ^{1}H-NMR (400 MHz, DMSO-d$_6$) δ 5.71 (s, 2H, OCH_2); 7.35 (m, 1H, thiophene); 7.41 (m, 2H, thiophene + Ar); 7.61 (m, 4H, Ar); 7.79 (m, 1H, thiophene); 7.92 (m, 1H, H_8); 7.97 (m, 1H, H_7); 8.18 (d, 1H, H_9, J = 8.4 Hz); 8.29 (d, 1H, H_6, J = 8.4 Hz); 8.47 (s, 1H, H_2). ESI-MS calcd. for $C_{21}H_{15}N_3OS$, 357.43; found: m/z 358.10 [M + H]$^+$. Anal. calcd for $C_{21}H_{15}N_3OS$ (C, H, N): C, 70.57; H, 4.23; N, 11.76; found: C, 70.29; H, 4.21; N, 11.71.

2-({[3-(Thiophen-3-yl)pyrazolo[1,5-*a*]quinazolin-5-yl]oxy}methyl)aniline (21b). Starting from compound **20** and 2-aminobenzyl alcohol; yield 30%, mp 125-126 °C (EtOH). ^{1}H-NMR (400 MHz, DMSO-d$_6$) δ 4.74 (exch br s, 2H, NH_2); 4.88 (s, 2H, OCH_2); 6.38 (t, 1H, $H_{5'}$ Ar, J = 7.2 Hz); 6.43 (d, 1H, $H_{3'}$ Ar, J = 7.2 Hz); 6.54 (d, 1H, $H_{6'}$ Ar, J = 7.2 Hz); 6.71 (m, 1H, thiophene); 6.89 (t, 1H, $H_{4'}$ Ar, J = 7.2 Hz); 7.04 (m, 1H, thiophene); 7.38 (m, 1H, thiophene); 7.52 (t, 1H, H_7, J = 8.0 Hz); 7.79 (s, 1H, H_2); 7.93 (m, 1H, H_8); 8.18 (m, 2 H, H_9 + H_6). ESI-MS calcd. for $C_{21}H_{16}N_4OS$, 372.45;

found: m/z 373.11 [M + H]$^+$. Anal. calcd for $C_{21}H_{16}N_4OS$ (C, H, N): C, 67.72; H, 4.33; N, 15.04; found: C, 67.99; H, 4.35; N, 15.10.

N-Benzyl-3-(thiophen-3-yl)pyrazolo[1,5-*a*]quinazolin-5-amine (22). Starting from compound **20** and benzyl amine; yield 33%, mp 176-177 °C (EtOH). ^{1}H-NMR (400 MHz, DMSO-d$_6$) δ 4.80 (d, 2H, *CH$_2$*NH); 7.19 (t, 1H, H$_{4'}$ Ar, *J* = 7.2 Hz); 7.30 (t, 2H, H$_{3'}$ + H$_{5'}$ Ar, *J* = 7.6 Hz); 7.45 (d, 2H, H$_{2'}$ + H$_{6'}$ Ar, *J* = 7.6 Hz); 7.50 (m, 1H, H$_{5'}$ thiophene); 7.53 (t, 1H, H$_8$, *J* = 8.4 Hz); 7.64 (d, 1H, H$_{4'}$ thiophene, *J* = 3.6 Hz); 7.79 (dd, 1H, H$_{2'}$ thiophene, *J* = 6.8 Hz, *J* = 2.8 Hz); 7.89 (t, 1H, H$_7$, *J* = 8.0 Hz); 8.21 (d, 1H, H$_9$, *J* = 8.4 Hz); 8.23 (s, 1H, H$_2$); 8.38 (d, 1H, H$_6$, *J* = 8.0 Hz); 8.80 (exch br s, 1H, NH). ESI-MS calcd. for $C_{21}H_{16}N_4S$, 356.45; found: m/z 357.11 [M + H]$^+$. Anal. calcd for $C_{21}H_{16}N_4S$ (C, H, N): C, 70.76; H, 4.52; N, 15.72; found: C, 70.47; H, 4.50; N, 15.66.

General procedure for compounds 23a-c. A suspension of 8-chloro-5-oxo-4,5-dihydropyrazolo[1,5-*a*]quinazoline-3-carboxyamide **3b** (150 mg, 0.66 mmol) in anhydrous DMF (3.0 mL) and anhydrous K_2CO_3 (2.64 mmol) was maintained at room temperature for 15 min. and then appropriate substituted halide (0.99 mmol) was added. The reaction was heated at 50 °C for 2 h. After cooling, ice-cold water was added (20 mL) and the precipitate formed was recovered by vacuum filtration and was purified by flash column chromatography using dichloromethane/methanol 10:0.5 as eluent (for **23a**) or by crystallisation from ethanol (for **23b**) or water/acetic acid 1:1 (for **23c**).

8-Chloro-5-methoxypyrazolo[1,5-*a*]quinazoline-3-carboxamide (23a). From **3b** and methyl iodide. Yield 80%, mp 288-290 °C (EtOH); TLC eluent: dichloromethane/methanol 10/0.5 v/v. ^{1}H-NMR (400 MHz, DMSO-d$_6$) δ 4.21 (s, 3H, OCH$_3$); 7.28 (exch br s, 1H, CONH$_2$); 7.51 (exch br s, 1H, CONH$_2$); 7.70 (dd, 1H, H$_7$, J_1 = 2.0 Hz, J_2 = 8.8 Hz); 8.20 (d, 1H, H$_6$, *J* = 8.8 Hz); 8.29 (d, 1H, H$_9$, *J* = 2.0 Hz); 8.35 (s, 1H, H$_2$). ESI-MS calcd. for $C_{12}H_9ClN_4O_2$, 276.68; found: m/z 278.04 [M + H]$^+$. Anal. calcd for $C_{12}H_9ClN_4O_2$ (C, H, N): C, 52.09; H, 3.28; N, 20.25; found: C, 52.30; H, 3.29; N, 20.33.

8-Chloro-5-[(4-(methylthio)benzyloxy]pyrazolo[1,5-*a*]quinazoline-3-carboxamide (23b). From **3b** and 4-(methylthio)benzyl bromide. Yield 90%, mp 207-209 °C (EtOH); TLC eluent: toluene/ethyl

acetate/acetic acid 8/2/1 v/v/v. ^{1}H-NMR (400 MHz, DMSO-d$_6$) δ 2.42 (s, 3H, SCH_3); 5.61 (s, 2H, OCH_2); 7.30 (m, 3H, $H_{2'}$, $H_{6'}$ + 1H $CONH_2$); 7.47 (exch br s, 1H, $CONH_2$); 7.53 (d, 2H, $H_{3'}$, $H_{5'}$, J = 7.6 Hz); 7.68 (d, 1H, H_7, J = 8.4 Hz); 8.19 (d, 1H, H_6, J = 8.4 Hz); 8.29 (s, 1H, H_9); 8.36 (s, 1H, H_2). ^{13}C-NMR (100 MHz, DMSO-d$_6$) δ 15.05; 69.52; 106.91; 110.58; 114.62; 126.36; 126.96; 128.29; 129.50; 132.53; 137.75; 139.03; 140.55; 142.38; 144.54; 159.55; 163.10. ESI-MS calcd. for $C_{19}H_{15}ClN_4O_2S$, 398.87; found: m/z 400.06 $[M + H]^+$. Anal. calcd for $C_{19}H_{15}ClN_4O_2S$ (C, H, N): C, 57.21; H, 3.79; N, 14.05; found: C, 57.44; H, 3.80; N, 14.10.

8-Chloro-5-(4-sulfamoylbenzyloxy)pyrazolo[1,5-*a*]quinazoline-3-carboxamide (23c). From **3b** and 4-(bromomethyl)benzene sulphonamide. Yield 61%, mp 177-180 °C (H_2O/CH_3COOH); TLC eluent: toluene/ethyl acetate/methanol 8/2/1.5 v/v/v. ^{1}H-NMR (400 MHz, DMSO-d$_6$) δ 5.78 (s, 2H, OCH_2); 7.26 (exch br s, 1H, $CONH_2$); 7.39 (exch br s, 2H, SO_2NH_2); 7.48 (exch br s, 1H, $CONH_2$); 7.69 (dd, 1H, H_7, J_1 = 1.6 Hz, J_2 = 8.4 Hz); 7.75 (d, 2H, $H_{2'}$, $H_{6'}$, J = 8.0 Hz); 7.85 (d, 2H, $H_{3'}$, $H_{5'}$, J = 8.0 Hz); 8.27 (d, 1H, H_6, J = 8.8 Hz); 8.29 (d, 1H, H_9, J = 2.0 Hz); 8.35 (s, 1H, H_2). ^{13}C-NMR (100 MHz, DMSO-d$_6$) δ 68.93; 106.00; 110.68; 114.76; 126.38; 127.07; 128.51; 128.77; 137.50; 140.16; 140.69; 142.10; 143.20; 146.70; 159.20; 163.09. ESI-MS calcd. for $C_{18}H_{14}ClN_5O_4S$, 431.85; found: m/z 433.04 $[M + H]^+$. Anal. calcd for $C_{18}H_{14}ClN_5O_4S$ (C, H, N): C, 50.06; H, 3.27; N, 16.22; found: C, 50.26; H, 3.28; N, 16.28.

8-Chloro-5-[(4-(methylsulfinyl)benzyloxy]pyrazolo[1,5-*a*]quinazoline-3-carboxamide (24). Starting from **23b** (1 mmol), HIO_3 in 11 mL of acetone/H_2O (10:1) and a small amount of tetra-n-butylammonium bromide (TBAB) following the same procedure used for the synthesis of product **16**. The reaction mixture was cooled and 10 mL of ice-cold water was added. The suspension was extracted with ethyl acetate (3 x 15 mL), then the organic fraction was recovered, dried over sodium sulphate, filtered and the solvent was removed under reduced pressure to obtain the desired compound. Yield 63%, mp 240-242 °C (EtOH); TLC eluent: toluene/ethyl acetate/methanol 8/2/1.5 v/v/v. ^{1}H-NMR (400 MHz, DMSO-d$_6$) δ 2.75 (s, 3H, $SOCH_3$); 5.80 (s, 2H, OCH_2); 7.24 (exch br s, 1H, $CONH_2$); 7.47 (exch br s, 1H, $CONH_2$); 7.70 (d, 1H, H_7, J = 8.8 Hz); 7.74 (d, 2H, $H_{2'}$, $H_{6'}$, J =

8.0 Hz); 7.80 (d, 2H, $H_{3'}$, $H_{5'}$, J = 8.0 Hz); 8.26 (d, 1H, H_6, J = 8.8 Hz); 8.31 (s, 1H, H_9); 8.36 (s, 1H, H_2). ESI-MS calcd. for $C_{19}H_{15}ClN_4O_3S$, 414.86; found: m/z 416.05 $[M + H]^+$. Anal. calcd for $C_{19}H_{15}ClN_4O_3S$ (C, H, N): C, 55.01; H, 3.64; N, 13.51; found: C, 55.23; H, 3.65; N, 13.56.

8-Chloro-5-[(4-(methylsulfonyl)benzyloxy]pyrazolo[1,5-*a*]quinazoline-3-carboxamide (25). Starting from **23b** in MeOH and a solution of OXONE® /H_2O, following the same procedure used to synthesise **17**. The row compound was purified by flash column chromatography (dichloromethane/methanol 9.5/0.5 v/v, as eluent). Yield 66%, mp 270-271 °C (EtOH); ^{1}H-NMR (400 MHz, DMSO-d_6) δ 3.23 (s, 3H, SO_2CH_3); 5.85 (s, 2H, OCH_2); 7.22 (exch br s, 1H, $CONH_2$); 7.48 (exch br s, 1H, $CONH_2$); 7.71 (d, 1H, H_7, J = 8.4 Hz); 7.86 (d, 2H, $H_{2'}$, $H_{6'}$, J = 8.0 Hz); 7.98 (d, 2H, $H_{3'}$, $H_{5'}$, J = 7.6 Hz); 8.28 (d, 1H, H_6, J = 8.4 Hz); 8.31 (s, 1H, H_9); 8.36 (s, 1H, H_2). ^{13}C-NMR (100 MHz, DMSO-d_6) δ 43.96; 68.76; 105.77; 115.20; 117.00; 123.00; 124.50; 127.10; 127.73; 128.98; 131.20; 132.30; 140.55; 142.40; 145.00; 153.00; 163.00. ESI-MS calcd. for $C_{19}H_{15}ClN_4O_4S$, 430.86; found: m/z 432.05 $[M + H]^+$. Anal. calcd for $C_{19}H_{15}ClN_4O_4S$ (C, H, N): C, 52.97; H, 3.51; N, 13.00; found: C, 52.75; H, 3.49; N, 12.95.

General procedure for compounds 26 and 27. To a solution of 0.21 mmol of starting material **3b** in 3 mL of anhydrous toluene and 0.3 mL of dry DMF, 0.76 mmol (0.10 mL) of DMF-DMA was added. The reaction was refluxed for 2 h. After cooling, a precipitate was formed and recovered by vacuum filtration to give compound **26**. The evaporation of solvent furnished compound **27**. The same reaction carried out using an excess of DMF-DMA (0.30 mL) led to obtaining only product **27**.

(E)-8-Chloro-N-[(dimethylamino)methylene]-5-oxo-4,5-dihydropyrazolo[1,5-*a*]quinazoline-3-carboxamide (26). Using 0.76 mmol of DMF-DMA, yield 83%, mp 288-290 °C (EtOH); TLC eluent: toluene/ethyl acetate/methanol 8/2/2 v/v/v. ^{1}H-NMR (400 MHz, DMSO-d_6) δ 3.15 (s, 3H, NCH_3); 3.23 (s, 3H, NCH_3); 7.60 (d, 1H, H_7, J = 8.4 Hz); 8.07 (s, 1H, H_9); 8.17 (d, 1H, H_6, J = 8.8 Hz); 8.19 (s, 1H, H_2); 8.67 (s, 1H, N=CH); 10.83 (exch br s, 1H, CONH). ESI-MS calcd. for $C_{14}H_{12}ClN_5O_2$, 317.73; found: m/z 319.07 $[M + H]^+$. Anal. calcd for $C_{14}H_{12}ClN_5O_2$ (C, H, N): C, 52.92; H, 3.81; N, 22.04; found: C, 52.71; H, 3.79; N, 21.95.

(E)-8-Chloro-N-[(dimethylamino)methylene]-4-methyl-5-oxo-4,5-dihydropyrazolo[1,5-*a*]quinazoline-3-carboxamide (27). Using an excess of DMF-DMA, yield 87%, mp 205-207 °C ($EtOH/H_2O$); TLC eluent: toluene/ethyl acetate/methanol 8/2/2 v/v/v. ^{1}H-NMR (400 MHz, DMSO-d_6) δ 3.10 (s, 3H, NCH_3); 3.17 (s, 3H, NCH_3); 3.89 (s, 3H, NCH_3); 7.56 (d, 1H, H_7, J = 7.2 Hz); 8.07 (s, 1H, H_9); 8.15 (d, 1H, H_6, J = 8.4 Hz); 8.35 (s, 1H, H_2); 8.53 (s, 1H, N=CH). ^{13}C-NMR (100 MHz, DMSO-d_6) δ 34.50; 35.54; 41.37; 114.50; 114.93; 125.00; 126.68; 127.51; 131.00; 136.00; 140.22; 143.00; 143.50; 147.00; 160.52. ESI-MS calcd. for $C_{15}H_{14}ClN_5O_2$, 331.76; found: m/z 333.08 $[M + H]^+$. Anal. calcd for $C_{15}H_{14}ClN_5O_2$ (C, H, N): C, 54.31; H, 4.25; N, 21.11; found: C, 54.53; H, 4.26; N, 21.19.

8-Chloro-4-methyl-5-oxo-4,5-dihydropyrazolo[1,5-*a*]quinazoline-3-carbohydrazide (29). 0.26 mmol of starting material **28a** [35] was dissolved in 10 mL of hydrazine hydrate and 2 mL of EtOH 96%. The mixture was refluxed for 7 h. After cooling, a precipitate was formed and recovered by vacuum filtration to obtain the desired compound. Yield 63%, mp >300 °C (2-methoxyethanol); TLC eluent: toluene/ethyl acetate/acetic acid 8/2/1 v/v/v. ^{1}H-NMR (400 MHz, DMSO-d_6) δ 3.66 (s, 3H, NCH_3); 4.46 (exch br s, 2H, NH_2); 7.57 (d, 1H, H_7, J = 8.4 Hz); 8.05 (s, 1H, H_2); 8.08 (s, 1H, H_9); 8.16 (d, 1H, H_6, J = 8.4 Hz); 9.63 (exch br s, 1H, NH). ^{13}C-NMR (100 MHz, DMSO-d_6) δ 32.83; 89.05; 114.23; 114.77; 125.80; 126.77; 131.03; 137.89; 140.00; 143.27; 153.30; 158.53. ESI-MS calcd. for $C_{12}H_{10}ClN_5O_2$, 291.70; found: m/z 293.05 $[M + H]^+$. Anal. calcd for $C_{12}H_{10}ClN_5O_2$ (C, H, N): C, 49.41; H, 3.46; N, 24.01; found: C, 49.60; H, 3.47; N, 24.10.

(E)-8-Chloro-3-(hydrazineylidenemethyl)-4-methylpyrazolo[1,5-*a*]quinazolin-5(4*H*)-one (30b). 0.19 mmol of starting material **28b** [35] was dissolved in 1 mL of hydrazine hydrate and 2 mL of EtOH 96%. The mixture was stirred at reflux for 1 h. After cooling, ice/cold water (20 mL) was added and the precipitate formed was recovered by vacuum filtration to obtain the desired compound. Yield 55%, mp >300 °C (EtOH); TLC eluent: toluene/ethyl acetate/methanol 8/2/1.5 v/v/v. ^{1}H-NMR (400 MHz, DMSO-d_6) δ 3.64 (s, 3H, NCH_3); 6.52 (exch br s, 2H, NH_2); 7.49 (d, 1H, H_7, J = 7.6 Hz); 7.97 (s, 1H, H_9); 8.02 (s, 1H, H_2); 8.10 (d, 1H, H_6, J = 8.0 Hz). ^{13}C-NMR (100 MHz, DMSO-d_6) δ 31.88;

114.25; 114.70; 126.05; 131.00; 131.09; 137.90; 138.07; 140.17; 141.78; 146.30; 158.50. ESI-MS calcd. for $C_{12}H_{10}ClN_5O$, 275.70; found: m/z 277.05 [M + H]$^+$. Anal. calcd for $C_{12}H_{10}ClN_5O$ (C, H, N): C, 52.28; H, 3.66; N, 25.40; found: C, 52.49; H, 3.67; N, 25.50.

(*E*)-8-Chloro-4-methyl-5-oxo-4,5-dihydropyrazolo[1,5-*a*]quinazoline-3-carbaldehyde oxime (30c). A suspension of **28b** (0.19 mmol) [35] and 0.57 mmol of hydroxylammonium chloride in 2 mL of H_2O was stirred at 60 °C for 30 minutes, then 0.57 mmol of $NaHCO_3$ was added and the mixture was refluxed for 2.5 hours. After cooling, ice/cold water (20 mL) was added and the precipitate formed was recovered by vacuum filtration. The desired compound was purified using by flash chromatography with cyclohexane/ethyl acetate 1/1 v/v as eluent. Yield 50%, mp 264-267 °C; ^{1}H-NMR (400 MHz, DMSO-d$_6$) δ 3.07 (s, 3H, NCH_3); 7.55 (d, 1H, H_7, J = 8.0 Hz); 8.05 (s, 1H, H_9); 8.12 (s, 1H, H_2); 8.15 (d, 1H, H_6, J = 7.6 Hz); 8.38 (s, 1H, *CH*NOH); 11.05 (exch br s, 1H, NOH). ESI-MS calcd. for $C_{12}H_9ClN_4O_2$, 276.68; found: m/z 278.04 [M + H]$^+$. Anal. calcd for $C_{12}H_9ClN_4O_2$ (C, H, N): C, 52.09; H, 3.28; N, 20.25; found: C, 52.30; H, 3.29; N, 20.33.

8-Chloro-4-methyl-3-{[(4-(methylthio)benzyloxy]methyl}pyrazolo[1,5-*a*]quinazolin-5(4*H*)-one (31). A solution of intermediate **30a** (0.23 mmol) [35] and 0.70 mmol of sodium hydride (60% dispersion in mineral oil) in 2 mL of dry CH_3CN was stirred at room temperature for 20 minutes, then 0.35 mmol of 4-methylthiobenzyl bromide was added and the mixture was stirred at reflux for 24 hours. After cooling, the solvent was evaporated and the solid obtained was washed with water. The crude compound was purified by flash chromatography using toluene/ethyl acetate/acetic acid 9/1/0.5 v/v/v as eluent. Yield 11%, mp 132-133 °C; ^{1}H-NMR (400 MHz, $CDCl_3$) δ 2.48 (s, 3H, SCH_3); 3.82 (s, 3H, NCH_3); 4.53 (s, 2H, CH_2); 4.58 (s, 2H, CH_2); 7.25 (m, 4H, Ar); 7.38 (dd, 1H, H_7, J_1 = 1.6 Hz, J_2 = 8.8 Hz); 7.70 (s, 1H, H_2); 8.18 (d, 1H, H_9, J = 1.6 Hz); 8.23 (d, 1H, H_6, J = 8.4 Hz). ^{13}C-NMR (100 MHz, $CDCl_3$) δ 15.77; 30.42; 36.62; 62.04; 71.41; 101.51; 114.03; 114.94; 126.17; 126.52; 128.66; 129.48; 130.50; 134.12; 137.89; 138.35; 138.87; 141.33; 144.90; 158.57. ESI-MS calcd. for $C_{20}H_{18}ClN_3O_2S$, 399.89; found: m/z 401.08 [M + H]$^+$. Anal. calcd for $C_{20}H_{18}ClN_3O_2S$ (C, H, N): C, 60.07; H, 4.54; N, 10.51; found: C, 60.31; H, 4.56; N, 10.55.

N-(4-Aminophenyl)-8-chloro-4-methyl-5-oxo-4,5-dihydropyrazolo[1,5-*a*]quinazoline-3-carboxamide (32). A solution of starting material **28c** (0.36 mmol) [35] in 3 mL of $SOCl_2$ was stirred at reflux for 2 hours. After cooling, the solvent was evaporated and the solid obtained was dissolved in 2 mL of dry CH_2Cl_2. To this solution, 1.08 mmol of benzene-1,4-diamine and 1.08 mmol of triethylamine were added and the mixture was stirred at reflux temperature for 4 hours. After cooling, the solvent was evaporated and the final compound was purified by flash column chromatography using dichloromethane/methanol 9.5/0.5 v/v as eluent. Yield 20%, mp 155-157 °C; ^{1}H-NMR (400 MHz, DMSO-d_6) δ 3.58 (s, 3H, NCH_3); 5.01 (exch br s, 1H, NH_2); 6.57 (d, 2H, $H_{3'}$, $H_{5'}$, J = 8.0 Hz); 7.28 (d, 2H, $H_{2'}$, $H_{6'}$, J = 8.0 Hz); 7.56 (d, 1H, H_7, J = 8.0 Hz); 8.08 (s, 1H, H_9); 8.12 (s, 1H, H_2); 10.03 (exch br s, 1H, NH). ESI-MS calcd. for $C_{18}H_{14}ClN_5O_2$, 367.79; found: m/z 369.08 $[M + H]^+$. Anal. calcd for $C_{18}H_{14}ClN_5O_2$ (C, H, N): C, 58.78; H, 3.84; N, 19.04; found: C, 58.54; H, 3.82; N, 18.96.

General procedure for compounds 33a, b. The starting material **3c** or **3d** (0.35 mmol) were treated in anhydrous DMF (2.5 mL), K_2CO_3 (0.35 mmol) and stirred at room temperature for 15 min. then methyl iodide (0.70 mmol) was added. The reaction was heated at 80 °C for 1 h and, after cooling, ice-cold water was added (20 mL), and the precipitate formed was recovered by vacuum filtration. The crude compounds were purified by flash column chromatography using dichloromethane/methanol/acetic acid 97/3/03 v/v/v (for **33a**) or toluene/ethyl acetate/acetic acid 8/2/1 v/v/v (for **33b**) as eluents.

5-Methoxy-8-nitropyrazolo[1,5-*a*]quinazoline-3-carboxamide (33a). From **3c**, yield 58%, mp > 300 °C; TLC eluent: dichloromethane/methanol/acetic acid 97/3/03 v/v/v. ^{1}H-NMR (400 MHz, DMSO-d_6) δ 4.25 (s, 3H, CH_3); 7.27 (exch br s, 1H, NH_2); 7.56 (exch br s, 1H, NH_2); 8.36 (dd, 1H, H_2, J_1 = 2.0 Hz, J_2 = 8.8 Hz); 8.42 (m, 2H, H_7, H_6); 8.88 (d, 1H, H_9, J = 1.6 Hz). ^{13}C-NMR (400 MHz, DMSO-d_6) δ 56.1; 110.5; 120.5; 128.8; 131.2; 144.9. ESI-MS calcd. for $C_{12}H_9N_5O_4$, 287.24; found: m/z 288.07 $[M + H]^+$. Anal. calcd for $C_{12}H_9N_5O_4$ (C, H, N): C, 50.18; H, 3.16; N, 24.38; found: C, 50.38; H, 3.17; N, 24.47.

5-Methoxy-7-nitropyrazolo[1,5-*a*]quinazoline-3-carboxamide (33b). From **3d,** yield 40%, mp 269-271 °C; TLC eluent: toluene/ethyl acetate/acetic acid 8/2/1 v/v/v. ^{1}H-NMR (400 MHz, DMSO-d_6) δ 4.26 (s, 3H, CH_3); 7.28 (exch br s, 1H, NH_2); 7.58 (exch br s, 1H, NH_2); 8.44 (s, 1H, H_2); 8.47 (d, 1H, H_9, J = 9.2 Hz); 8.77 (dd, 1H, H_8, J_1 = 2.4 Hz, J_2 = 9.2 Hz); 8.86 (d, 1H, H_6, J = 2.4 Hz). ^{13}C-NMR (400 MHz, DMSO-d_6) δ 56.1; 107.4; 112.1; 117.1; 122.3; 130.1; 140.2; 144.8; 145.7; 160.4; 163.0. ESI-MS calcd. for $C_{12}H_9N_5O_4$, 287.24; found: m/z 288.07 $[M + H]^+$. Anal. calcd for $C_{12}H_9N_5O_4$ (C, H, N): C, 50.18; H, 3.16; N, 24.38; found: C, 50.38; H, 3.17; N, 24.47.

5-Methoxy-8-nitropyrazolo[1,5-*a*]quinazoline-3-carbonitrile (34). A solution of **33a** (0.27 mmol) in 3 mL of $POCl_3$ was stirred at 80 °C for 2 hours. After cooling and evaporation of the solvent, ice/cold water (20 mL) was added and the solid obtained was recovered by vacuum filtration e purified by recrystallisation. Yield 92%, mp >300 °C (EtOH); TLC eluent: toluene/ethyl acetate/methanol 8/2/2 v/v/v. ^{1}H-NMR (400 MHz, DMSO-d_6) δ 4.24 (s, 3H, CH_3); 8.41 (dd, 1H, H_7, J_1 = 2.2 Hz, J_2 = 9.0 Hz); 8.46 (d, 1H, H_6, J = 8.8 Hz); 8.68 (s, 1H, H_2); 8.89 (d, 1H, H_9, J = 2.0 Hz). ^{13}C-NMR (400 MHz, DMSO-d_6) δ 56.15; 83.08; 110.68; 114.91; 116,42; 121.28; 128.90; 136.87; 146.35; 148.05; 151.31; 161.18. ESI-MS calcd. for $C_{12}H_7N_5O_3$, 269.22; found: m/z 270.06 $[M + H]^+$. Anal. calcd for $C_{12}H_7N_5O_3$ (C, H, N): C, 53.54; H, 2.62; N, 26.01; found: C, 53.75; H, 2.63; N, 26.11.

8-Nitro-5-oxo-4,5-dihydropyrazolo[1,5-*a*]quinazoline-3-carbaldehyde (35). To a solution of 0.43 mmol of starting material **5c** in 5 mL of glacial acetic acid, 4.30 mmol of hexamethylenetetramine (HTMA) was added and the reaction was stirred at reflux temperature for 3 hours. After cooling, water was added, the suspension was extracted with ethyl acetate (3 x 15 mL), dried over anhydrous sodium sulphate, filtered, and finally evaporated to obtain the desired compound. Yield 73%, mp 150-153 °C (EtOH); TLC eluent: toluene/ethyl acetate/methanol 8/2/2 v/v/v. ^{1}H-NMR (400 MHz, DMSO-d_6) δ 8.30-8.42 (m, 3H, H_2, H_7, H_6); 8.67 (s, 1H, H_9); 9.99 (s, 1H, CHO). ESI-MS calcd. for $C_{11}H_6N_4O_4$, 258.19; found: m/z 259.04 $[M + H]^+$. Anal. calcd for $C_{11}H_6N_4O_4$ (C, H, N): C, 51.17; H, 2.34; N, 21.70; found: C, 51.37; H, 2.35; N, 21.78.

(E)-8-Nitro-5-oxo-4,5-dihydropyrazolo[1,5-*a*]quinazoline-3-carbaldehyde oxime (36). Starting material **35** was reacted with hydroxylammonium chloride following the same procedure for obtaining **30c**. At the end of the reaction, the suspension was extracted with ethyl acetate (3 x 15 mL), dried over sodium sulphate, filtered and finally evaporated. The final compound was purified by flash column chromatography, using cyclohexane/ethyl acetate 1/5 v/v as eluent. Yield 65%, mp 243-246 °C; ^{1}H-NMR (400 MHz, DMSO-d_6) δ 7.65 (s, 1H, H_2); 8.24 (d, 1H, H_6, J = 8.4 Hz); 8.36 (d, 1H, H_7, J = 8.4 Hz); 8.49 (s, 1H, CH); 8.65 (s, 1H, H_9); 11.49 (exch br s, 1H, OH). ESI-MS calcd. for $C_{11}H_7N_5O_4$, 273.21; found: m/z 274.05 $[M + H]^+$. Anal. calcd for $C_{11}H_7N_5O_4$ (C, H, N): C, 48.36; H, 2.58; N, 25.63; found: C, 48.16; H, 2.57; N, 25.52.

4-Methyl-8-nitro-5-oxo-4,5-dihydropyrazolo[1,5-*a*]quinazoline-3-carbonitrile (37). Starting material **2c** was reacted in anhydrous DMF, K_2CO_3, and methyl iodide following the procedure used for obtaining **33a,b**. Yield 94%, mp 260-263 °C dec. (EtOH); TLC eluent: toluene/ethyl acetate/methanol 8/2/2 v/v/v. ^{1}H-NMR (400 MHz, DMSO-d_6) δ 3.74 (s, 3H, CH_3); 8.32 (dd, 1H, H_7, J_1 = 8.6 Hz, J_2 = 2.2 Hz); 8.44 (d, 1H, H_9, J = 8.8); 8.54 (s, 1H, H_2); 8.69 (d, 1H, H_6, J = 2.0 Hz). ^{13}C-NMR (400 MHz, DMSO-d_6) δ 37.30; 77.47; 110.84; 113.99; 120.81; 121.42; 131.47; 136.89; 144.26; 146.24; 151.66; 157.48. ESI-MS calcd. for $C_{12}H_7N_5O_3$, 269.22; found: m/z 270.06 $[M + H]^+$. Anal. calcd for $C_{12}H_7N_5O_3$ (C, H, N): C, 53.54; H, 2.62; N, 26.01; found: C, 53.75; H, 2.63; N, 26.11.

4-Methyl-8-nitro-5-oxo-4,5-dihydropyrazolo[1,5-*a*]quinazoline-3-carboxamide (38). Starting compound **37** (0.82 mmol) was solubilised in 2 mL of H_2SO_4 conc., and stirred at 80 °C for 2 hours. After cooling, the mixture was slowly poured into water and ice. The precipitate formed was isolated by vacuum filtration to obtain the desired product. Yield 89%, mp 286-289 °C (EtOH); TLC eluent: toluene/ethyl acetate/methanol 8/2/2 v/v/v. ^{1}H-NMR (400 MHz, DMSO-d_6) δ 3.74 (s, 3H, CH_3); 7.39 (exch br s, 1H, NH); 7.93 (exch br s, 1H, NH); 8.19 (s, 1H, H_2); 8.25 (dd, 1H, H_7, J_1 = 2.2 Hz, J_2 = 8.6 Hz); 8.39 (d, 1H, H_6, J = 8.4 Hz); 8.70 (d, 1H, H_9 J = 2.0 Hz). ^{13}C-NMR (400 MHz, DMSO-d_6) δ 37.04; 104.2; 110.34; 111.00; 120.20; 121.25; 131.28; 137.40; 139.81; 151.49; 158.29; 163.77. ESI-

MS calcd. for $C_{12}H_9N_5O_4$, 287.24; found: m/z 288.07 $[M + H]^+$. Anal. calcd for $C_{12}H_9N_5O_4$ (C, H, N): C, 50.18; H, 3.16; N, 24.38; found: C, 50.38; H, 3.17; N, 24.47.

General procedure for compounds 39a-c. To a solution of 0.39 mmol di suitable starting material (**2c, 2d,** or **4d**) in 2.7 mL of $POCl_3$, 3.60 mmol of PCl_5 was added. The mixture was stirred at 200 °C for 2.5-4.5 hours. After cooling to room temperature, ice/cold water (20 mL) was added and the precipitate formed was recovered by vacuum filtration to obtain the desired compounds.

5-Chloro-8-nitropyrazolo[1,5-*a*]quinazoline-3-carbonitrile (39a). From **2c**, yield 75%, mp > 295 °C (EtOH); TLC eluent: toluene/ethyl acetate/acetic acid 8/2/1 v/v/v. ^{1}H-NMR (400 MHz, DMSO-d$_6$) δ 8.52 (dd, 1H, H_7, J_1 = 2.0 Hz, J_2 = 8.8 Hz); 8.63 (d, 1H, H_6, J = 9.2 Hz); 8.92 (s, 1H, H_2); 9.02 (d, 1H, H_9, J = 2.4 Hz). ESI-MS calcd. for $C_{11}H_4ClN_5O_2$, 273.64; found: m/z 275.00 $[M + H]^+$. Anal. calcd for $C_{11}H_4ClN_5O_2$ (C, H, N): C, 48.28; H, 1.47; N, 25.59; found: C, 48.47; H, 1.47; N, 25.69.

5-Chloro-7-nitropyrazolo[1,5-*a*]quinazoline-3-carbonitrile (39b). From **2d**, yield 84%, mp 222-224 °C (EtOH); TLC eluent: toluene/ethyl acetate/acetic acid 8/2/1 v/v/v. ^{1}H-NMR (400 MHz, DMSO-d$_6$) δ 8.66 (d, 1H, H_9, J = 9.2 Hz); 8.90 (dd, 1H, H_8, J_1 = 2.4 Hz, J_2 = 9.2 Hz); 8.93 (s, 1H, H_2); 9.01 (d, 1H, H_6, J = 2.0 Hz). ESI-MS calcd. for $C_{11}H_4ClN_5O_2$, 273.64; found: m/z 275.00 $[M + H]^+$. Anal. calcd for $C_{11}H_4ClN_5O_2$ (C, H, N): C, 48.28; H, 1.47; N, 25.59; found: C, 48.47; H, 1.47; N, 25.69.

Ethyl 5-chloro-7-nitropyrazolo[1,5-*a*]quinazoline-3-carboxylate (39c). From **4d**, yield 88%, mp 169-172 °C (EtOH); TLC eluent: toluene/ethyl acetate/methanol 8/2/2 v/v/v. ^{1}H-NMR (400 MHz, DMSO-d$_6$) δ 1.32 (t, 3H, CH_3, J = 7.0 Hz); 4.33 (q, 2H, CH_2, J = 7.1 Hz); 8.65 (d, 1H, H_9, J = 9.2 Hz); 8.72 (s, 1H, H_2); 8.87 (dd, 1H, H_8, J_1 = 2.4 Hz, J_2 = 9.2 Hz); 8.98 (d, 1H, H_6, J = 2.4 Hz). ESI-MS calcd. for $C_{13}H_9ClN_4O_4$, 320.69; found: m/z 322.03 $[M + H]^+$. Anal. calcd for $C_{13}H_9ClN_4O_4$ (C, H, N): C, 48.69; H, 2.83; N, 17.47; found: C, 48.49; H, 2.82; N, 17.40.

General procedure for compounds 40 and 42b-d. To a suspension of 0.29 mmol of suitable starting material **39a-c** in 5.0 mL of isopropyl alcohol, 0.58 mmol of substituted aniline and a catalytic amount of triethylamine (3 gt) were added. The mixture was stirred at reflux for 2-20 hours (the reaction was

monitored by TLC until the starting materials disappeared). After cooling, a precipitate was obtained, recovered by vacuum filtration and purified by crystallisation from suitable solvent.

Ethyl 5-[(4-aminophenyl)amino]-7-nitropyrazolo[1,5-*a*]quinazoline-3-carboxylate (40). Starting from **39c** and 1,4-phenylenediamine. Yield 94%, mp 269-271 °C (2-methoxyethanol); TLC eluent: dichloromethane/methanol 10/1 v/v. ^{1}H-NMR (400 MHz, DMSO-d$_6$) δ 1.32 (t, 3H, CH_3, J = 7.0 Hz); 4.23 (q, 2H, CH_2, J = 7.0 Hz); 5.05 (exch br s, 2H, NH_2); 6.61 (d, 2H, $H_{3'}$, $H_{5'}$, J = 8.8 Hz); 7.88 (d, 2H, $H_{2'}$, $H_{6'}$, J = 8.8 Hz); 8.32 (s, 1H, H_2); 8.40 (d, 1H, H_9, J = 8.8 Hz); 8.67 (dd, 1H, H_8, J_1 = 2.4 Hz, J_2 = 9.2 Hz); 9.68 (d, 1H, H_6, J = 2.0 Hz); 10,08 (exch br s, 1H, NH). ^{13}C-NMR (400 MHz, DMSO-d$_6$) δ 15.1; 59.8; 101.1; 112.4; 113.9; 117.0; 162.8; 122.2; 123.2; 128.6; 128.7; 139.7; 144.8; 146.0; 146.5; 146.8; 151.3. ESI-MS calcd. for $C_{19}H_{16}N_6O_4$, 392.38; found: m/z 393.13 $[M + H]^+$. Anal. calcd for $C_{19}H_{16}N_6O_4$ (C, H, N): C, 58.16; H, 4.11; N, 21.42; found: C, 58.39; H, 4.13; N, 21.50.

5-[(4-(methylthiophenyl)amino]-8-nitropyrazolo[1,5-*a*]quinazoline-3-carbonitrile (42b). Starting from **39a** and 4-(methylthio)aniline. Yield 73%, mp > 295 °C (*i*-PrOH); IR (nujol) cm^{-1}: 3120, 2240, 1440-1360; TLC eluent: toluene/ethyl acetate/methanol 8/2/2 v/v/v. ^{1}H-NMR (400 MHz, DMSO-d$_6$) δ 2.52 (s, 3H, CH_3); 7.34 (d, 2H, $H_{3'}$, $H_{5'}$, J = 8.4 Hz); 7.71 (exch br s, 1H, NH); 7.80 (s, 2H, $H_{2'}$, $H_{6'}$, J = 8.8 Hz); 8.17 (m, 2H, H_2, H_6); 8.38 (dd, 1H, H_7, J_1 = 1.6 Hz, J_2 = 8.8 Hz); 9.26 (d, 1H, H_9, J = 2.0 Hz). ESI-MS calcd. for $C_{18}H_{12}N_6O_2S$, 376.39; found: m/z 377.08 $[M + H]^+$. Anal. calcd for $C_{18}H_{12}N_6O_2S$ (C, H, N): C, 57.44; H, 3.21; N, 22.33; found: C, 57.66; H, 3.22; N, 22.42.

5-[(4-Aminophenyl)amino]-8-nitropyrazolo[1,5-*a*]quinazoline-3-carbonitrile (42c). Starting from **39a** and 1,4-phenylenediamine. Yield 79%, mp >295 °C (2-methoxyethanol); TLC eluent: dichloromethane/methanol 9/1 v/v. ^{1}H-NMR (400 MHz, DMSO-d$_6$) δ 5.10-5.40 (exch br s, 2H, NH_2); 6.61 (m, 2H, $H_{3'}$, $H_{5'}$); 7.43 (m, 2H, $H_{2'}$, $H_{6'}$); 8.37 (m, 2H, H_2, H_6); 8.78 (m, 2H, H_9, H_7); 10.11 (exch br s, 1H, NH). ESI-MS calcd. for $C_{17}H_{11}N_7O_2$, 345.32; found: m/z 346.10 $[M + H]^+$. Anal. calcd for $C_{17}H_{11}N_7O_2$ (C, H, N): C, 59.13; H, 3.21; N, 28.39; found: C, 59.36; H, 3.22; N, 28.50.

5-[(4-Aminophenyl)amino]-7-nitropyrazolo[1,5-*a*]quinazoline-3-carbonitrile (42d). Starting from **39b** and 1,4-phenylenediamine. Yield 61%, mp >295 °C (2-methoxyethanol); IR (nujol) cm^{-1}:

3350; 3300; 2220; 1450-1350; TLC eluent: dichloromethane/methanol 9/1 v/v. ^{1}H-NMR (400 MHz, DMSO-d$_6$) δ 7.28 (d, 2H, H$_{3'}$, H$_{5'}$, J = 8.4 Hz); 7.85 (d, 2H, H$_{2'}$, H$_{6'}$, J = 8.4 Hz); 8.46 (d, 1H, H$_9$, J = 9.2 Hz); 8.53 (s, 1H, H$_2$); 8.76 (dd, 1H, H$_8$, J_1 = 2.0 Hz, J_2 = 9.2 Hz); 9.00-9.50 (exch br s, 2H, NH$_2$); 9.72 (d, 1H, H$_6$, J = 1.6 Hz); 10.61 (exch br s, 1H, NH). ^{13}C-NMR (400 MHz, DMSO-d$_6$) δ 79.7; 112.8; 114.1; 114.5; 117.1; 122.4; 124.9; 127.2; 129.0; 139.5; 145.0; 146.5; 146.6; 150.2; 152.7. ESI-MS calcd. for $C_{17}H_{11}N_7O_2$, 345.32; found: m/z 346.10 [M + H]$^+$. Anal. calcd for $C_{17}H_{11}N_7O_2$ (C, H, N): C, 59.13; H, 3.21; N, 28.39; found: C, 59.36; H, 3.22; N, 28.50.

Ethyl 5-{[4-(cyclopropanecarboxamido)phenyl]amino}-7-nitropyrazolo[1,5-*a*]quinazoline-3-carboxylate (41). A cold solution (0 °C) of 0.13 mmol of compound **40** in 2.0 mL of dry CH_2Cl_2 added of cyclopropane carbonyl chloride (0.32 mmol) and a catalytic amount of triethylamine (0.03 mL), was stirred for 2 hours, and then at room temperature for another 2 hours. The precipitate formed was recovered by vacuum filtration to obtain the desired compound. Yield 85%, mp >295 °C (2-methoxyethanol); TLC eluent: toluene/ethyl acetate/acetic acid 8/2/1 v/v/v. ^{1}H-NMR (400 MHz, DMSO-d$_6$) δ 0.78 (m, 4H, 2 x CH$_2$ cC$_3$H$_5$); 1.35 (t, 3H, CH$_3$, J = 7.0 Hz); 1.77 (m, 1H, CH cC$_3$H$_5$); 4.27 (q, 2H, CH$_2$, J = 7.0 Hz); 7.63 (d, 2H, H$_{2'}$, H$_{6'}$, J = 8.8 Hz); 8.19 (d, 2H, H$_{3'}$, H$_{5'}$, J = 8.8 Hz); 8.36 (s, 1H, H$_2$); 8.43 (d, 1H, H$_9$, J = 9.2 Hz); 8.70 (dd, 1H, H$_8$, J_1 = 2.0 Hz, J_2 = 9.2 Hz); 9.75 (d, 1H, H$_6$, J = 1.6 Hz); 10.20 (exch br s, 1H, NH); 10,24 (exch br s, 1H, NH). ^{13}C-NMR (400 MHz, DMSO-d$_6$) δ 6.9; 14.1; 14.4; 59.3; 108.0; 115.1; 116.7; 118.7; 119.3; 122.1; 127.1; 128.5; 130.5; 136.5; 145.5; 146.6; 161.8; 165.0; 172.0: 180.7. ESI-MS calcd. for $C_{23}H_{20}N_6O_2$, 460.45; found: m/z 461.15 [M + H]$^+$. Anal. calcd for $C_{23}H_{20}N_6O_2$ (C, H, N): C, 60.00; H, 4.38; N, 18.25; found: C, 60.24; H, 4.40; N, 18.32.

5-(Methylamino)-8-nitropyrazolo[1,5-*a*]quinazoline-3-carbonitrile (42a). To a solution of 0.25 mmol of starting material **39a** in 4.0 mL of 1,4-dioxane, 0.76 mmol of methylamine and 0.38 mmol of N,N-diisopropylethylamine (DIPEA) were added. The mixture was stirred at room temperature for 1.5 hours. Then, ice/cold water (20 mL) was added and the precipitate obtained was recovered by vacuum filtration to obtain the desired compound. Yield 67%, mp >300 °C (EtOH); TLC eluent:

toluene/ethyl acetate/methanol 8/2/2 v/v/v. ^{1}H-NMR (400 MHz, DMSO-d$_6$) δ 3.06 (d, 3H, CH$_3$ *J* = 4.4 Hz); 8.37 (d, 1H, H$_7$, *J* = 2.0 Hz); 8.39 (s, 1H, H$_2$); 8.54 (d, 1H, H$_6$, *J* = 8.8 Hz); 8.75 (d, 1H, H$_9$, *J* = 2.0 Hz); 8.98 (exch br d, 1H, NH). ^{13}C-NMR (400 MHz, DMSO-d$_6$) δ 28.7; 66.4; 114.5; 116.1; 119.9; 121.8; 125.5; 135.7; 146.1; 150.2; 154.4; 163.2. ESI-MS calcd. for $C_{12}H_8N_6O_2$, 268.24; found: m/z 269.07 [M + H]$^+$. Anal. calcd for $C_{12}H_8N_6O_2$ (C, H, N): C, 53.73; H, 3.01; N, 31.33; found: C, 53.51; H, 2.99; N, 31.20.

5-(Methylamino)-8-nitropyrazolo[1,5-*a*]quinazoline-3-carboxamide (43). Starting compound **42a** (0.82 mmol) was transformed into the corresponding carboxamide following the same procedure for obtaining compound **38**. Yield 70%, mp > 300 °C (EtOH); TLC eluent: toluene/ethyl acetate/methanol 8/2/2 v/v/v. ^{1}H-NMR (400 MHz, DMSO-d$_6$) δ 3.04 (s, 3H, CH$_3$); 7.24 (exch br s, 1H, NH); 7.48 (exch br s, 1H, NH); 8.14 (s, 1H, H$_2$); 8.30 (d, 1H, H$_7$, *J* = 8.4 Hz); 8.52 (d, 1H, H$_6$, *J* = 8.8 Hz); 8.74 (s, 2H, H$_9$ + NH). ESI-MS calcd. for $C_{12}H_{10}N_6O_3$, 286.25; found: m/z 287.08 [M + H]$^+$. Anal. calcd for $C_{12}H_{10}N_6O_3$ (C, H, N): C, 50.35; H, 3.52; N, 29.36; found: C, 50.55; H, 3.53; N, 29.47.

5-[(4-(Methylsulfinylphenyl)amino]-8-nitropyrazolo[1,5-*a*]quinazoline-3-carbonitrile (44). Starting compound **42b** was oxidated with HIO_3 in acetone/H_2O (10:1) following the procedure used for synthesising **24**. Yield 72%, mp 241-243 °C (EtOH); TLC eluent: toluene/ethyl acetate/methanol 8/2/2 v/v/v. ^{1}H-NMR (400 MHz, DMSO-d$_6$) δ 2.77 (s, 3H, SOCH$_3$); 7.76 (d, 2H, H$_{2'}$, H$_{6'}$, *J* = 7.6 Hz); 8.05 (d, 2H, H$_{3'}$, H$_{5'}$, *J* = 6.0 Hz); 8.44 (m, 2H, H$_2$, H$_6$); 8.81 (m, 2H, H$_9$, H$_7$); 10.48 (exch br s, 1H, NH). ESI-MS calcd. for $C_{18}H_{12}N_6O_3S$, 392.39; found: m/z 393.07 [M + H]$^+$. Anal. calcd for $C_{18}H_{12}N_6O_3S$ (C, H, N): C, 55.10; H, 3.08; N, 21.42; found: C, 55.32; H, 3.09; N, 21.50.

5-[(4-(methylsulfonylphenyl)amino]-8-nitropyrazolo[1,5-*a*]quinazoline-3-carbonitrile (45). Starting compound **42b** was oxidated with OXONE® following the same procedure used to synthesise **17**. Yield 69%, mp 187-189 °C (EtOH); TLC eluent: toluene/ethyl acetate/methanol 8/2/2 v/v/v. ^{1}H-NMR (400 MHz, DMSO-d$_6$) δ 3.24 (s, 3H, SO$_2$CH$_3$); 7.98 (d, 2H, H$_{2'}$, H$_{6'}$, *J* = 8.4 Hz); 8.20 (d, 2H, H$_{3'}$, H$_{5'}$, *J* = 8.4 Hz); 8.52 (m, 1H, H$_6$); 8.58 (s, 1H, H$_2$); 8.90 (s, 1H, H$_9$); 8.98 (d, 1H, H$_7$, *J* = 8.4

Hz); 10.59 (exch br s, 1H, NH). ESI-MS calcd. for $C_{18}H_{12}N_6O_4S$, 408.39; found: m/z 409.07 [M + H]$^+$. Anal. calcd for $C_{18}H_{12}N_6O_4S$ (C, H, N): C, 52.94; H, 2.96; N, 20.58; found: C, 53.15; H, 2.97; N, 20.66.

General procedure for compounds 46a-c. To a cold solution (0 °C) of 0.11 mmol of suitable starting material (**33a**, **42a** or **43**) in 2 mL of HCl 37%, 1.10 mmol of tin powder was added. The reaction was stirred at 0 °C for 30 minutes. The suspension was then filtered off on a celite pad, and the pH of the filtrate was adjusted to 9-10 with NaOH 6N. The precipitate formed was collected by vacuum filtration to obtain the desired compound. The final compounds **46a-c** were purified by crystallisation.

8-Amino-5-methoxypyrazolo[1,5-*a*]quinazoline-3-carboxamide (46a). Starting from **33a**. Yield 98%, mp >300 °C (EtOH); TLC eluent: dichloromethane/methanol 8/2 v/v. ^{1}H-NMR (400 MHz, DMSO-d$_6$) δ 4.09 (s, 3H, OCH_3); 6.79 (m, 3H, H_7 + NH_2 exch); 7.23 (s, 1H, H_9); 7.33 (exch br s, 2H, NH_2); 7.80 (d, 1H, H_6, J = 8.8 Hz); 8.18 (s, 1H, H_2). ^{13}C-NMR (400 MHz, DMSO-d$_6$) δ 54.0; 94.0; 99.1; 104.2; 114.7; 127.5; 139.3; 143.1; 143.2; 155.8; 160.7; 163.8. ESI-MS calcd. for $C_{12}H_{11}N_5O_2$, 257.25; found: m/z 258.09 [M + H]$^+$. Anal. calcd for $C_{12}H_{10}N_6O_3$ (C, H, N): C, 56.03; H, 4.31; N, 27.22; found: C, 56.25; H, 4.33; N, 27.33.

8-Amino-5-(methylamino)pyrazolo[1,5-*a*]quinazoline-3-carbonitrile (46b). Starting from **42a**. Yield 76%, mp >300 °C (EtOH); TLC eluent: toluene/ethyl acetate/methanol 8/2/2 v/v/v. ^{1}H-NMR (400 MHz, DMSO-d$_6$) δ 2.96 (s, 3H, CH_3); 6.48 (exch br s, 2H, NH_2); 6.73 (d, 1H, H_7, J = 8.8 Hz); 7.17 (s, 1H, H_9); 8.00 (d, 1H, H_6, J = 8.8 Hz); 8.17 (s, 1H, H_2); 8.27 (exch br s,1H, NH). ESI-MS calcd. for $C_{12}H_{10}N_6$, 238.25; found: m/z 239.10 [M + H]$^+$. Anal. calcd for $C_{12}H_{10}N_6$ (C, H, N): C, 60.50; H, 4.23; N, 35.27; found: C, 60.26; H, 4.21; N, 35.12.

8-Amino-5-(methylamino)pyrazolo[1,5-*a*]quinazoline-3-carboxamide (46c). Starting from **43**. Yield 66%, mp >300 °C (EtOH); TLC eluent: toluene/ethyl acetate/methanol 8/2/2 v/v/v. ^{1}H-NMR (400 MHz, DMSO-d$_6$) δ 2.96 (s, 3H, CH_3); 6.41 (exch br s, 2H, NH_2); 6.73 (m, 1H, H_7); 7.06 (s, 1H, H_9); 7.21 (exch br s, 1H, NH); 7.67 (exch br s, 1H, NH); 7.95 (m, 1H, H_6); 7.99 (exch br s, 1H, NH);

8.14 (s, 1H, H_2). ESI-MS calcd. for $C_{12}H_{12}N_6O$, 256.27; found: m/z 257.11 $[M + H]^+$. Anal. calcd for $C_{12}H_{12}N_6O$ (C, H, N): C, 56.24; H, 4.72; N, 32.79; found: C, 56.46; H, 4.74; N, 32.92.

8-Amino-5-methoxypyrazolo[1,5-*a*]quinazoline-3-carbonitrile (47). Starting material **46a** was dehydrated with $POCl_3$ following the same procedure used to synthesise **34**. Yield 72%, mp >300 °C (EtOH); TLC eluent: toluene/ethyl acetate/methanol 8/2/2 v/v/v. ^{1}H-NMR (400 MHz, DMSO-d_6) δ 4.08 (s, 3H, OCH_3); 6.83 (s, 3H, H_7, NH_2, exch); 7.23 (s, 1H, H_9); 7.80 (d, 1H, H_6, J = 8.0 Hz); 8.42 (s, 1H, H_2). ^{13}C-NMR (400 MHz, DMSO-d_6) δ 54.5; 80.6; 100.8; 114.6; 115.7; 127.4; 134.3; 139.1; 145.0; 148.2; 156.0; 161.7. ESI-MS calcd. for $C_{12}H_9N_5O$, 239.24; found: m/z 240.08 $[M + H]^+$. Anal. calcd for $C_{12}H_9N_5O$ (C, H, N): C, 60.25; H, 3.79; N, 29.27; found: C, 60.49; H, 3.80; N, 29.39.

General procedure for compounds 48 and 49. Starting compounds **37** and **38** were reduced with tin powder, as reported for synthesizing **46a-c**.

8-Amino-4-methyl-5-oxo-4,5-dihydropyrazolo[1,5-*a*]quinazoline-3-carbonitrile (48). Starting from **37**. Yield 94%, mp 286-288 °C (EtOH); TLC eluent: toluene/ethyl acetate/methanol 8/2/2 v/v/v. ^{1}H-NMR (400 MHz, DMSO-d_6) δ 3.65 (s, 3H, CH_3); 6.66 (exch br s, 2H, NH_2); 6.71 (d, 1H, H_7, J = 8.8 Hz); 7.11 (s, 1H, H_9); 7.80 (d, 1H, H_6, J = 8.8 Hz); 8.33 (s, 1H, H_2). ^{13}C-NMR (400 MHz, DMSO-d_6) δ 37.3; 77.5; 96.0; 103.9; 114.8; 118.0; 125.0; 130.5; 142.9; 145.0; 150.0; 155.8. ESI-MS calcd. for $C_{12}H_9N_5O$, 239.24; found: m/z 240.08 $[M + H]^+$. Anal. calcd for $C_{12}H_9N_5O$ (C, H, N): C, 60.25; H, 3.79; N, 29.27; found: C, 60.49; H, 3.80; N, 29.38.

8-Amino-4-methyl-5-oxo-4,5-dihydropyrazolo[1,5-*a*]quinazoline-3-carboxamide (49). Starting from **38**. Yield 74%, mp > 300 °C (EtOH); TLC eluent: dichloromethane/methanol 8/2 v/v. ^{1}H-NMR (400 MHz, DMSO-d_6) δ 3.64 (s, 3H, CH_3); 6.48 (exch br s, 2H, NH_2); 6.65 (d, 1H, H_7, J = 8.0 Hz); 7.12 (s, 1H, H_9); 7.20 (exch br s, 1H, NH_2); 7.79 (d, 2H, H_6 + 1H NH_2, J = 8.8 Hz); 8.02 (s, 1H, H_2). ^{13}C-NMR (400 MHz, DMSO-d_6) δ 32.7; 96.0; 103.1; 103.8; 113.6; 130.3; 138.8; 139.6; 142.9; 155.4; 158.9; 164.3. ESI-MS calcd. for $C_{12}H_{11}N_5O_2$, 257.25; found: m/z 258.09 $[M + H]^+$. Anal. calcd for $C_{12}H_{11}N_5O_2$ (C, H, N): C, 56.03; H, 4.31; N, 27.22; found: C, 56.25; H, 4.33; N, 27.32.

Ethyl 7-[(4-methylphenyl)sulfonamido]-6-oxo-6,7-dihydropyrazolo[1,5-*a*]pyrido[3,4-*e*]pyrimi--dine-3-carboxylate (51a). A mixture of diethyl 7-[(*E*)-2-(dimethylamino)ethenyl]pyrazolo[1,5-a]pyrimidine-3,6-dicarboxylate **50** [38] (0.01 mmol) and 4-tolylsolphonylhydrazide (0.015 mmol) was heated to reflux under magnetic stirring in glacial acetic acid (20 mL) for 3 hours. On cooling, the precipitate was filtered, washed and recrystallised by ethanol; yield 97 %, mp 203 °C (EtOH); TLC eluent: toluene/ethyl acetate/acetic acid 8/2/1.5 v/v/v. ^{1}H-NMR (400 MHz, DMSO-d_6) δ 1.34 (t, 3H, CH_2CH_3, J = 7.2 Hz); 2.42 (s, 3H, CH_3); 4.35 (q, 2H, CH_2CH_3, J = 7.2 Hz); 7.25 (d, 1H, H_9, J = 8.0 Hz); 7.37 (d, 2H, $H_{3'}$, $H_{5'}$, J = 8.4 Hz); 7.65 (d, 2H, $H_{2'}$, $H_{6'}$, J = 8.4 Hz); 8.21 (d, 1H, H_8, J = 8.0 Hz); 8.78 (s, 1H, H_2); 9.04 (s, 1H, H_5); 11.96 (exch br s, 1H, NH). ESI-MS calcd. for $C_{19}H_{17}N_5O_5S$, 427.44; found: m/z 428.10 $[M + H]^+$. Anal. calcd for $C_{19}H_{17}N_5O_5S$ (C, H, N): C, 53.39; H, 4.01; N, 16.38; found: C, 53.60; H, 4.03; N, 16.44.

General procedure for compounds 53a,b. The commercial starting materials ethyl 7-amino-6-cyanopyrazolo[1,5-*a*]pyrimidine-3-carboxylate **52a** or 7-amino-6-cyanopyrazolo[1,5-*a*]pyrimidine **52b** (2 mmol) were suspended in formamide (15 mL) and refluxed for 8 hours. The reaction was monitored by TLC (dichloromethane/methanol 9:1, v/v as eluent) until the starting materials disappeared. The cooling to room temperature yielded a precipitate separated by filtration and crystallised from a suitable solvent.

Ethyl 4-oxo-1,4-dihydropyrazolo[1,5-*a*]pyrimido[5,4-*e*]pyrimidine-7-carboxylate (53a). Yield 55 %, mp >300 °C (CH_3COOH). ^{1}H-NMR (400 MHz, DMSO-d_6) δ 1.20 (t, 3H, CH_3, J = 7.2 Hz); 4.14 (q, 2H, CH_2, J = 7.2 Hz); 8.54 (s, 1H, H_2); 8.58 (s, 1H, H_8); 9.37 (s, 1H, H_5). ESI-MS calcd. for $C_{11}H_9N_5O_3$, 259.23; found: m/z 260.07 $[M + H]^+$. Anal. calcd for $C_{11}H_9N_5O_3$ (C, H, N): C, 50.97; H, 3.50; N, 27.02; found: C, 50.76; H, 3.48; N, 26.91.

Pyrazolo[1,5-*a*]pyrimido[5,4-*e*]pyrimidin-4(1*H*)-one (53b). Yield 38 %, mp >300 °C (2-methoxyehanol). ^{1}H-NMR (400 MHz, DMSO-d_6) δ 6.84 (d, 1H, H_3, J = 2.0 Hz); 8.24 (d, 1H, H_2, J = 2.0 Hz); 8.36 (exch br s, 1H, NH); 8.58 (s, 1H, H_8); 9.20 (s, 1H, H_5). ESI-MS calcd. for $C_8H_5N_5O$,

187.16; found: m/z 188.05 [M + H]$^+$. Anal. calcd for $C_8H_5N_5O$ (C, H, N): C, 51.34; H, 2.69; N, 37.42; found: C, 51.54; H, 2.70; N, 38.42.

Ethyl (*Z*)-3-[(4-phenyl-1*H*-pyrazol-5-yl)amino]-2-(1*H*-pyrazol-1-yl)acrylate (56). A mixture of 3-amino-4-phenylpyrazole **54** (1 mmol) and ethyl 2-(pyrazol-1'-yl)-2-formylacetate **55** (1 mmol) [39] was heated to reflux under magnetic stirring in diglyme (20 mL) for 3 hours. Evaporation of the solvent gave a residue which was purified by recrystallisation; yield 80 %, mp 152-155 °C (EtOH/H_2O); TLC eluent: dichloromethane/methanol 10/1 v/v; from TLC is evidenced the formation of the two isomers, cis and trans. The reported ^{1}H-NMR data are related to the isomer in high quantity. ^{1}H-NMR (400 MHz, DMSO-d_6) δ 1.40 (t, 3H, CH_3, J = 7.2 Hz); 4.35 (q, 2H, CH_2, J = 7.2 Hz); 6.58 (t, 1H, $H_{4'}$ pyrazole, J = 2.4 Hz); 7.26 (m, 1H, Ar); 7.58-7.63 (m, 4H, Ar); 7.83 (d, 1H, $H_{5'}$ pyrazole, J = 1.6 Hz); 8.15 (s, 1H, H_3 pyrazole); 8.22 (d, 1H, $H_{3'}$ pyrazole, J = 2.4 Hz); 8.35 (d, 1H, -C=CH, J = 12.4 Hz); 9.81 (exch br d, 1H, =C-NH, J = 12.4 Hz); 12.80 (exch br s, 1H, NH pyrazole). ESI-MS calcd. for $C_{17}H_{17}N_5O_2$, 323.36; found: m/z 324.14 [M + H]$^+$. Anal. calcd for $C_{17}H_{17}N_5O_2$ (C, H, N): C, 63.15; H, 5.30; N, 21.66; found: C, 63.40; H, 5.32; N, 21.75.

2. Chemical structures of all tested compounds (Tables S1-S3)

Table S1

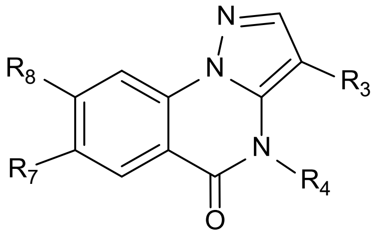

Comp.	R_3	R_4	R_7	R_8
**2c**	CN	H	H	NO_2
**2d**	CN	H	NO_2	H
**3a** [34]	$CONH_2$	H	H	H
**4c**	COOEt	H	H	NO_2
**4d**	COOEt	H	NO_2	H
**4e** [36]	COOEt	H	H	OCH_3
**7a** [30]	COOEt	CH_3	H	H
**7b**	COOEt	$CH_2(4\text{-}SCH_3)Ph$	H	H
**7c**	COOEt	$CH_2(4\text{-}SO_2NH_2)Ph$	H	H
**7d**	COOEt	$CH_2(4\text{-}SO_2CH_3)Ph$	H	H
**7e**	CN	$CH_2(4\text{-}SCH_3)Ph$	H	H
**7f**	H	$CH_2(4\text{-}SCH_3)Ph$	H	H
**8**	H	$CH_2(4\text{-}SO_2CH_3)Ph$	H	H
**9**	I	$CH_2(4\text{-}SOCH_3)Ph$	H	H
**10**	COOEt	$CH_2(4\text{-}SOCH_3)Ph$	H	H
**14c**	$CONH_2$	$CH_2(4\text{-}SCH_3)Ph$	H	H
**26**	$CON{=}CHN(CH_3)_2$	H	H	Cl
**27**	$CON{=}CHN(CH_3)_2$	CH_3	H	Cl
**29**	$CONHNH_2$	CH_3	H	Cl
**30a** [35]	CH_2OH	CH_3	H	Cl
**30b**	$CH{=}NNH_2$	CH_3	H	Cl
**30c**	CH=NOH	CH_3	H	Cl
**31**	$CH_2OCH_2(4\text{-}SCH_3)Ph$	CH_3	H	Cl
**32**	$CONH(4\text{-}NH_2)Ph$	CH_3	H	Cl
**36**	CH=NOH	H	H	NO_2
**37**	CN	CH_3	H	NO_2
**38**	$CONH_2$	CH_3	H	NO_2
**48**	CN	CH_3	H	NH_2
**49**	$CONH_2$	CH_3	H	NH_2

Table S2

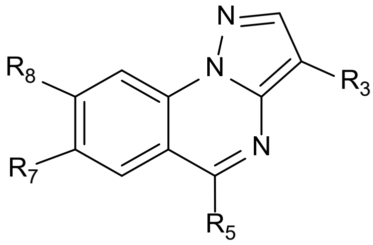

**Comp.**	**R3**	**R5**	**R7**	**R8**
**12**	COOEt	OCH_3	H	H
**13a**	$CONH_2$	OCH_3	H	H
**13b**	$CONH_2$	OCH_2Ph	H	H
**13c**	$CONH_2$	$OCH_2(4\text{-}SCH_3)Ph$	H	H
**13d**	$CONH_2$	$OCH_2(2\text{-}Cl)\text{-}Ph$	H	H
**13e**	$CONH_2$	$OCH_2(3\text{-}Cl)\text{-}Ph$	H	H
**13f**	$CONH_2$	$OCH_2(4\text{-}Br)\text{-}Ph$	H	H
**13g**	$CONH_2$	$OCH_2(2\text{-}CH_3)\text{-}Ph$	H	H
**13h**	$CONH_2$	$OCH_2(3\text{-}OCH_3)\text{-}Ph$	H	H
**13i**	$CONH_2$	$OCH_2(4\text{-}SO_2NH_2)\text{-}Ph$	H	H
**16**	$CONH_2$	$OCH_2(4\text{-}SOCH_3)Ph$	H	H
**17**	$CONH_2$	$OCH_2(4\text{-}SO_2CH_3)Ph$	H	H
**19**	1,2,4-triazol-3-yl	OCH_2Ph	H	H
**20**	3-thienyl	$OSO_2(4\text{-}Me)Ph$	H	H
**21a**	3-thienyl	OCH_2Ph	H	H
**21b**	3-thienyl	$OCH_2(2\text{-}NH_2)Ph$	H	H
**22**	3-thienyl	$NHCH_2Ph$	H	H
**23a**	$CONH_2$	OCH_3	H	Cl
**23b**	$CONH_2$	$OCH_2(4\text{-}SCH_3)Ph$	H	Cl
**23c**	$CONH_2$	$OCH_2(4\text{-}SO_2NH_2)\text{-}Ph$	H	Cl
**24**	$CONH_2$	$OCH_2(4\text{-}SOCH_3)Ph$	H	Cl
**25**	$CONH_2$	$OCH_2(4\text{-}SO_2CH_3)Ph$	H	Cl
**33a**	$CONH_2$	OCH_3	H	NO_2
**33b**	$CONH_2$	OCH_3	NO_2	H
**34**	CN	OCH_3	H	NO_2
**40**	COOEt	$NH(4\text{-}NH_2)Ph$	NO_2	H
**41**	COOEt	NH(4-NHCO-*c*-Pr)Ph	NO_2	H
**42a**	CN	$NHCH_3$	H	NO_2
**42b**	CN	$NH(4\text{-}SCH_3)Ph$	H	NO_2
**42c**	CN	$NH(4\text{-}NH_2)Ph$	H	NO_2
**42d**	CN	$NH(4\text{-}NH_2)Ph$	NO_2	H
**43**	$CONH_2$	$NHCH_3$	H	NO_2
**44**	CN	$NH(4\text{-}SOCH_3)Ph$	H	NO_2
**45**	CN	$NH(4\text{-}SO_2CH_3)Ph$	H	NO_2
**46a**	$CONH_2$	OCH_3	H	NH_2
**46b**	CN	$NHCH_3$	H	NH_2
**46c**	$CONH_2$	$NHCH_3$	H	NH_2
**47**	CN	OCH_3	H	NH_2
**57** [42]	$CONHCH_2(2\text{-}OCH_3)Ph$	H	H	OCH_3

Table S3

	Compound	R_3	R_7
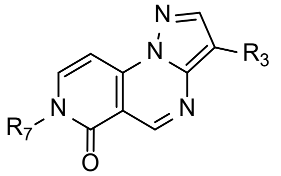	**51a**	COOEt	$-SO_2$-Ph-4-CH_3
	**51b** [38]	CN	-NHCONH$_2$
	**51c** [38]	CN	-NHCO-Ph-4-NO$_2$
	**51d** [38]	COOEt	-NHCONH$_2$
	**51e** [29]	CN	-OH
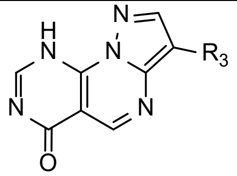	**53a**	COOEt	-
	**53b**	H	-
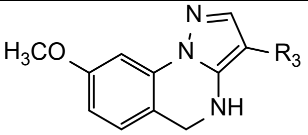	**58a** [42]	CONH$_2$	-
	**58b** [42]	CONHCH$_2$-2-Thienyl	-
	**58c** [42]	CONHCH$_2$-Ph	-

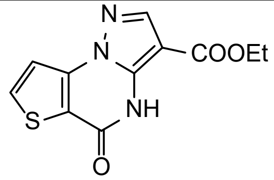 **59** [30]	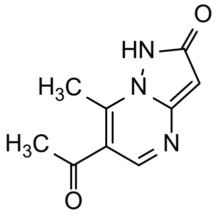 **60** [31]
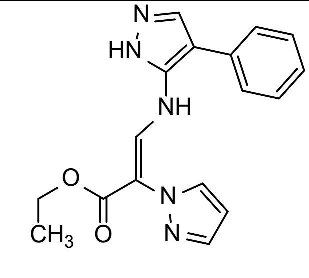 **56**	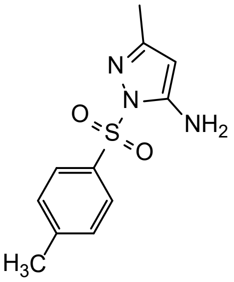 **61** [32]

3. ^{1}H-NMR and ^{13}C-NMR of representative compounds

Figure S1. ^{1}H NMR of Compound 7b.

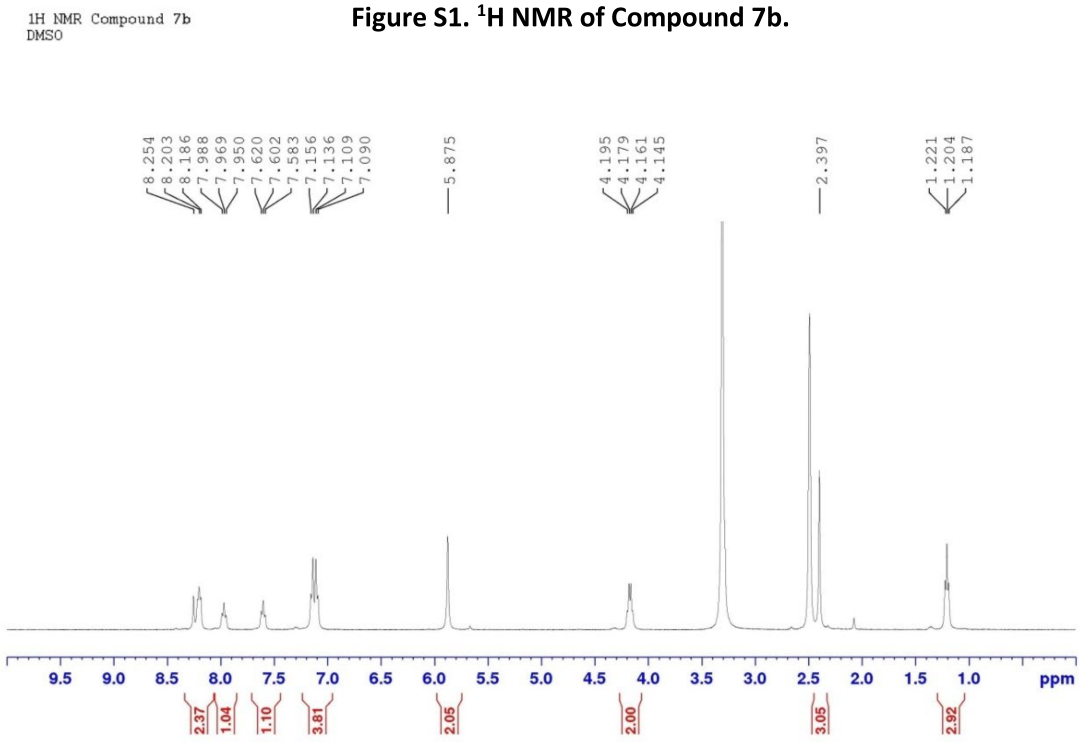

Figure S2. ^{13}C NMR of Compound 7b.

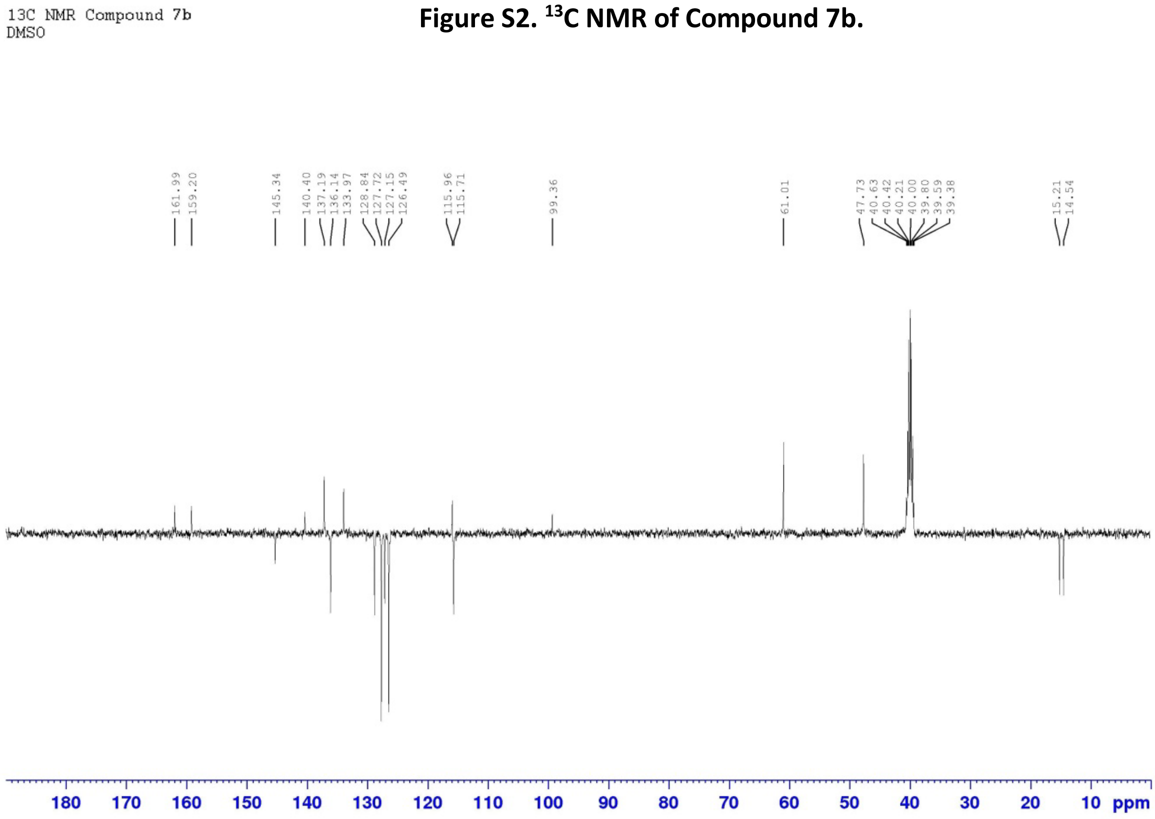

Figure S3. ^{1}H NMR of Compound 7c.

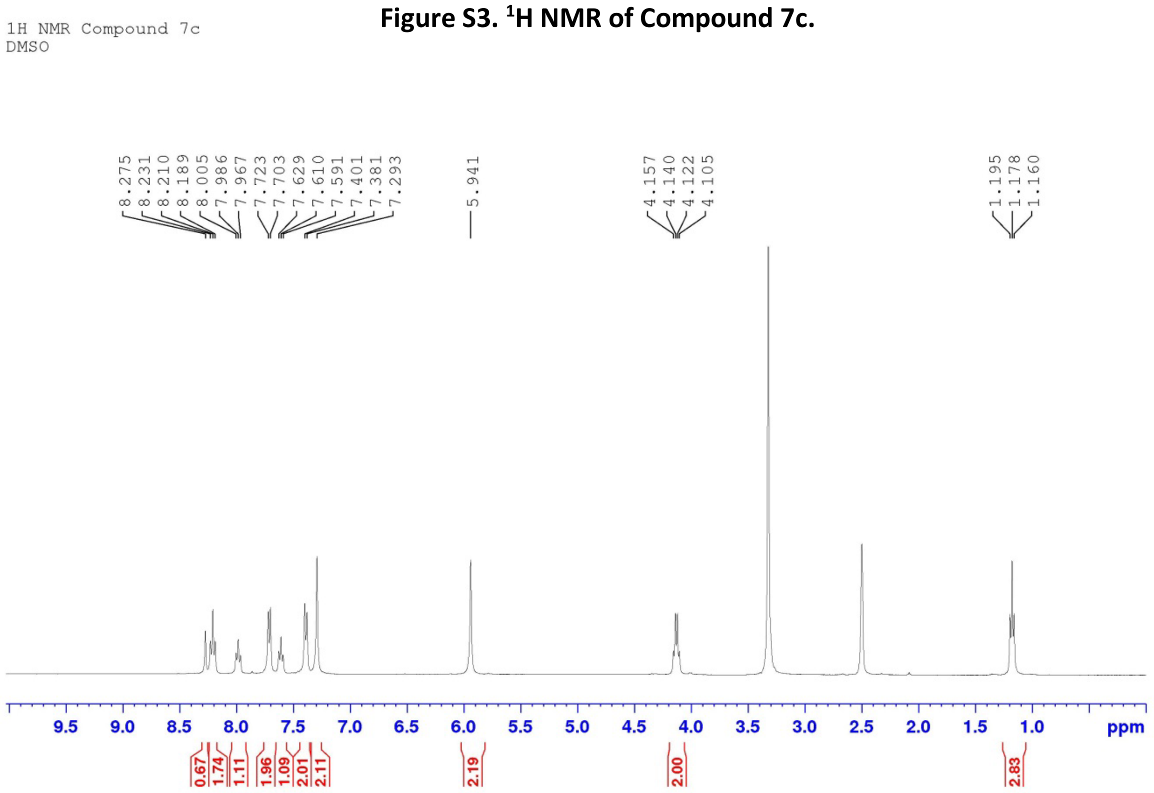

Figure S4. ^{13}C NMR of Compound 7c.

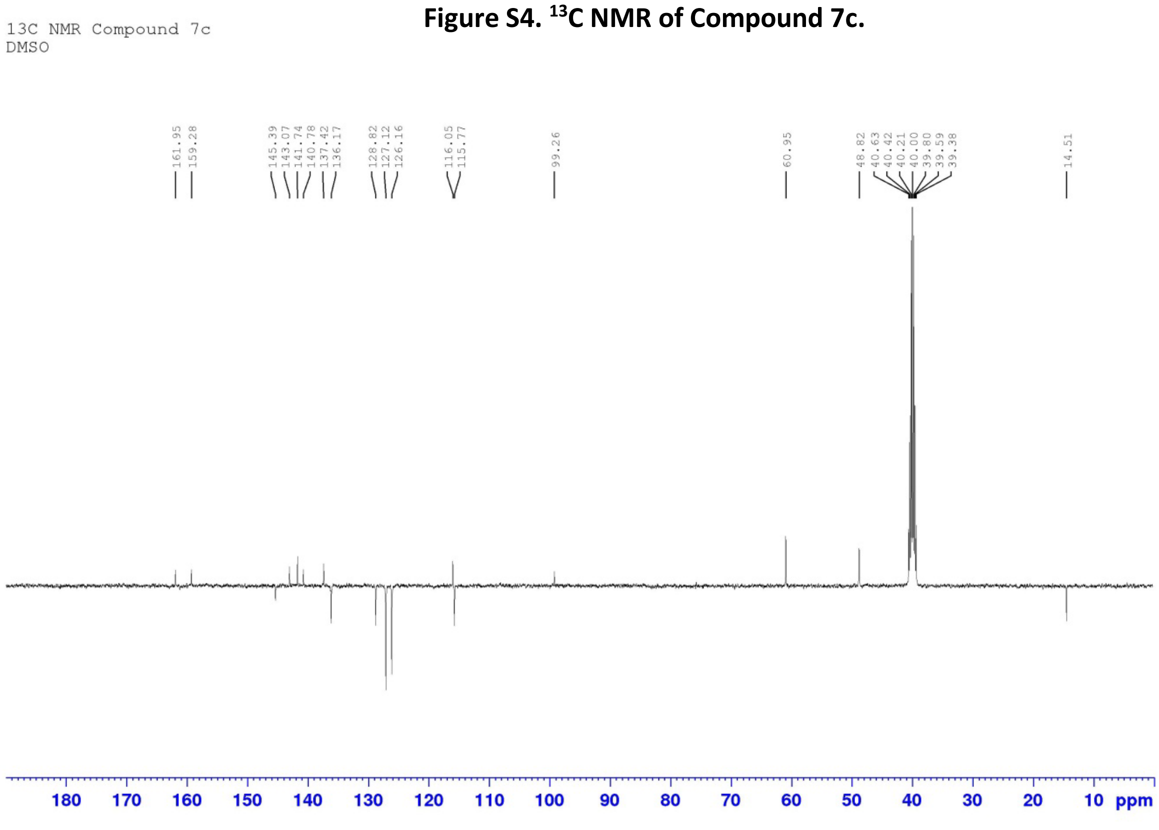

Figure S5. ^{1}H NMR of Compound 7d.

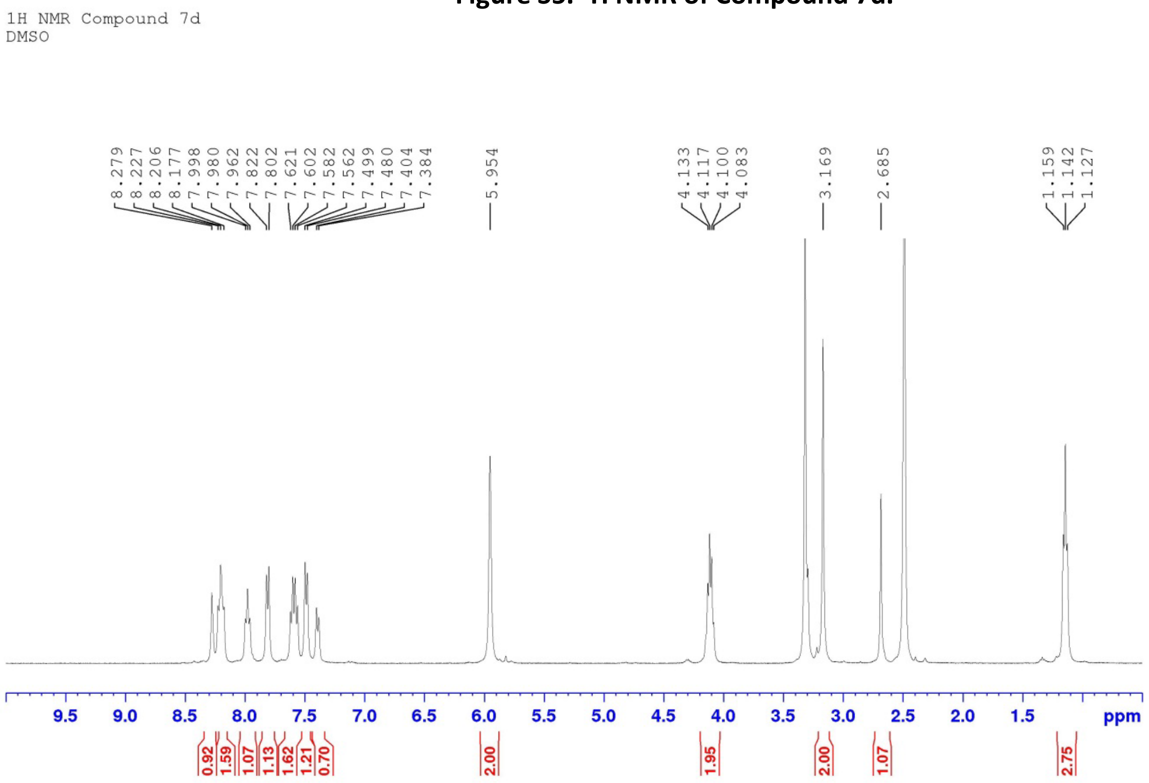

Figure S6. ^{13}C NMR of Compound 7d.

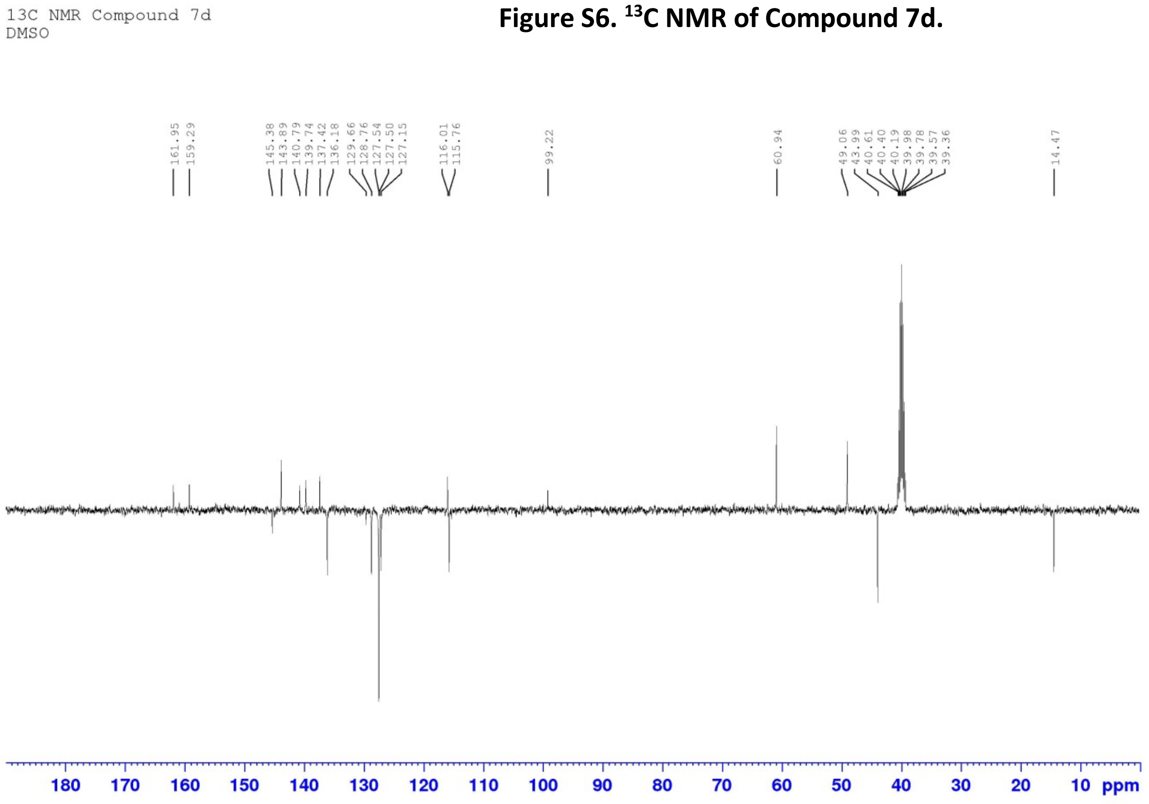

Figure S7. ^{1}H NMR of Compound 7e.

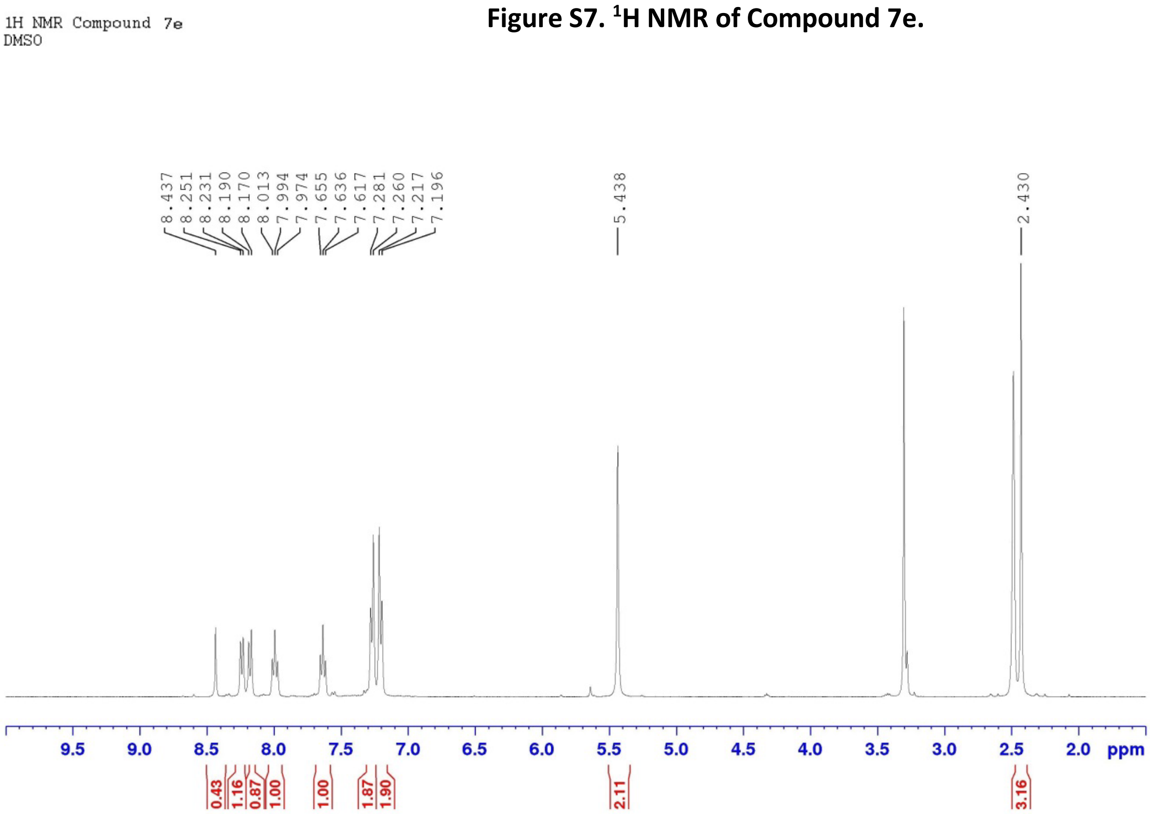

Figure S8. ^{13}C NMR of Compound 7e.

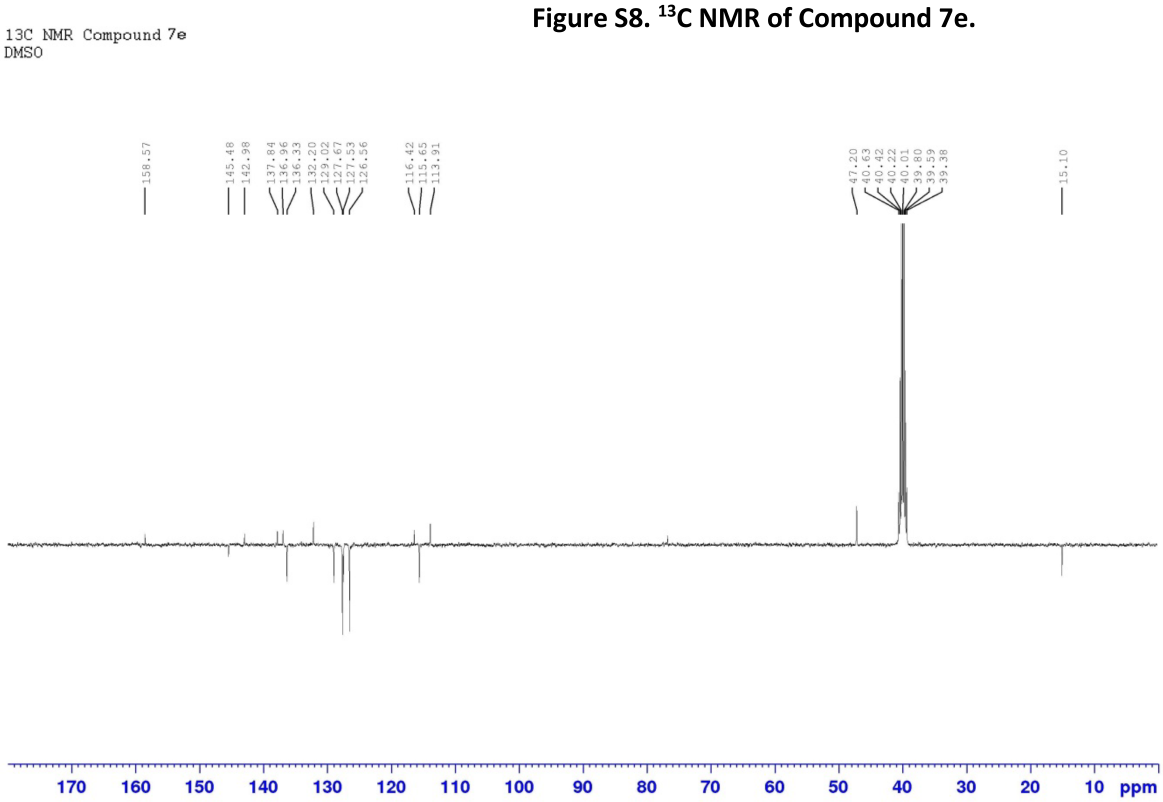

Figure S9. ^{1}H NMR of Compound 7f.

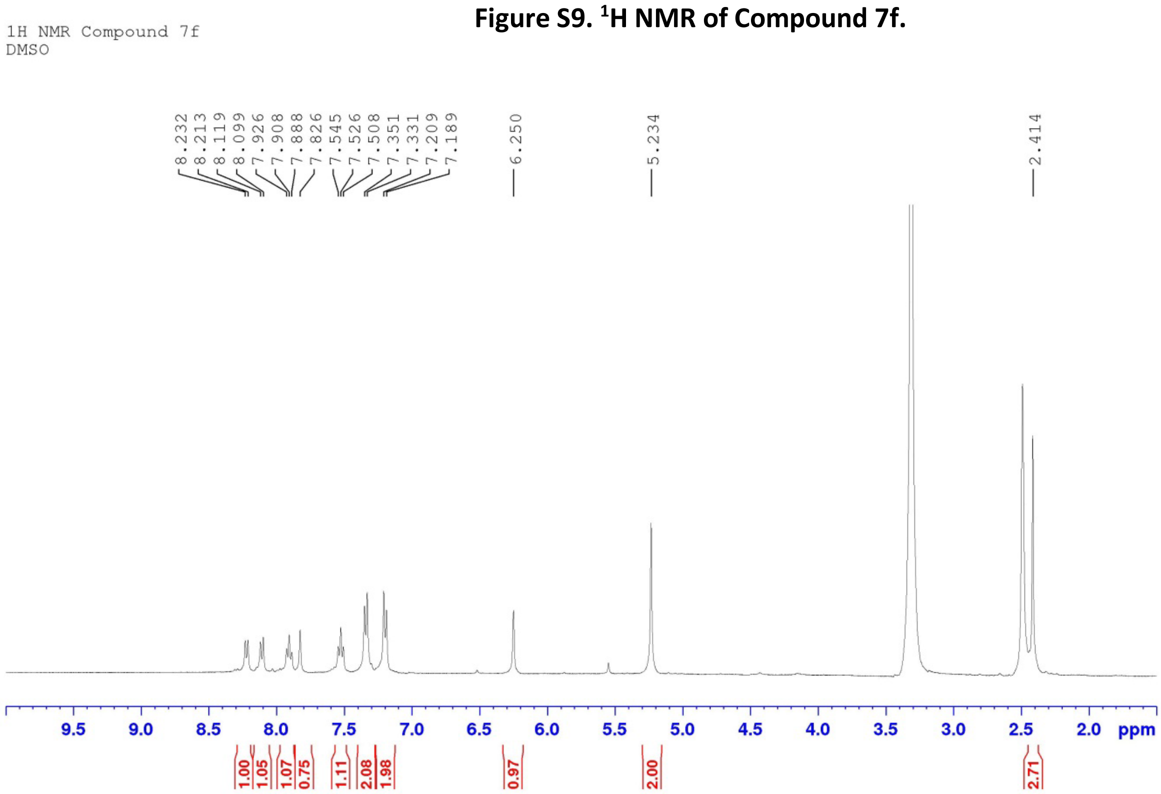

Figure S10. ^{13}C NMR of Compound 7f.

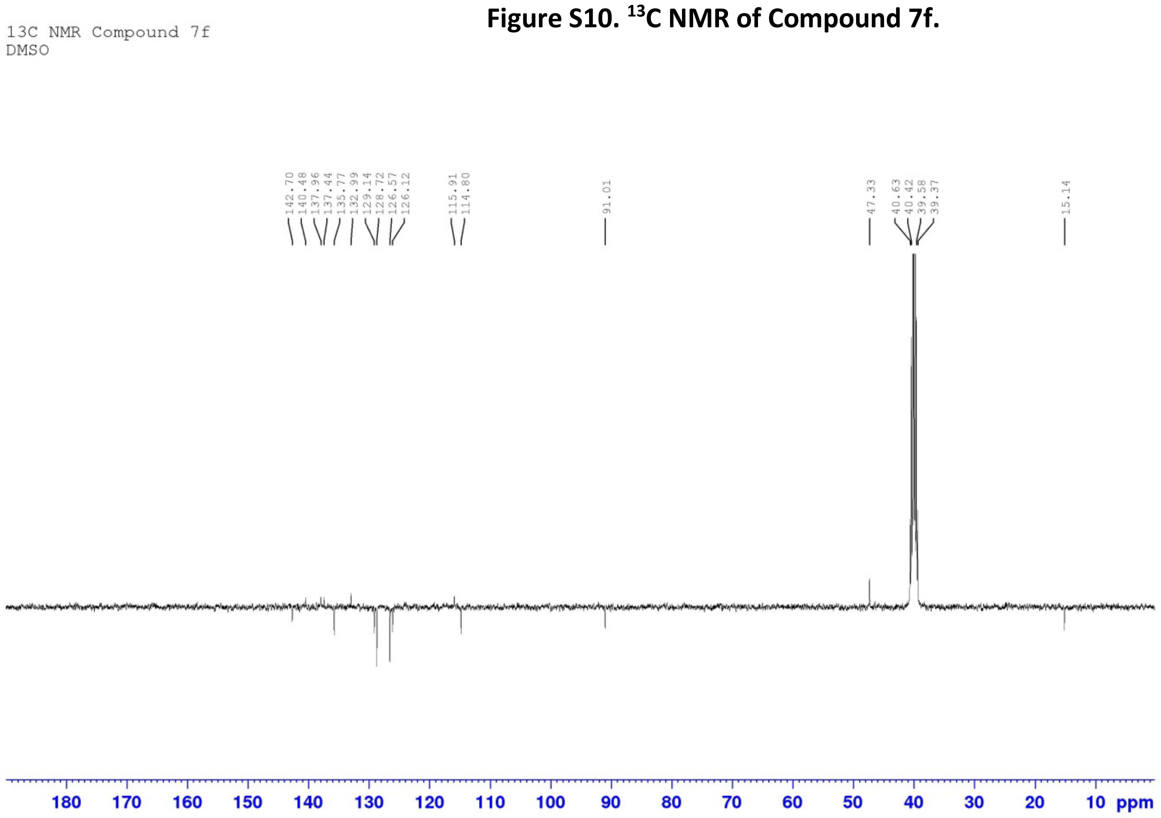

Figure S11. ^{1}H NMR of Compound 8.

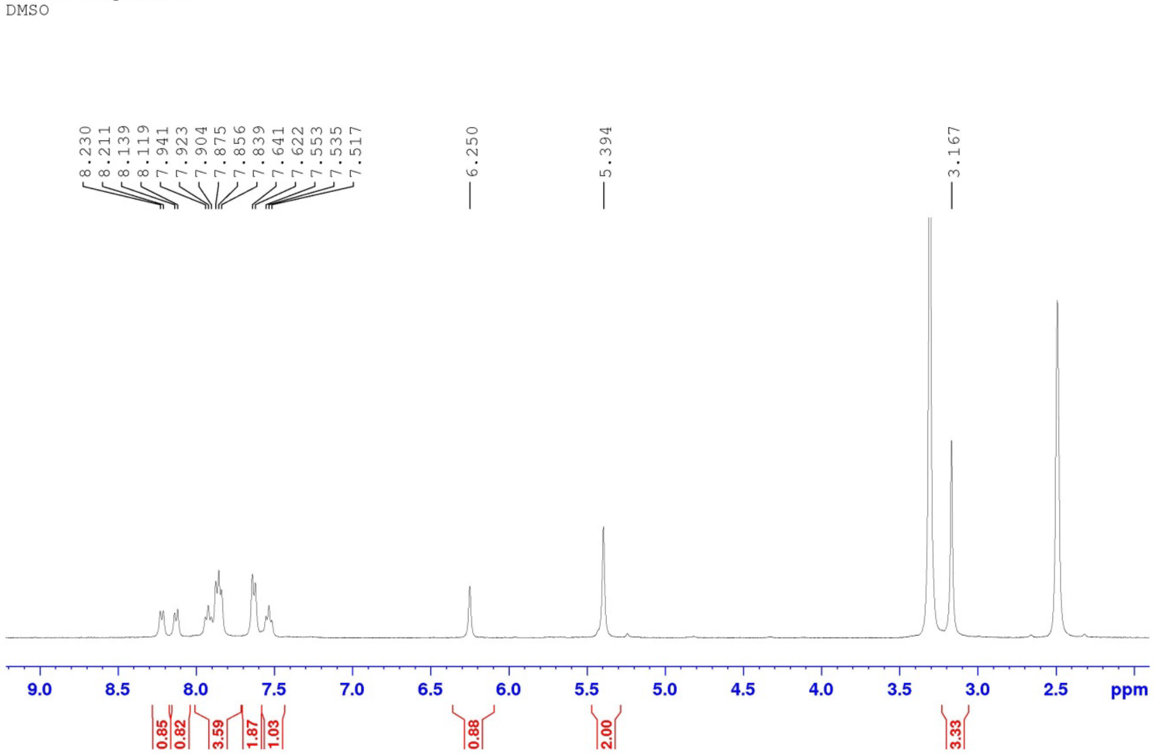

Figure S12. ^{13}C NMR of Compound 8.

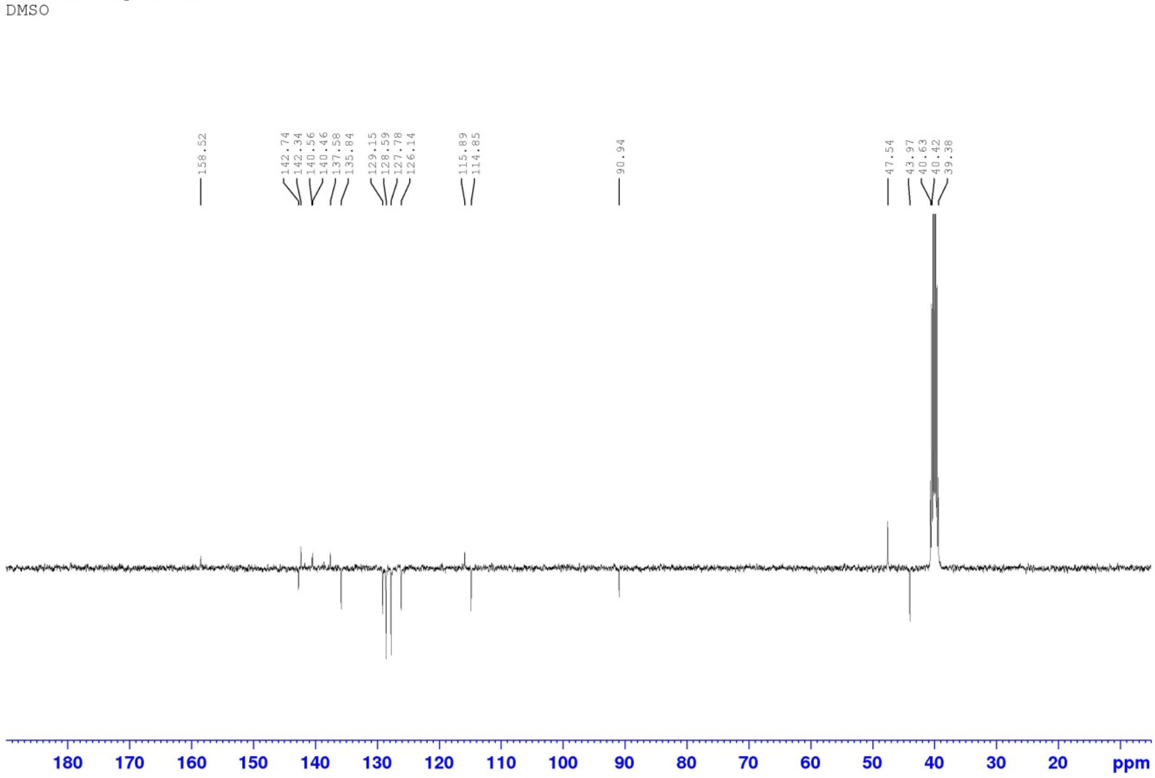

Figure S13. ^{1}H NMR of Compound 9.

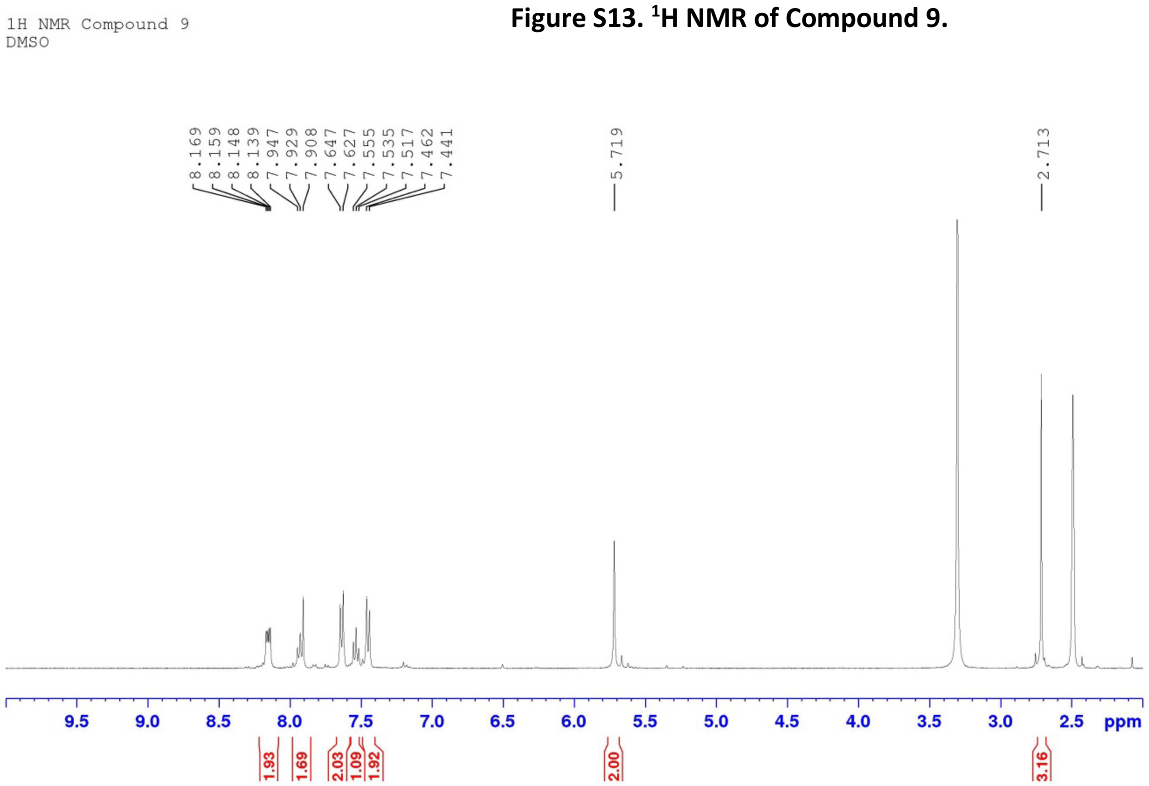

Figure S14. ^{13}C NMR of Compound 9.

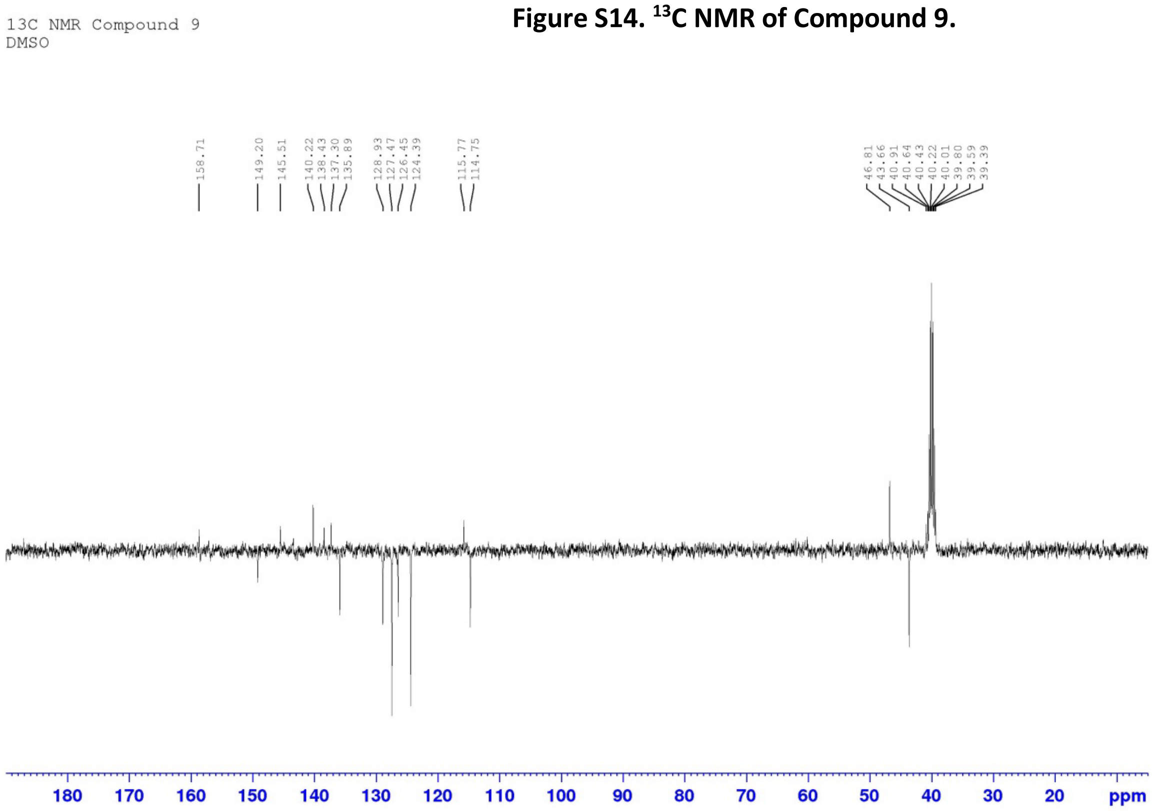

Figure S15. ^{1}H NMR of Compound 10.

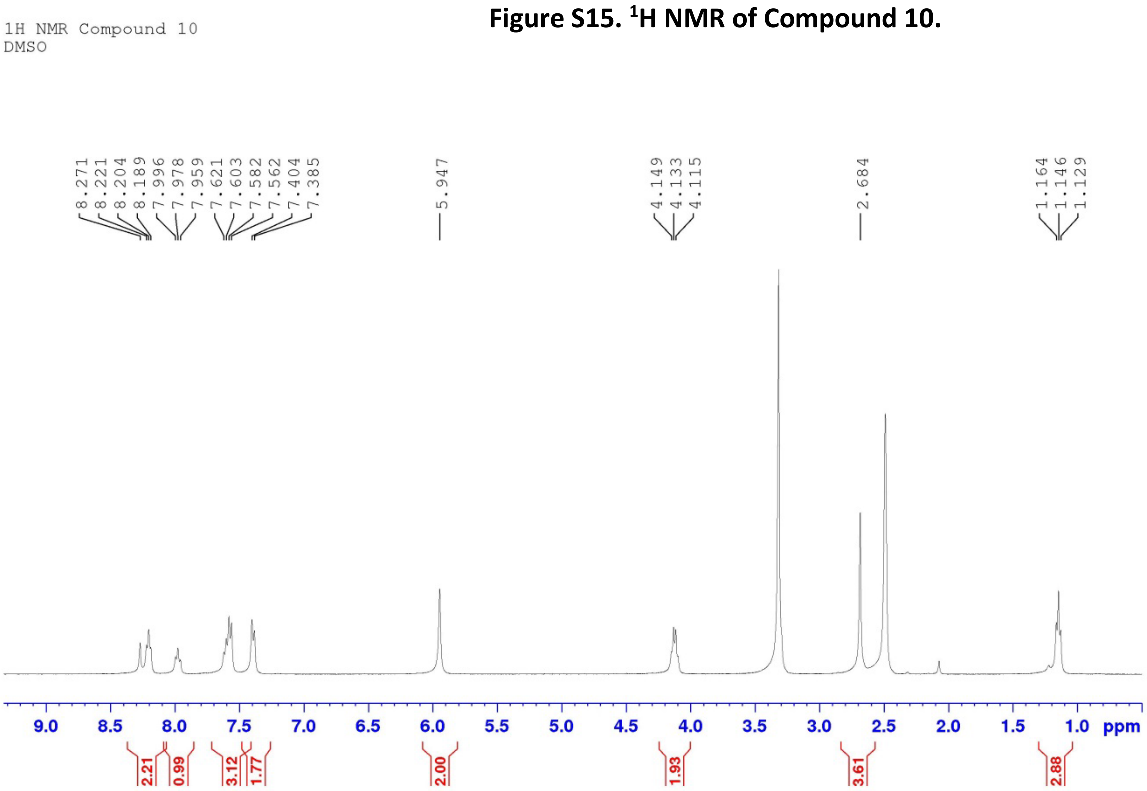

Figure S16. ^{13}C NMR of Compound 10.

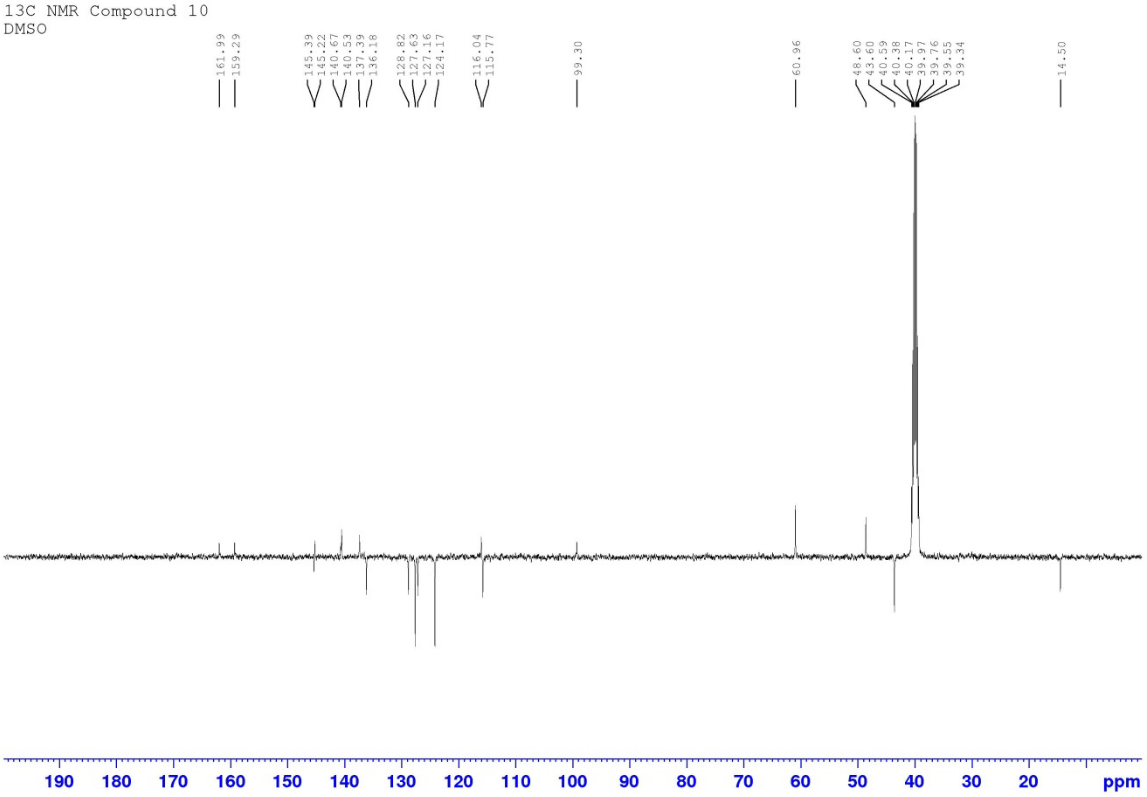

Figure S17. ^{1}H NMR of Compound 13a.

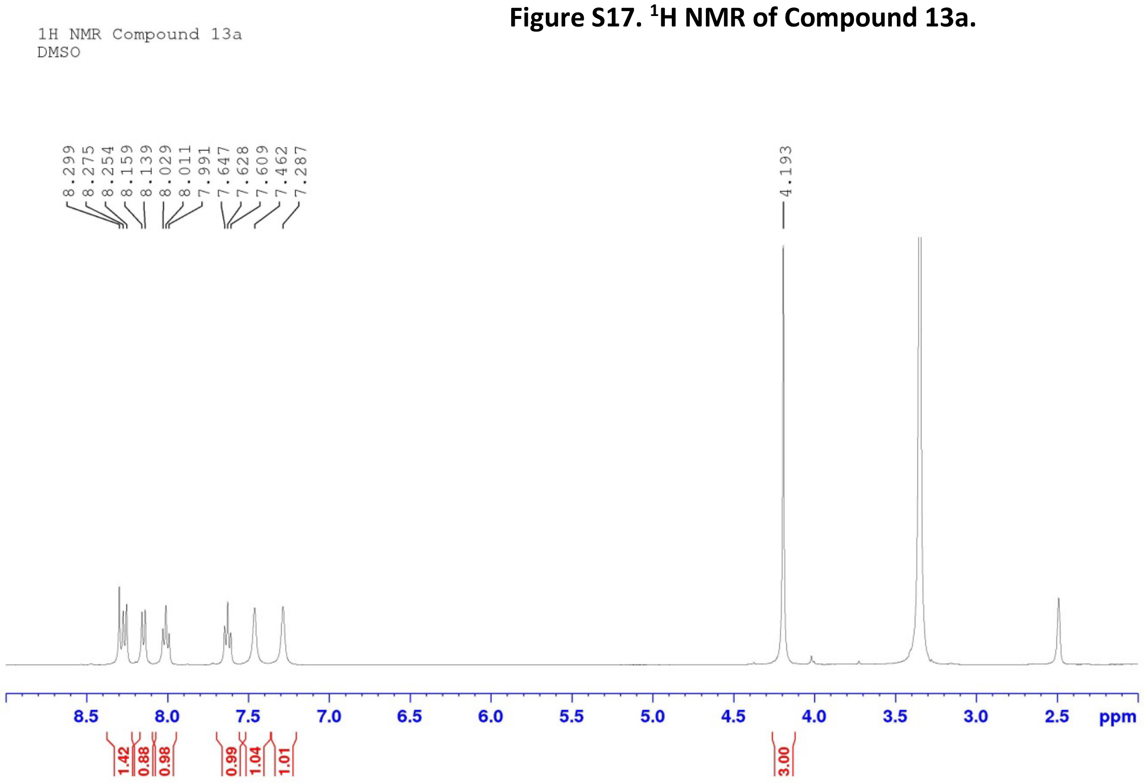

Figure S18. ^{13}C NMR of Compound 13a.

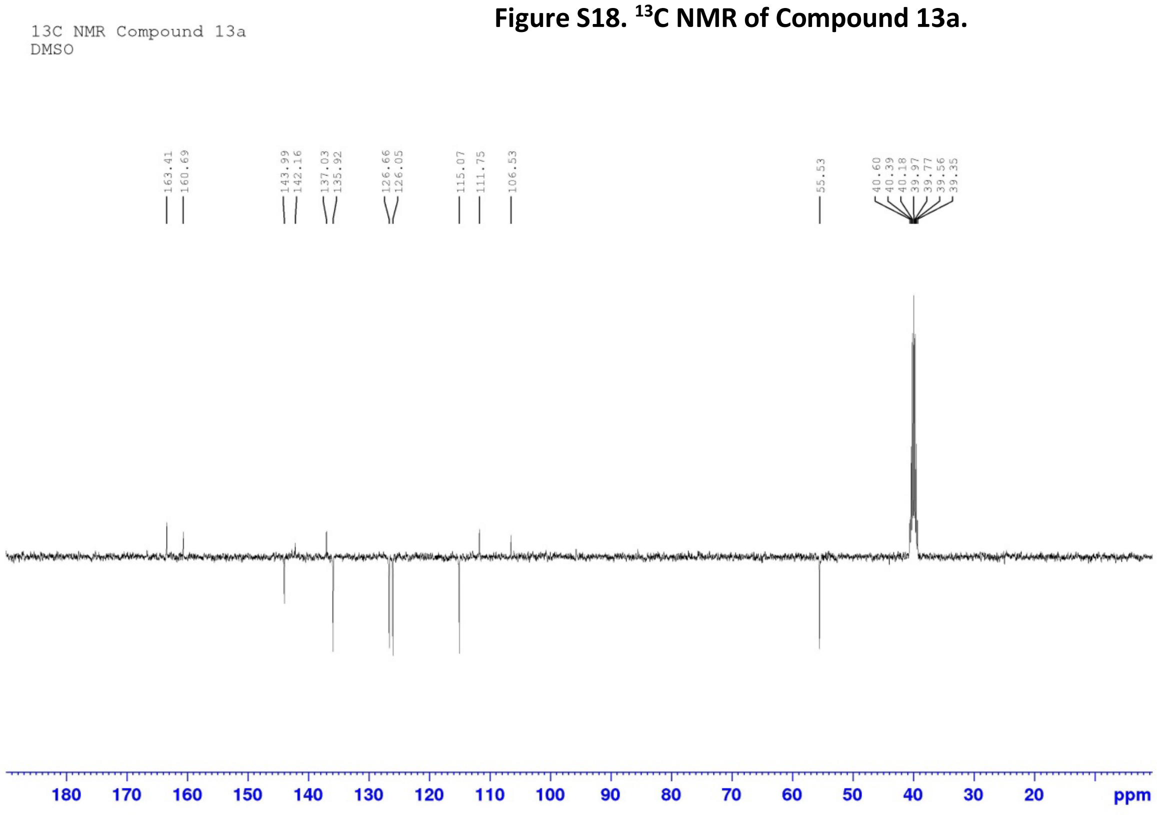

1H NMR Compound 13c
DMSO

Figure S19. ^{1}H NMR of Compound 13c.

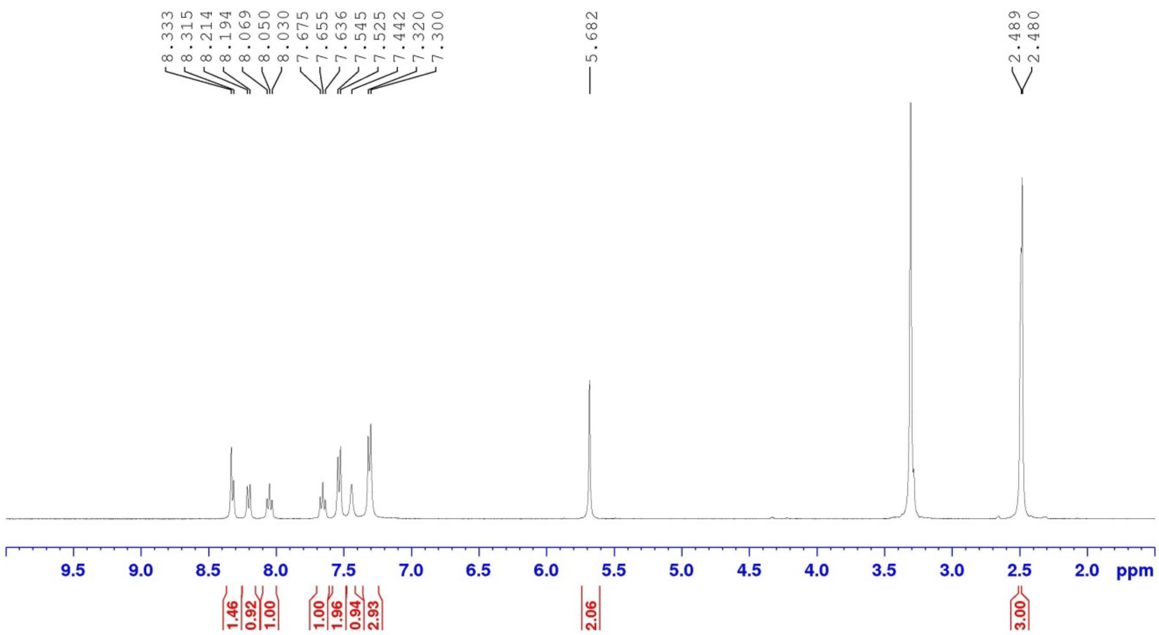

1H NMR Compound 13c
DMSO + D2O

Figure S20. ^{1}H NMR of Compound 13c + D_2O

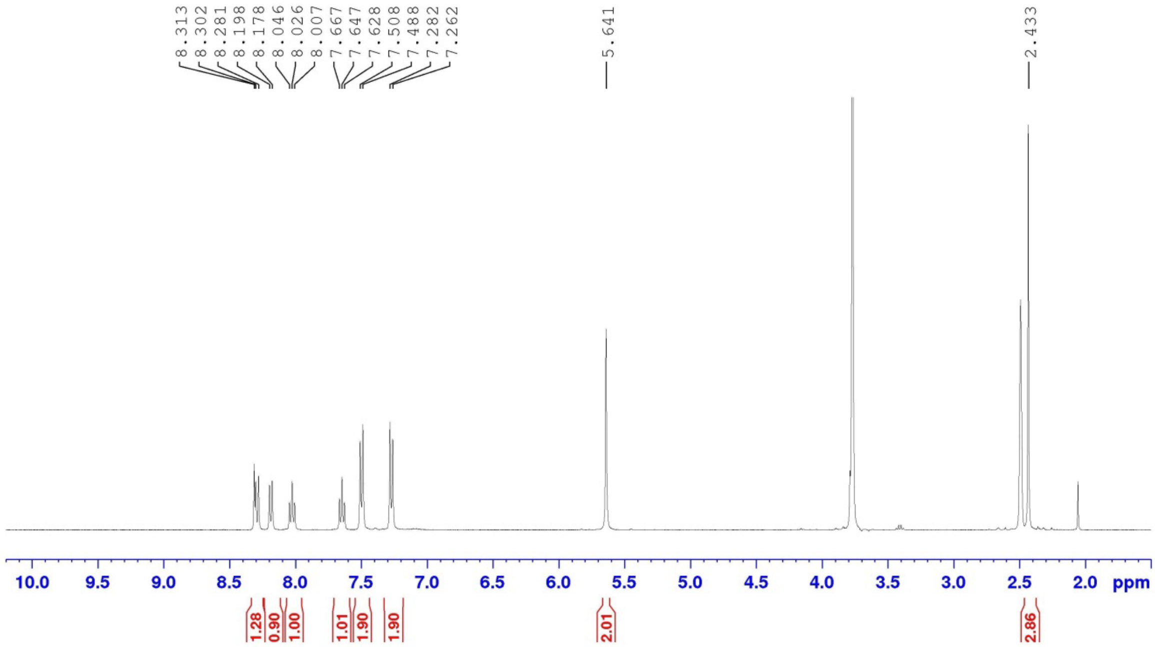

Figure S21. ^{13}C NMR of Compound 13c.

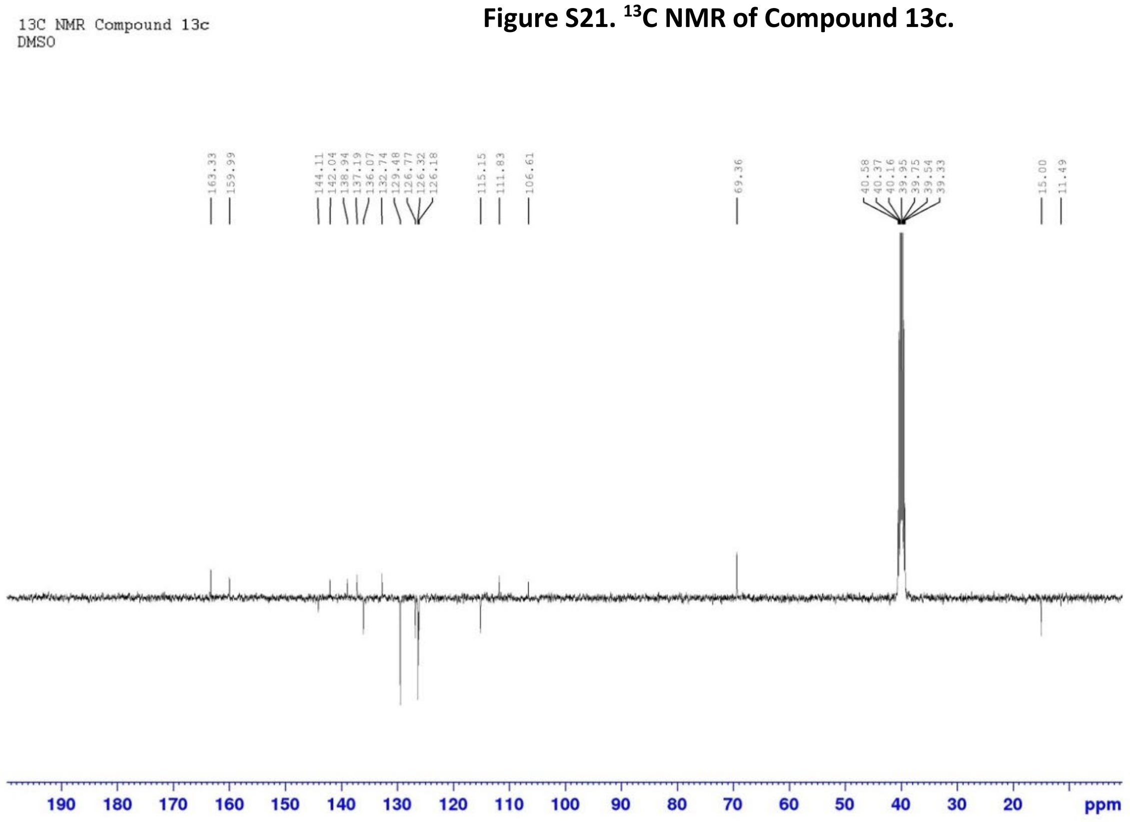

Figure S22. ^{1}H NMR of Compound 13f.

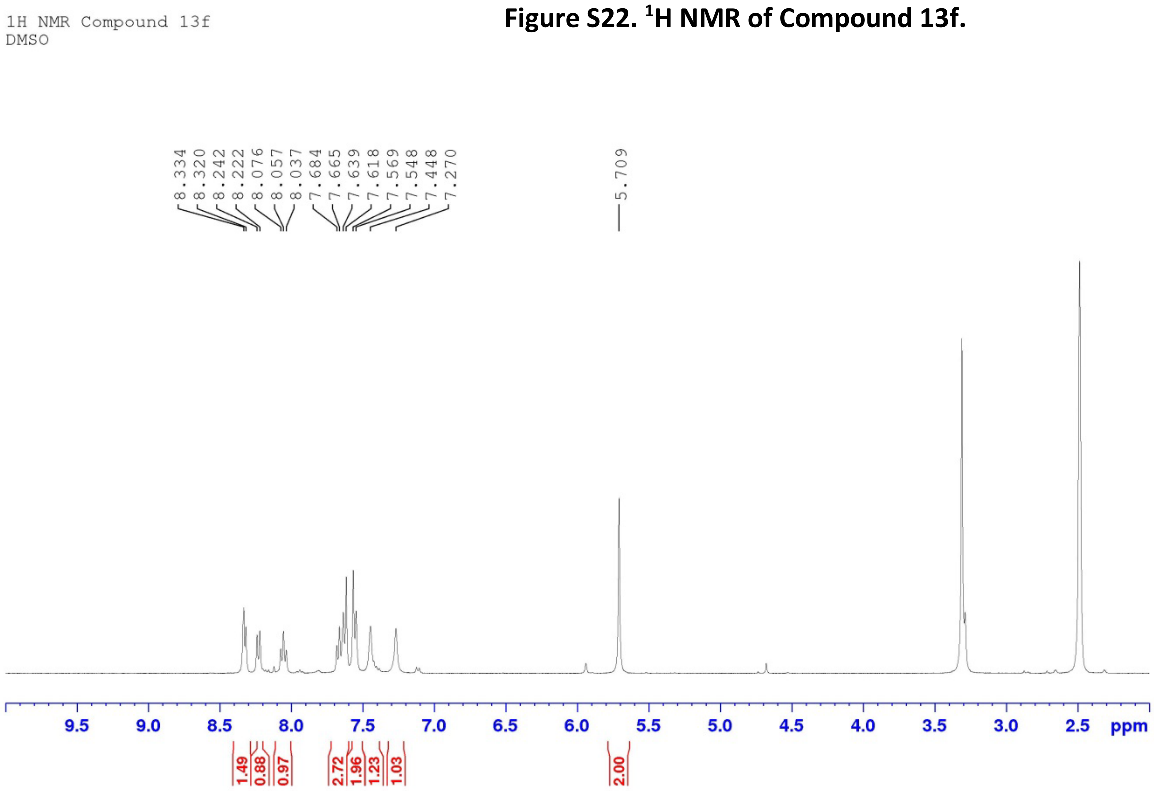

Figure S23. ^{13}C NMR of Compound 13f.

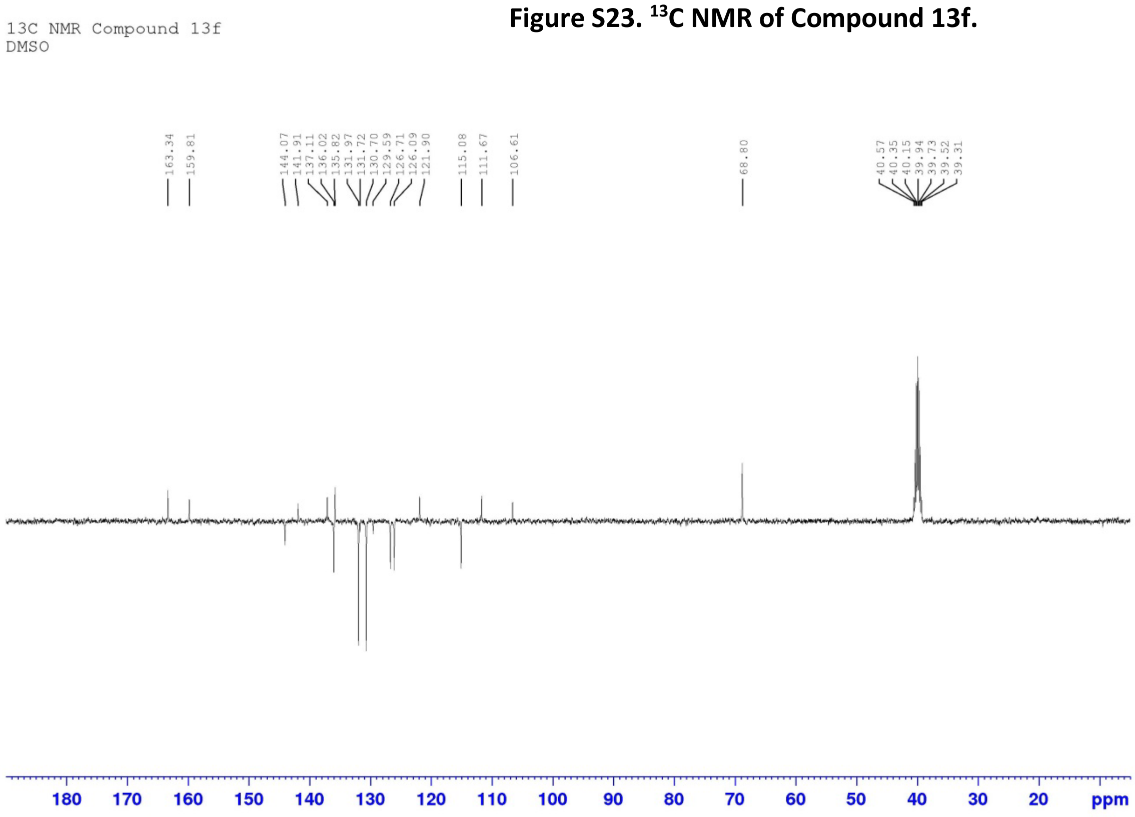

Figure S24. ^{1}H NMR of Compound 13h.

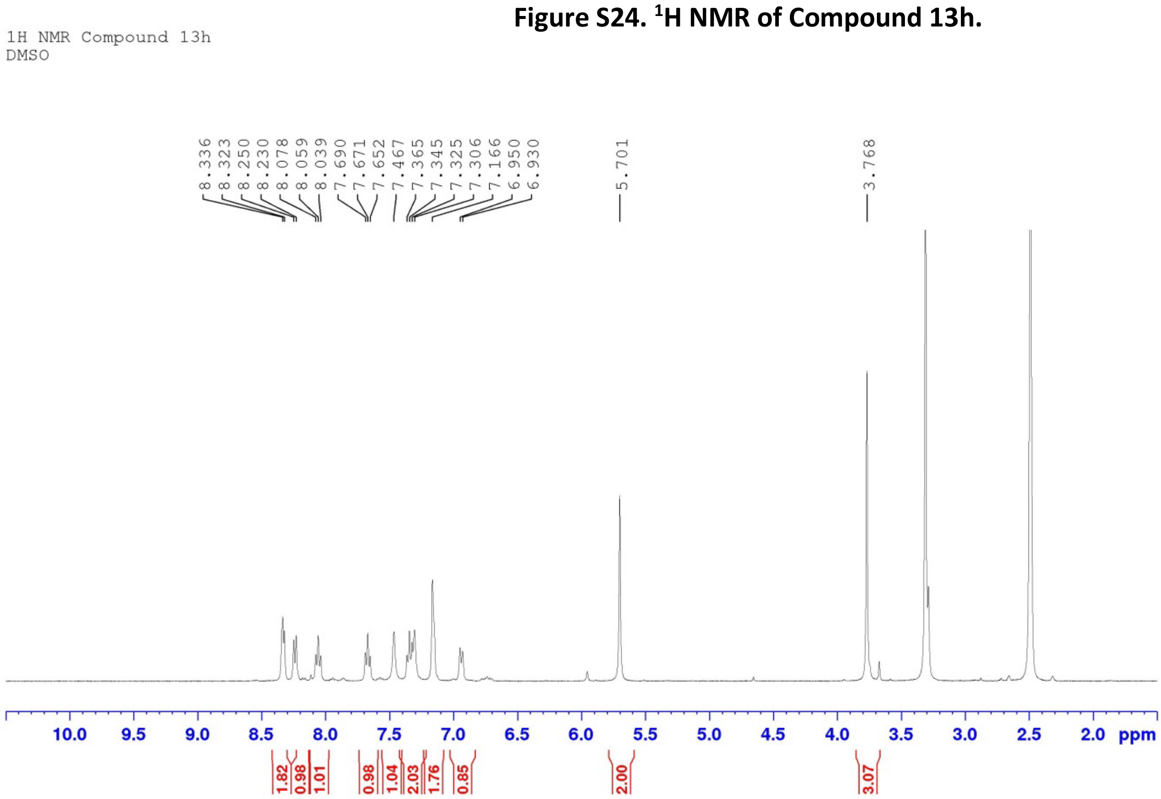

Figure S25. ^{13}C NMR of Compound 13h.

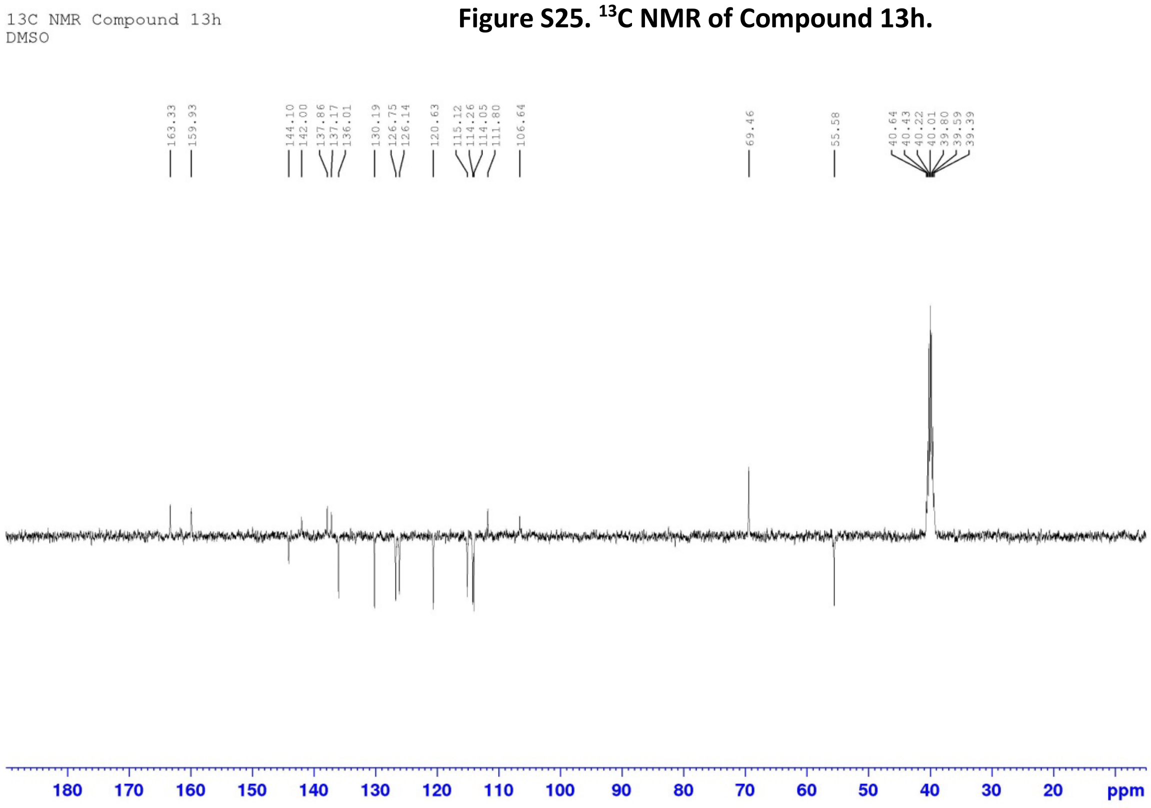

Figure S26. ^{1}H NMR of Compound 13i.

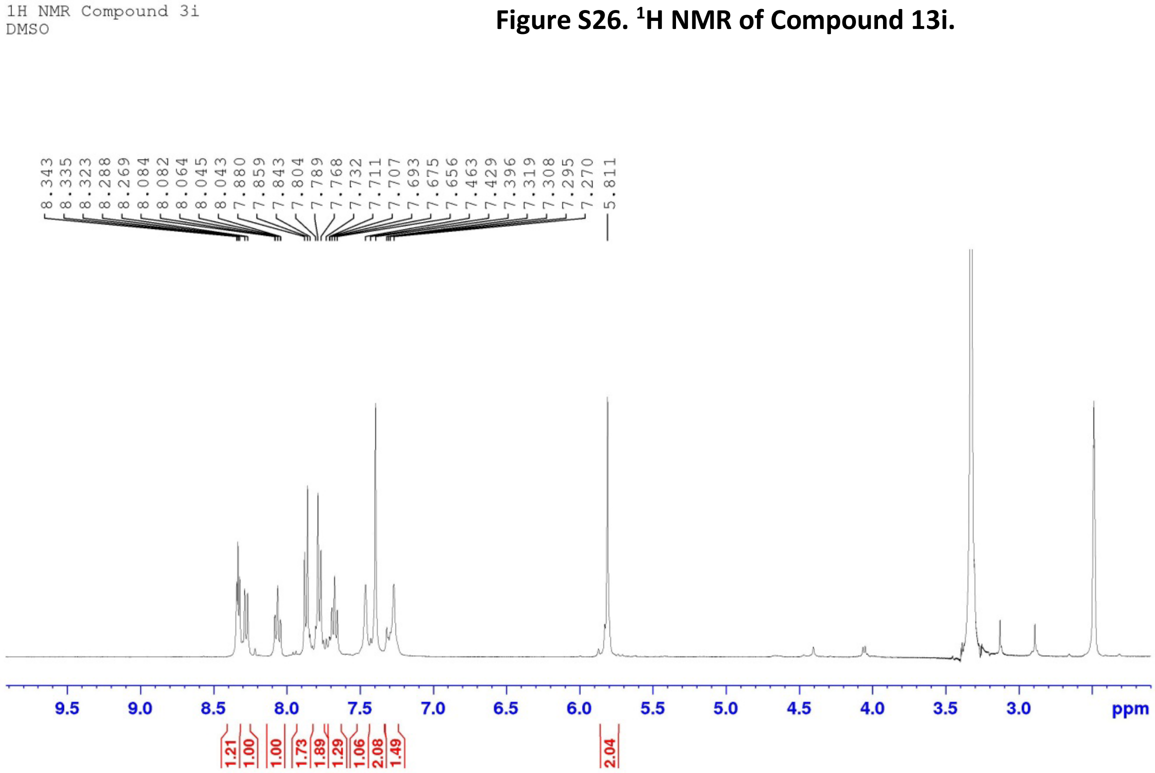

Figure S27. ^{1}H NMR of Compound 13i + D_2O.

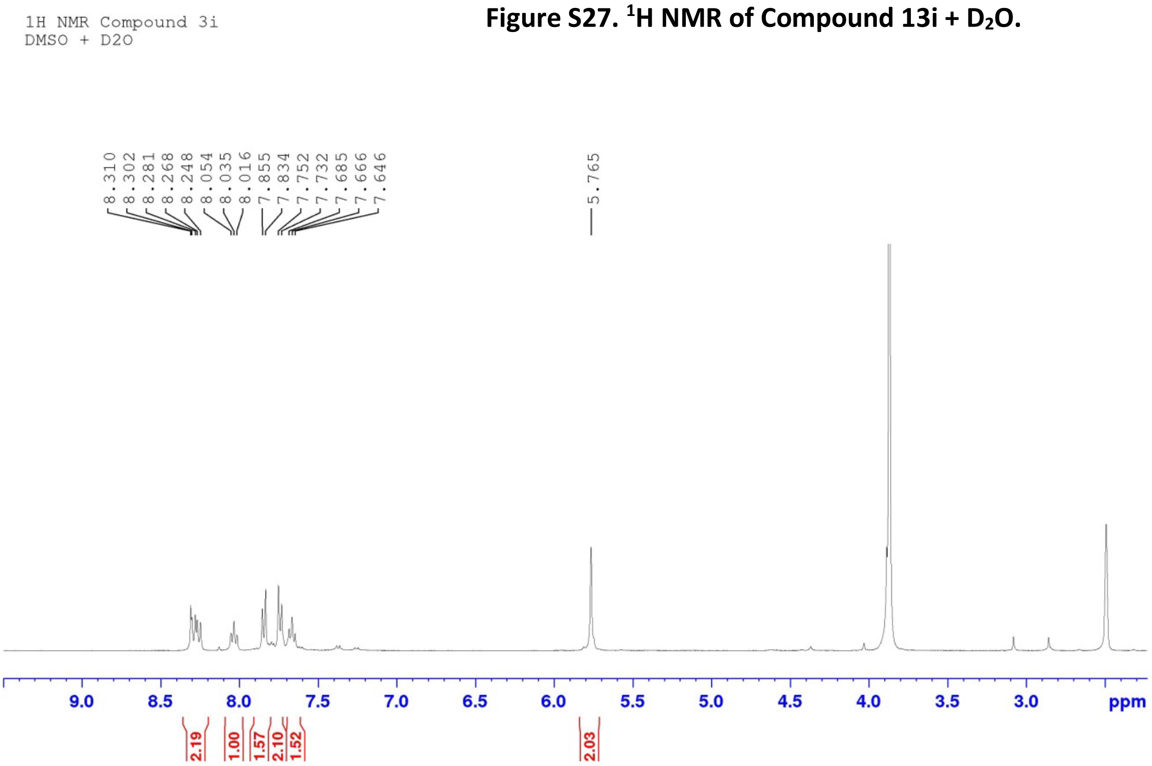

Figure S28. ^{13}C NMR of Compound 13i.

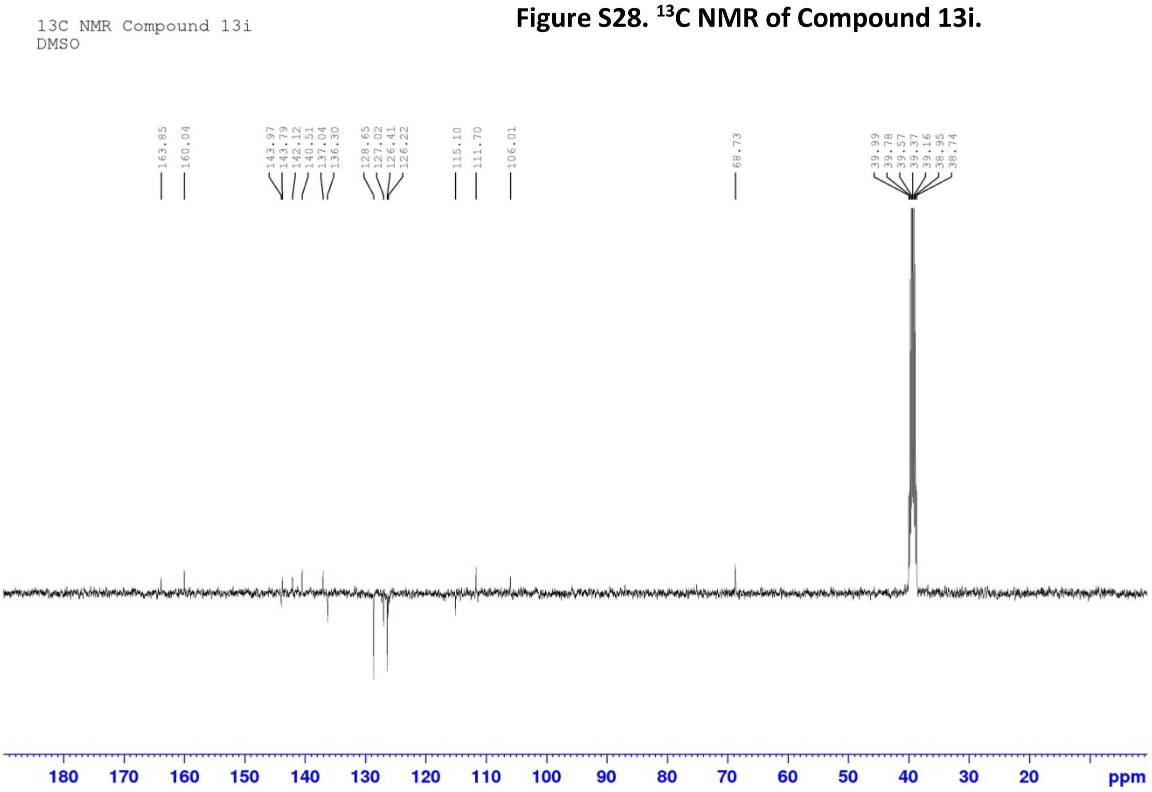

Figure S29. ^{1}H NMR of Compound 14c.

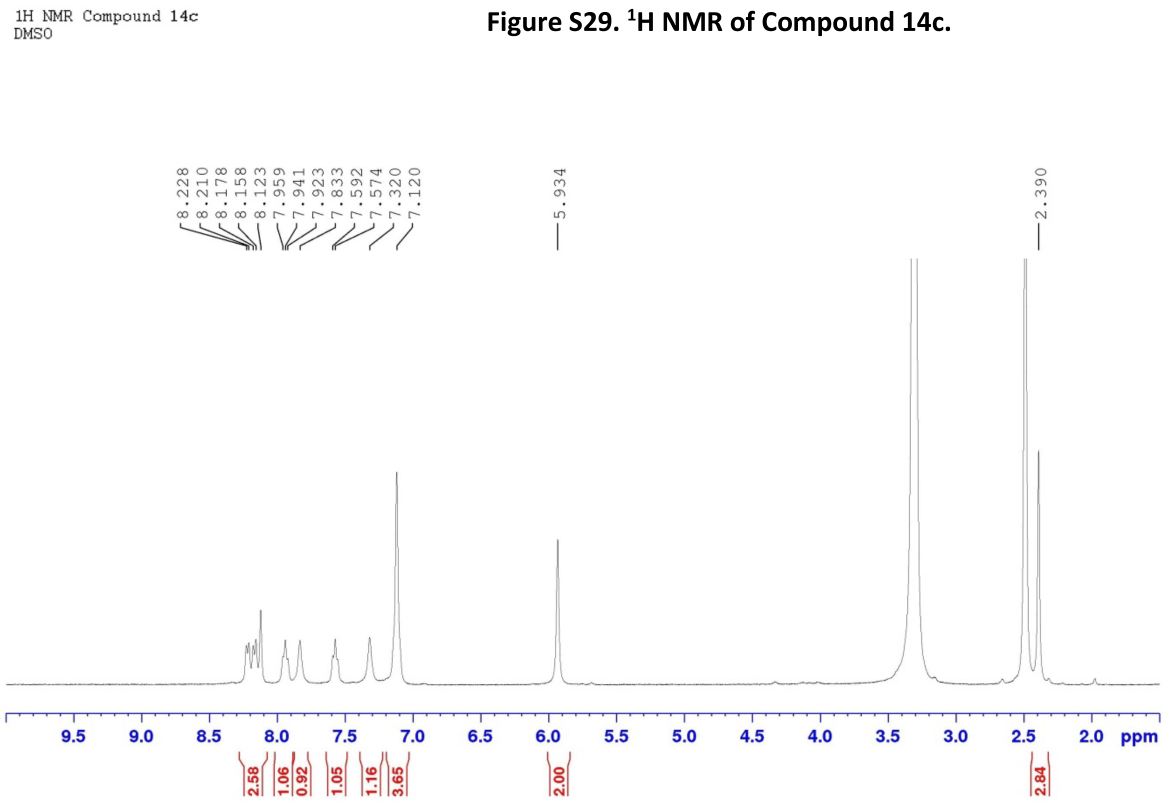

Figure S30. ^{13}C NMR of Compound 14c.

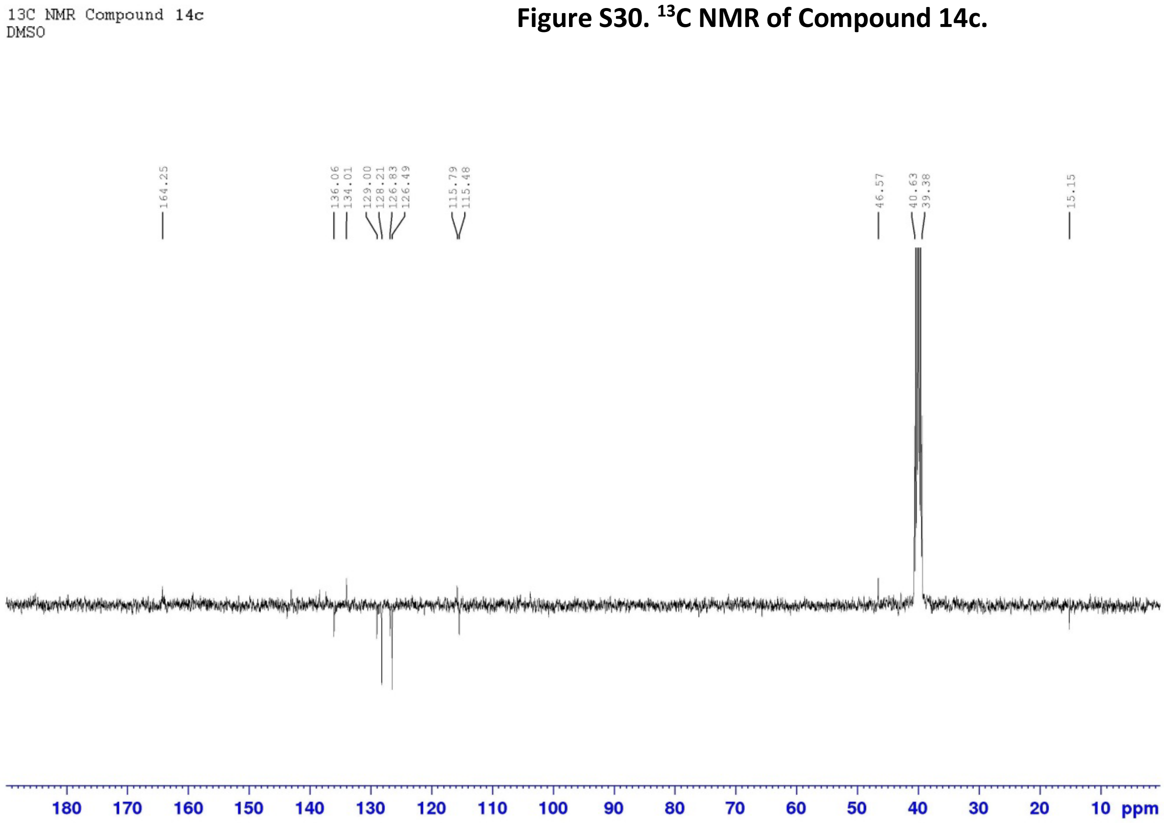

Figure S31. ^{1}H NMR of Compound 16.

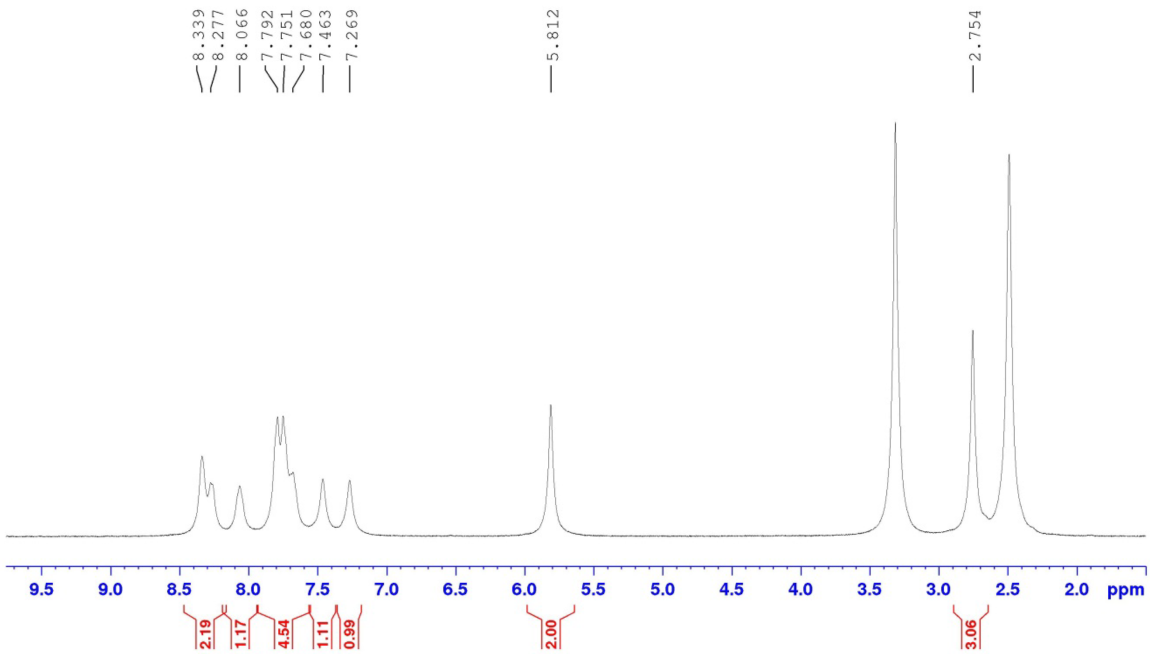

Figure S32. ^{13}C NMR of Compound 16.

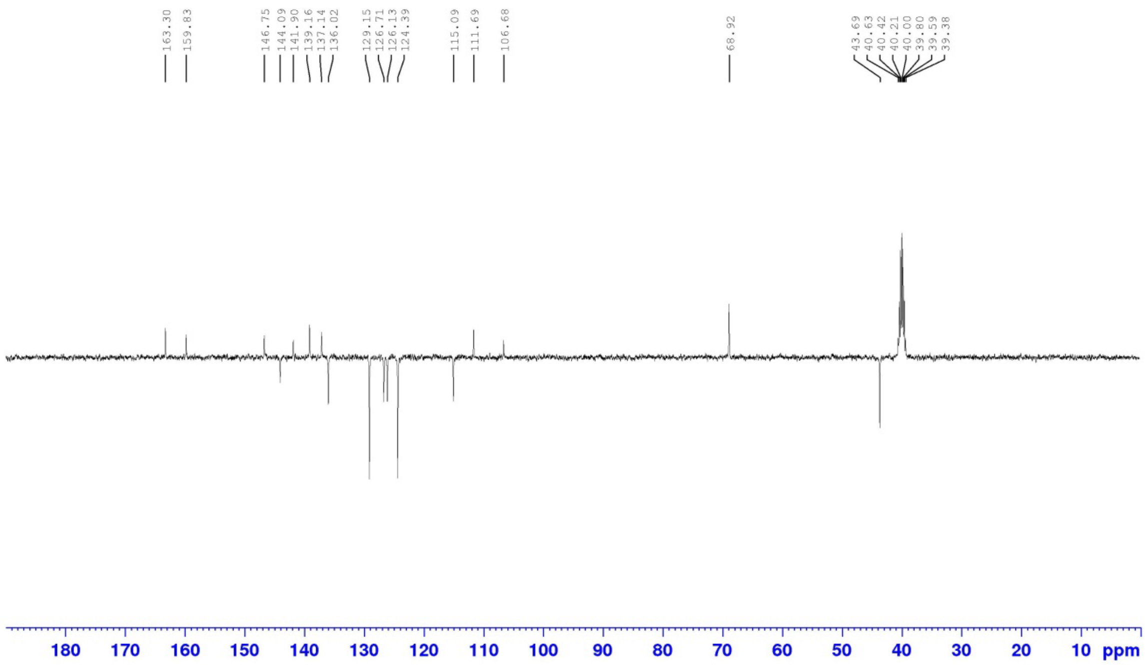

Figure S33. ^{1}H NMR of Compound 23b.

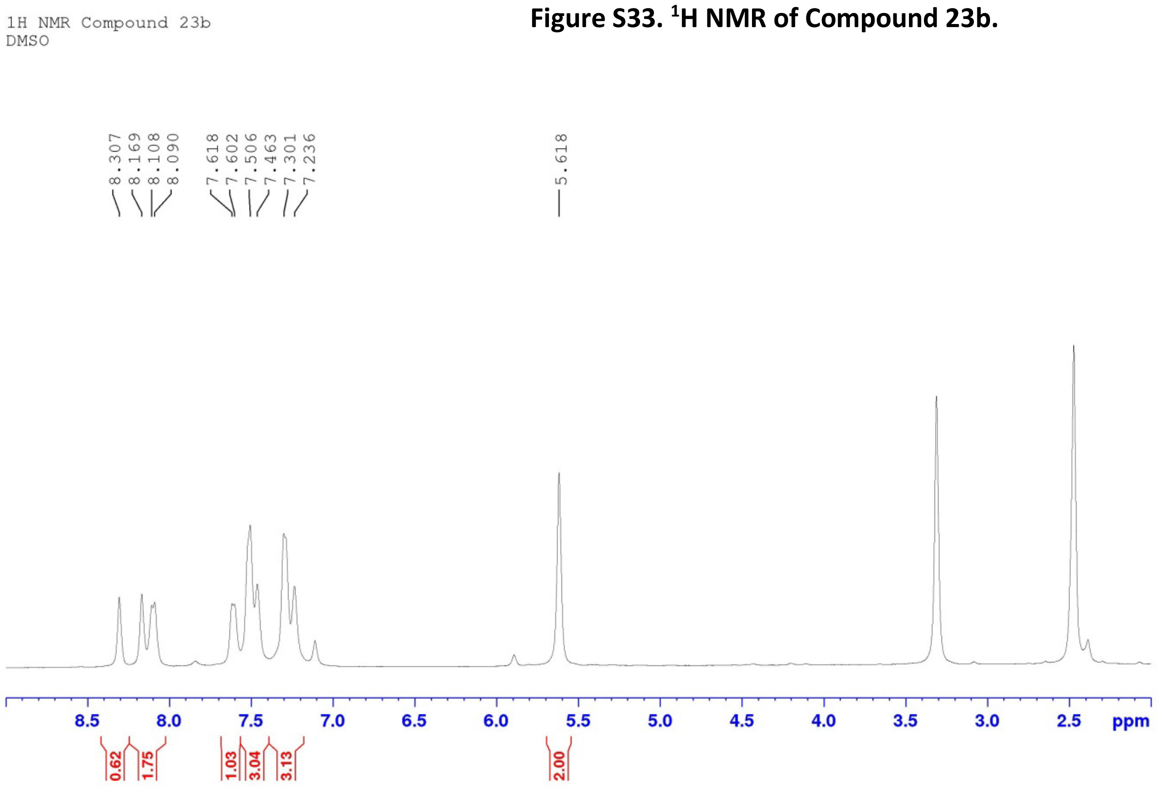

Figure S34. ^{13}C NMR of Compound 23b.

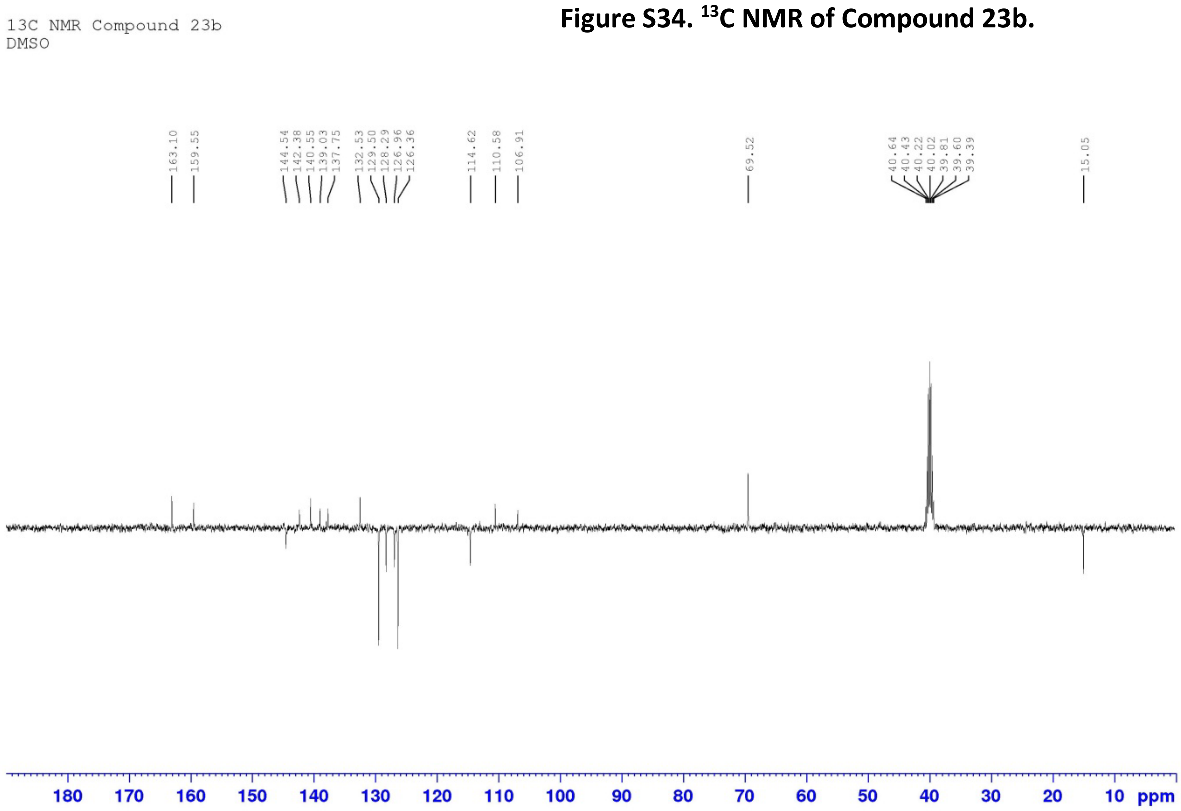

Figure S35. ^{1}H NMR of Compound 29.

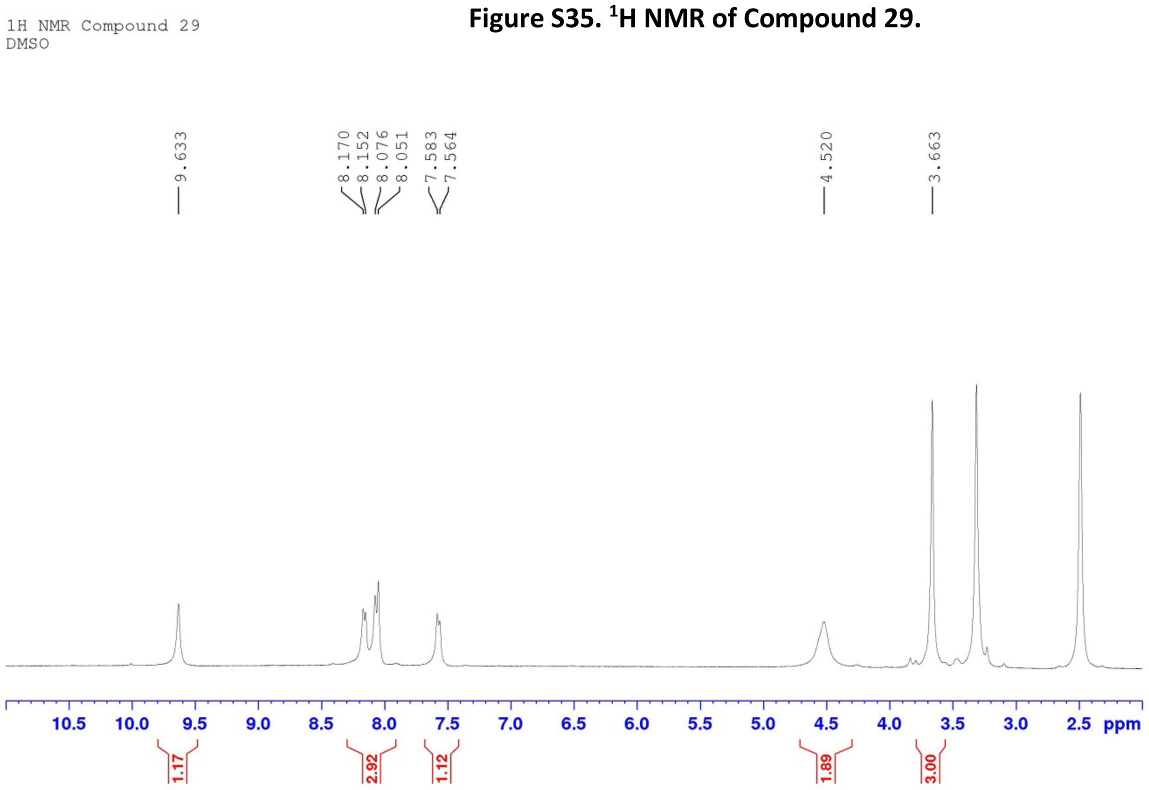

Figure S36. ^{13}C NMR of Compound 29.

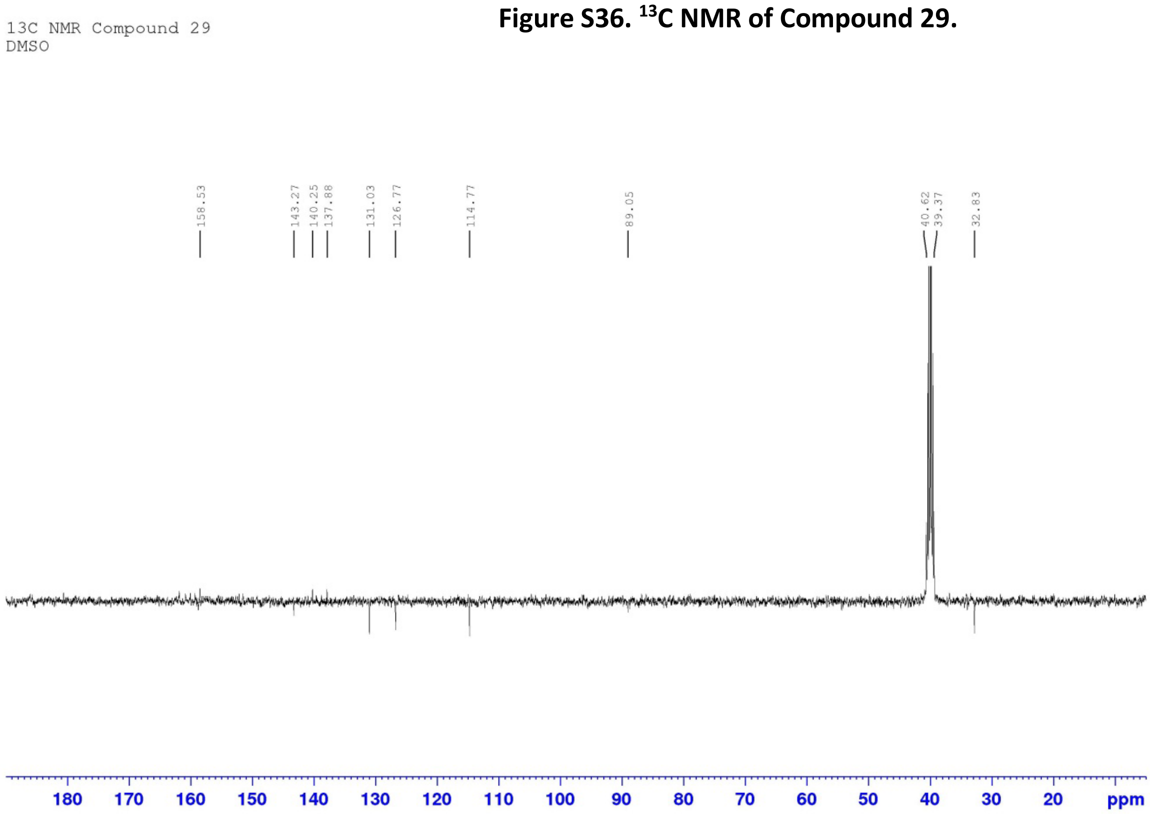

Figure S37. ^{1}H NMR of Compound 31.

1H NMR Compound 31
CDCl3

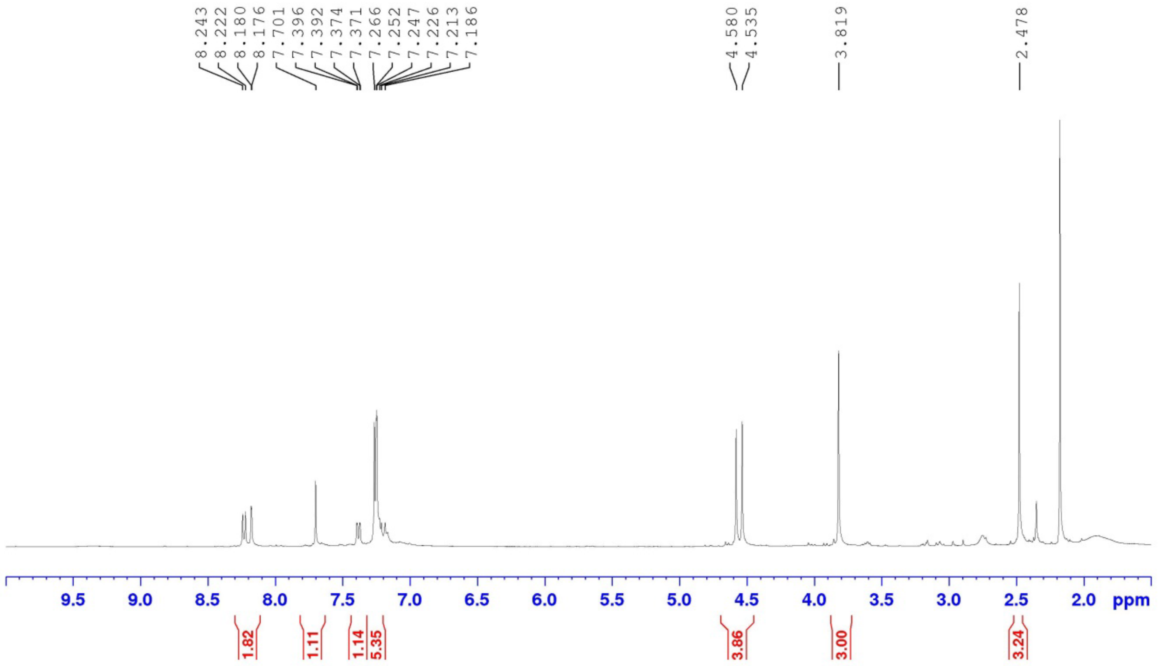

Figure S38. ^{13}C NMR of Compound 31.

13C NMR Compound 31
CDCl3

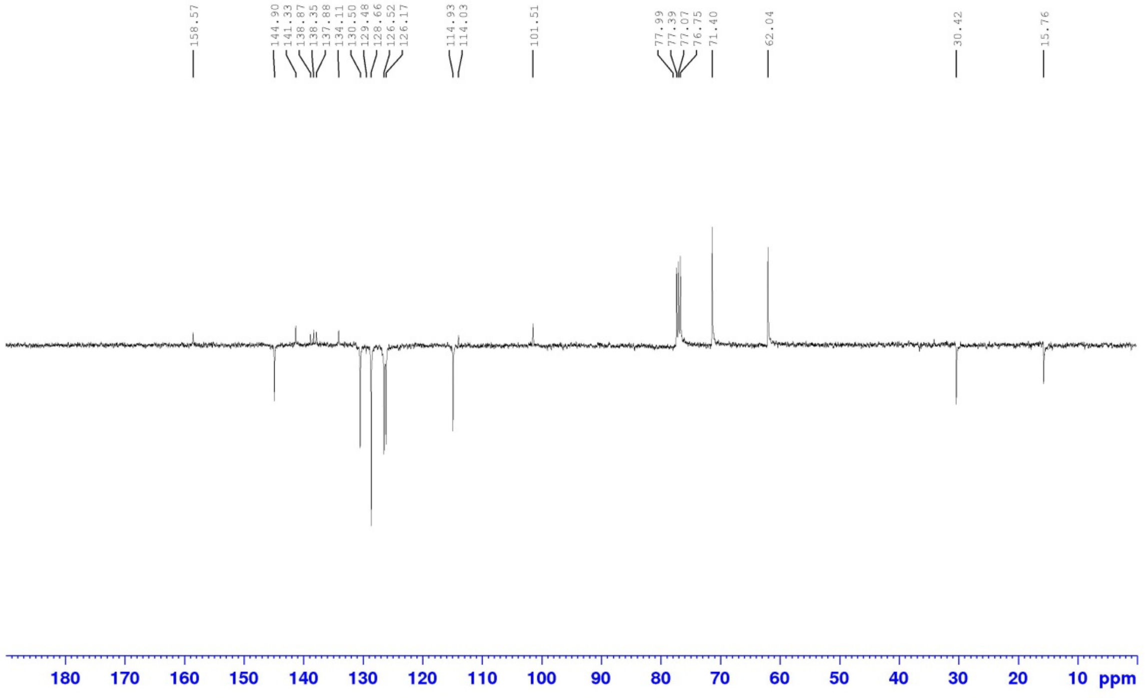

Figure S39. ^{1}H NMR of Compound 33a.

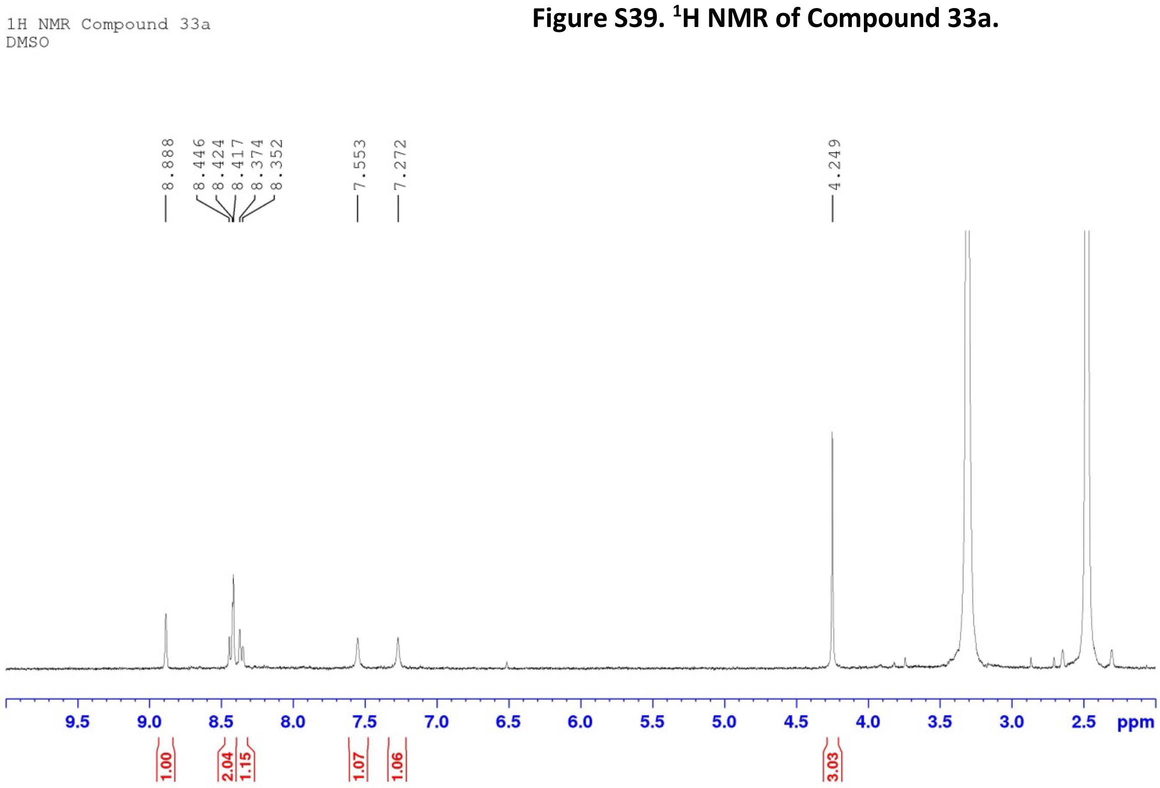

Figure S40. ^{13}C NMR of Compound 33a.

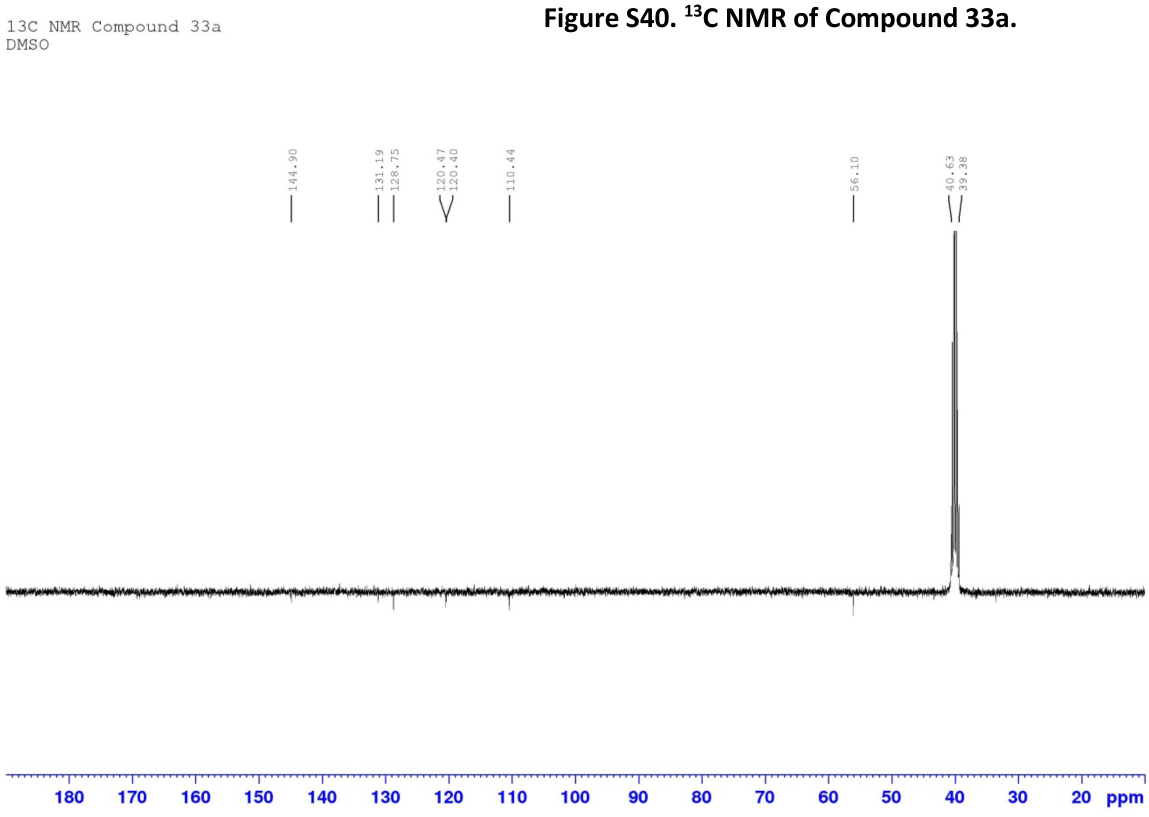

Figure S41. ^{1}H NMR of Compound 33b.

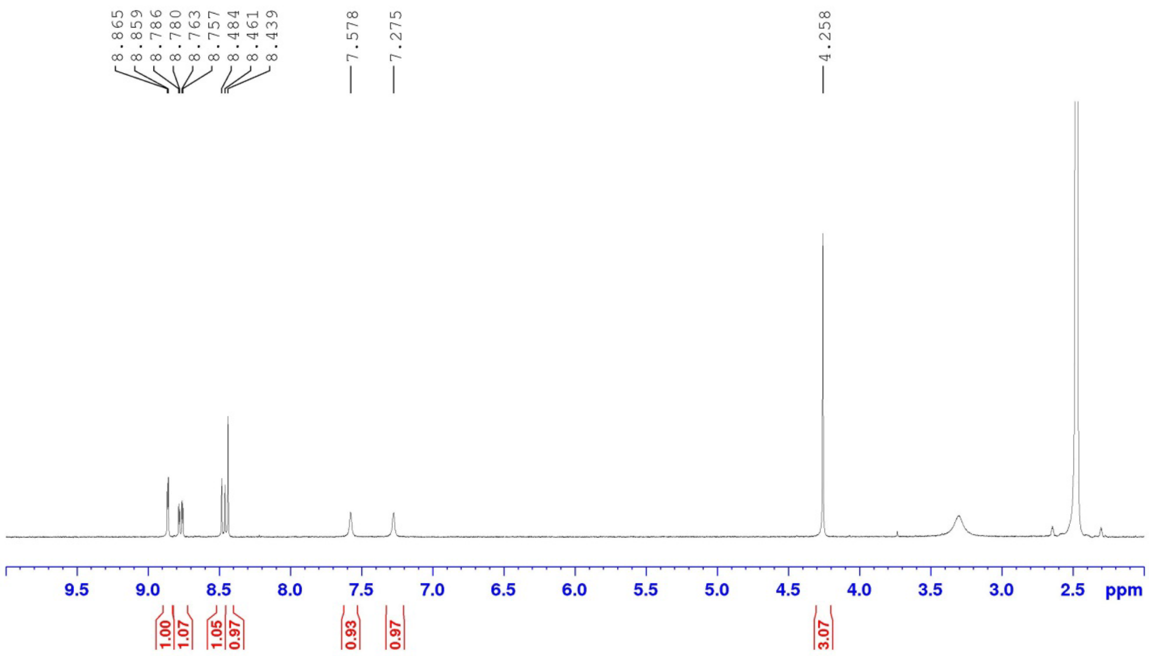

Figure S42. ^{1}H NMR of Compound 33b + D_2O.

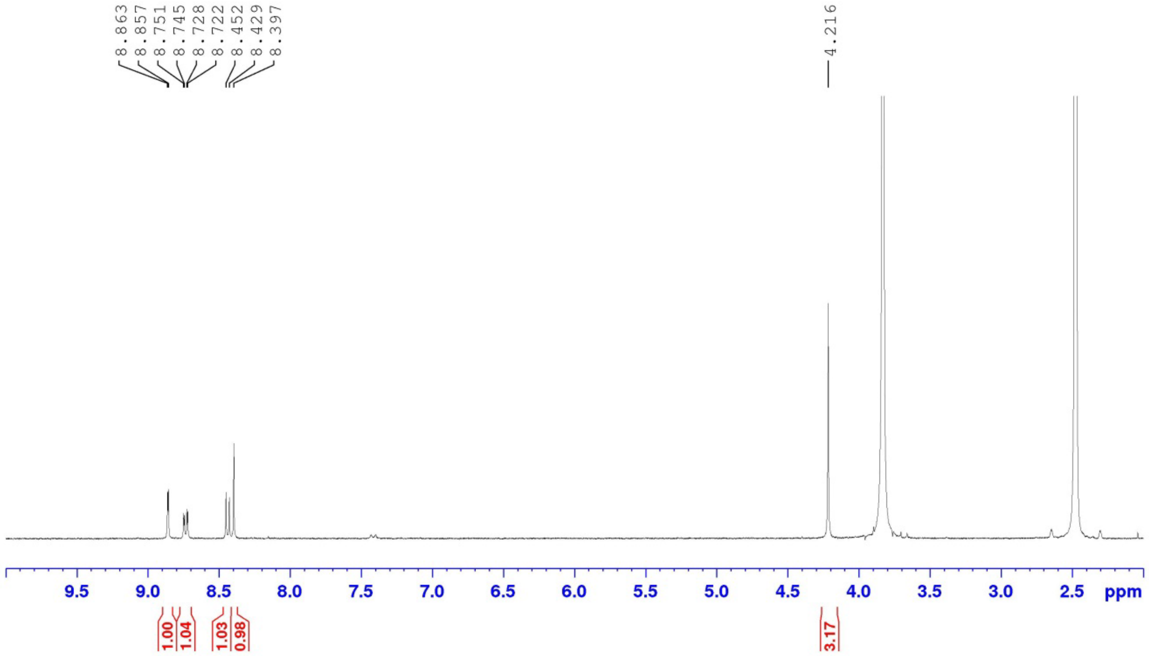

Figure S43. ^{13}C NMR of Compound 33b.

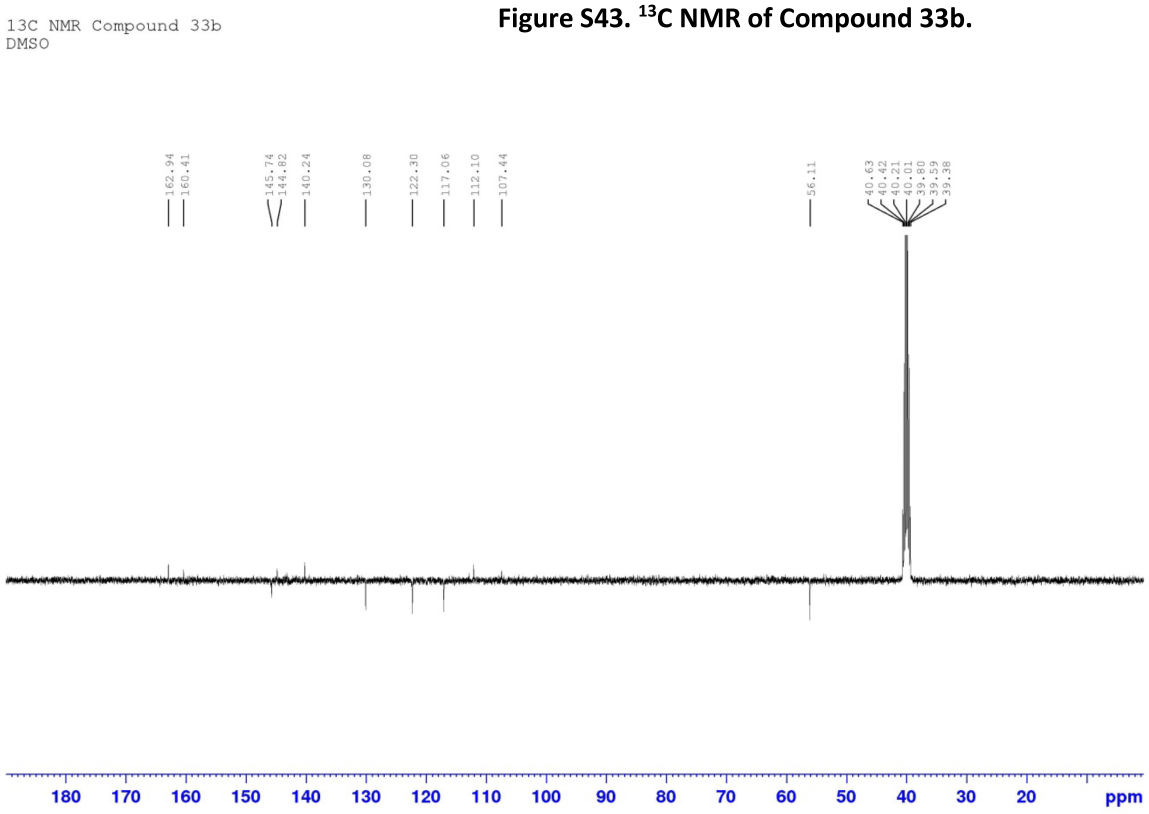

Figure S44. ^{1}H NMR of Compound 34.

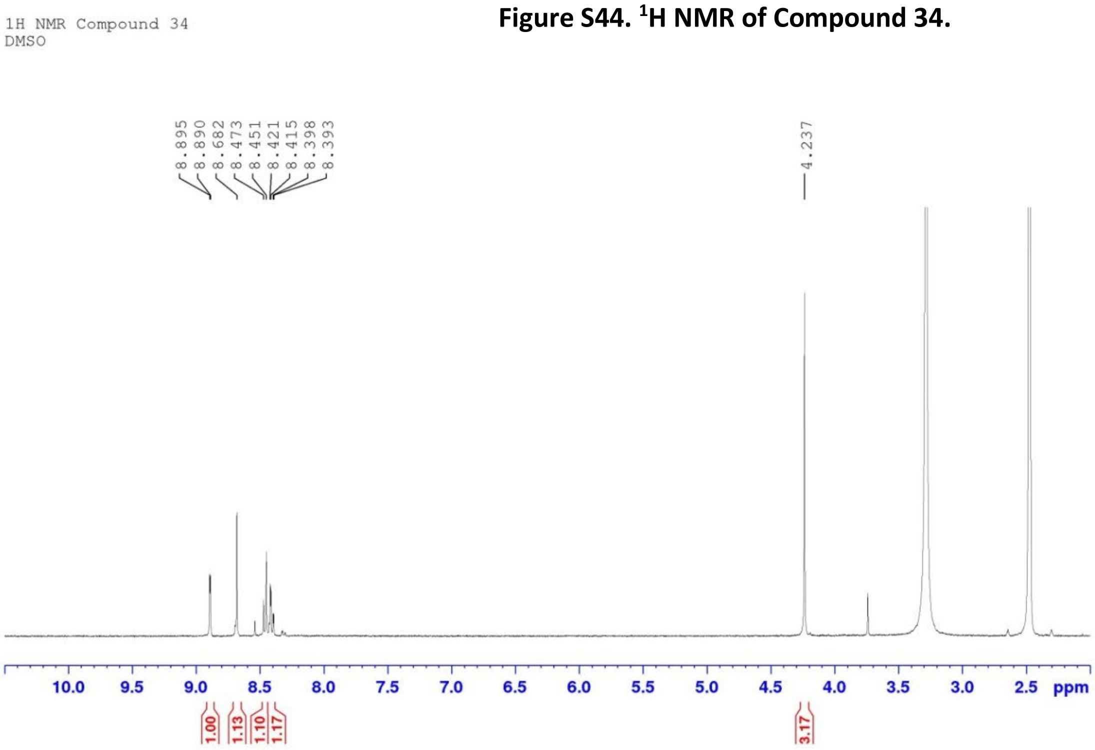

Figure S45. ^{13}C NMR of Compound 34.

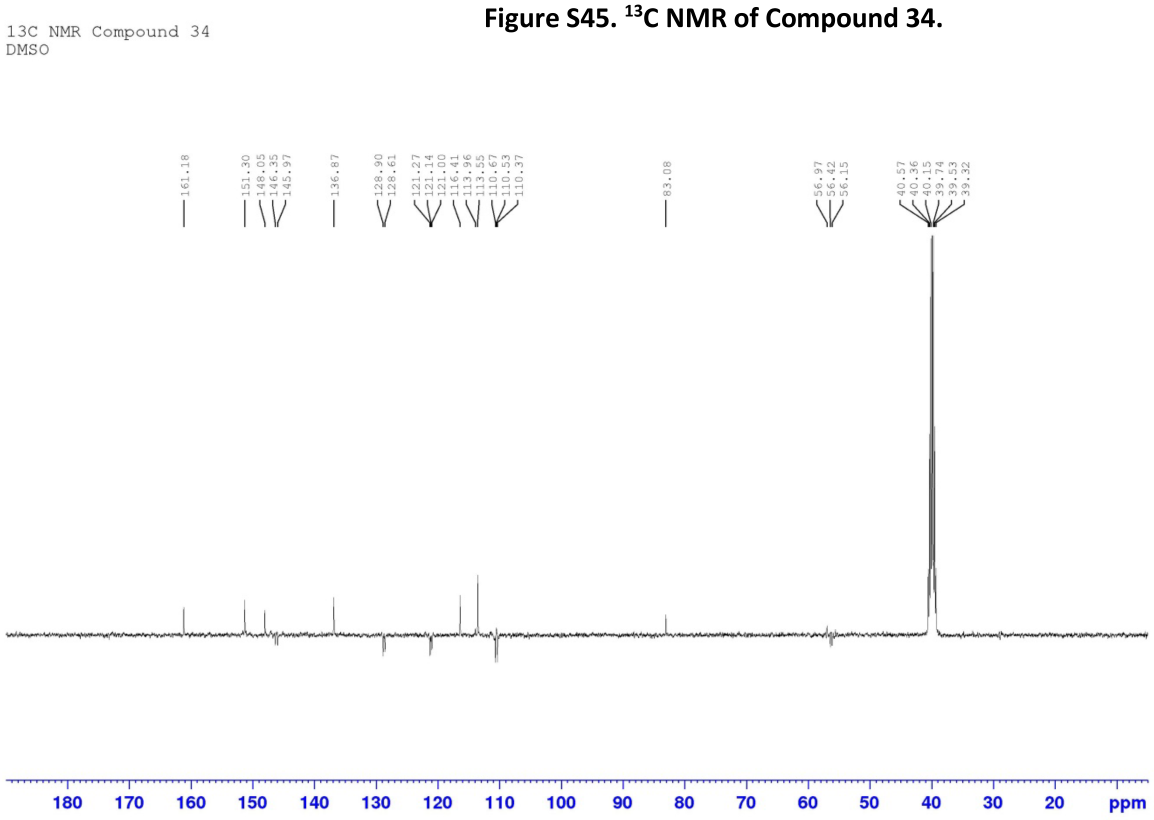

Figure S46. ^{1}H NMR of Compound 37.

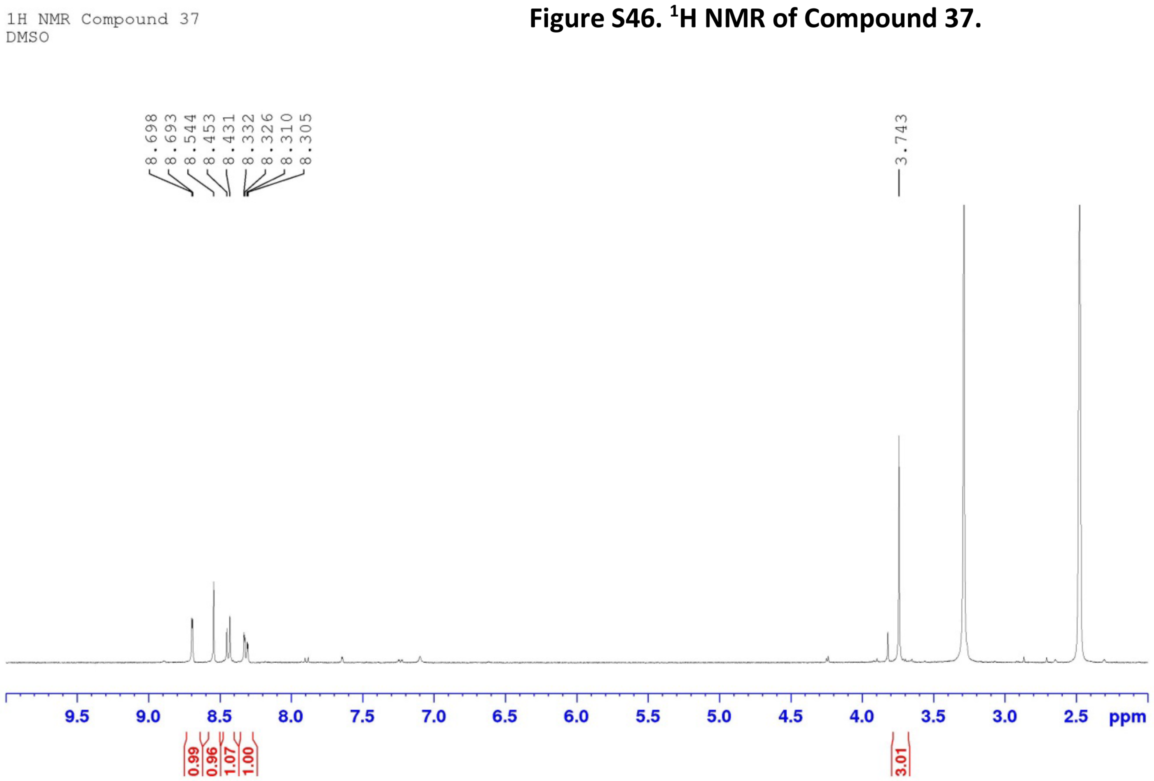

Figure S47. ^{13}C NMR of Compound 37.

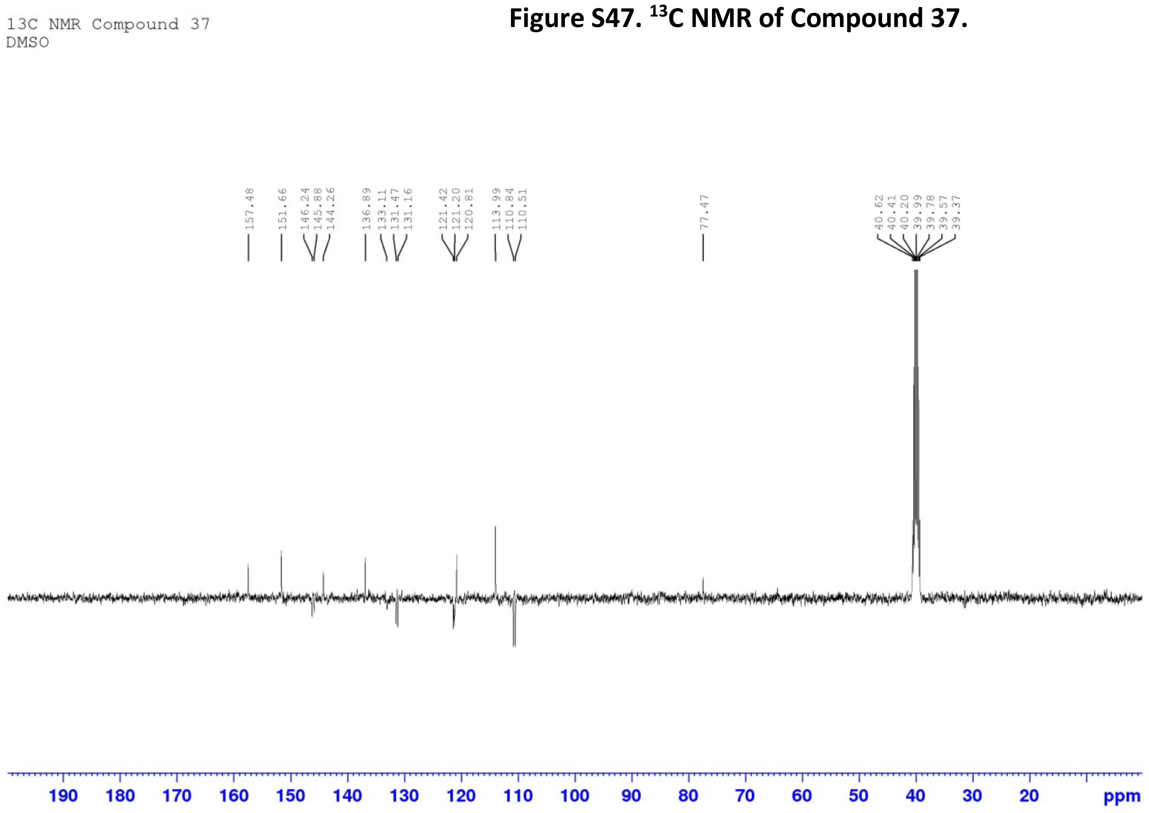

Figure S48. ^{1}H NMR of Compound 40.

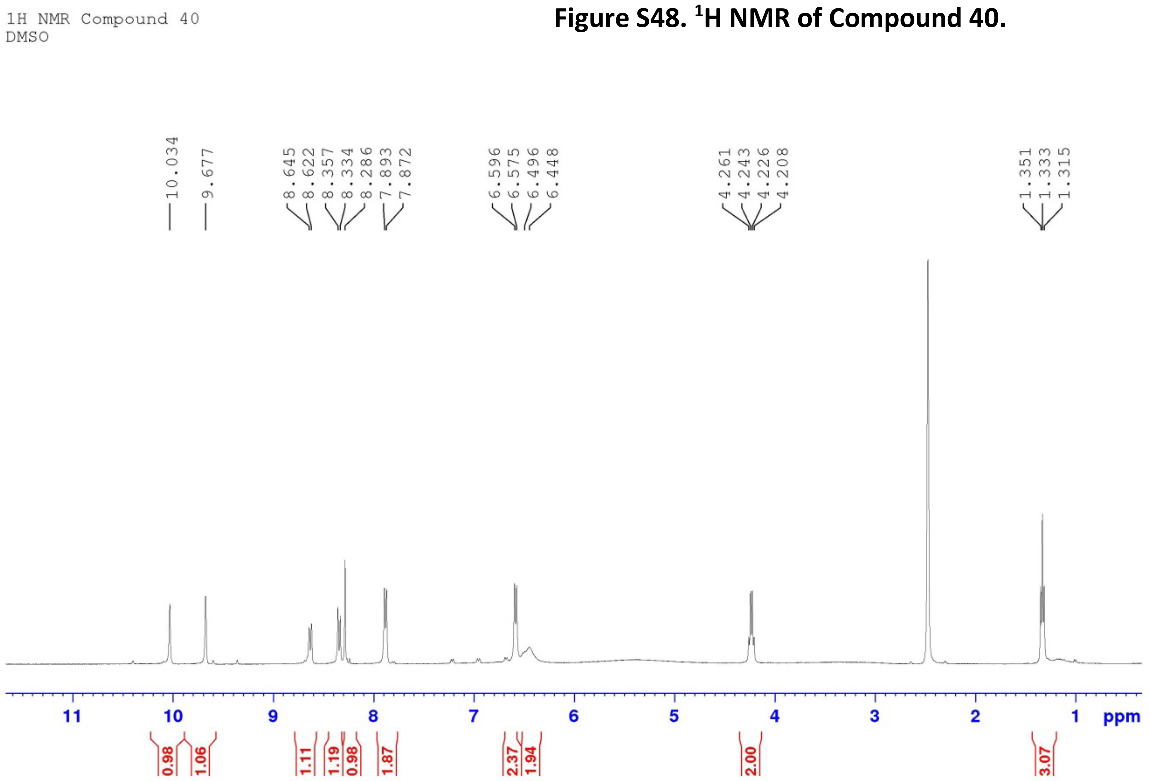

Figure S49. ^{1}H NMR of Compound 40 + D_2O.

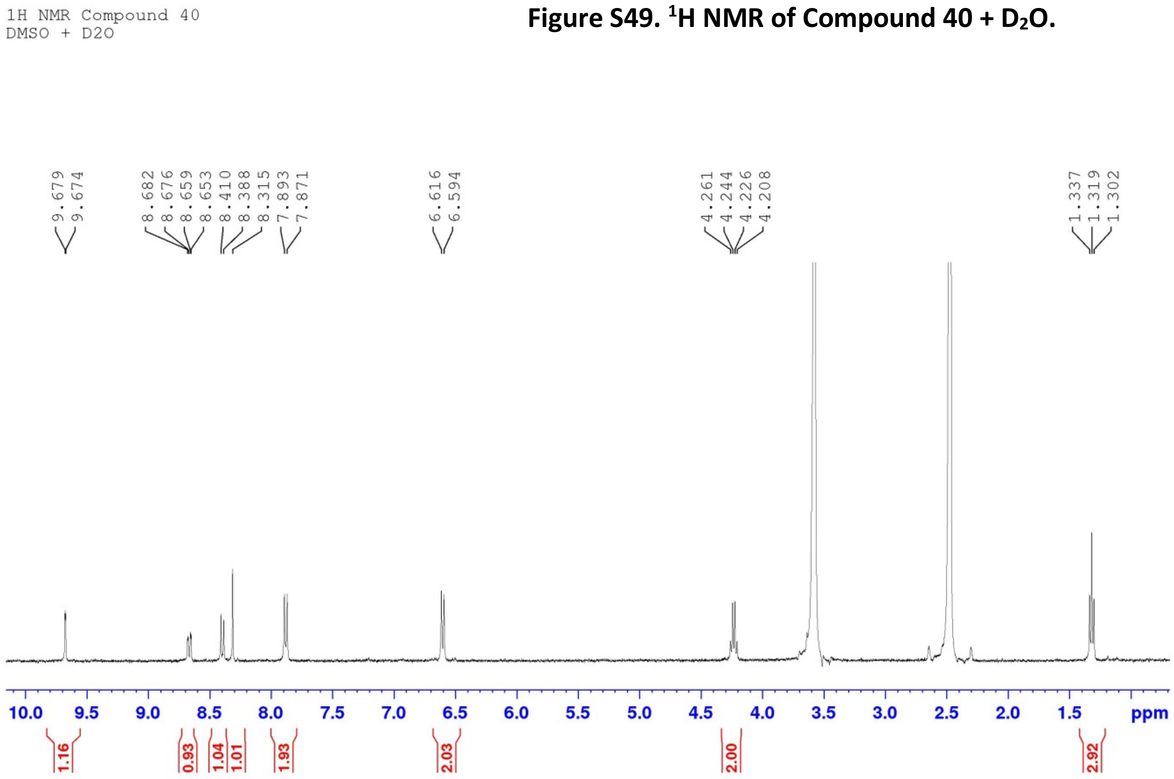

Figure S50. ^{13}C NMR of Compound 40.

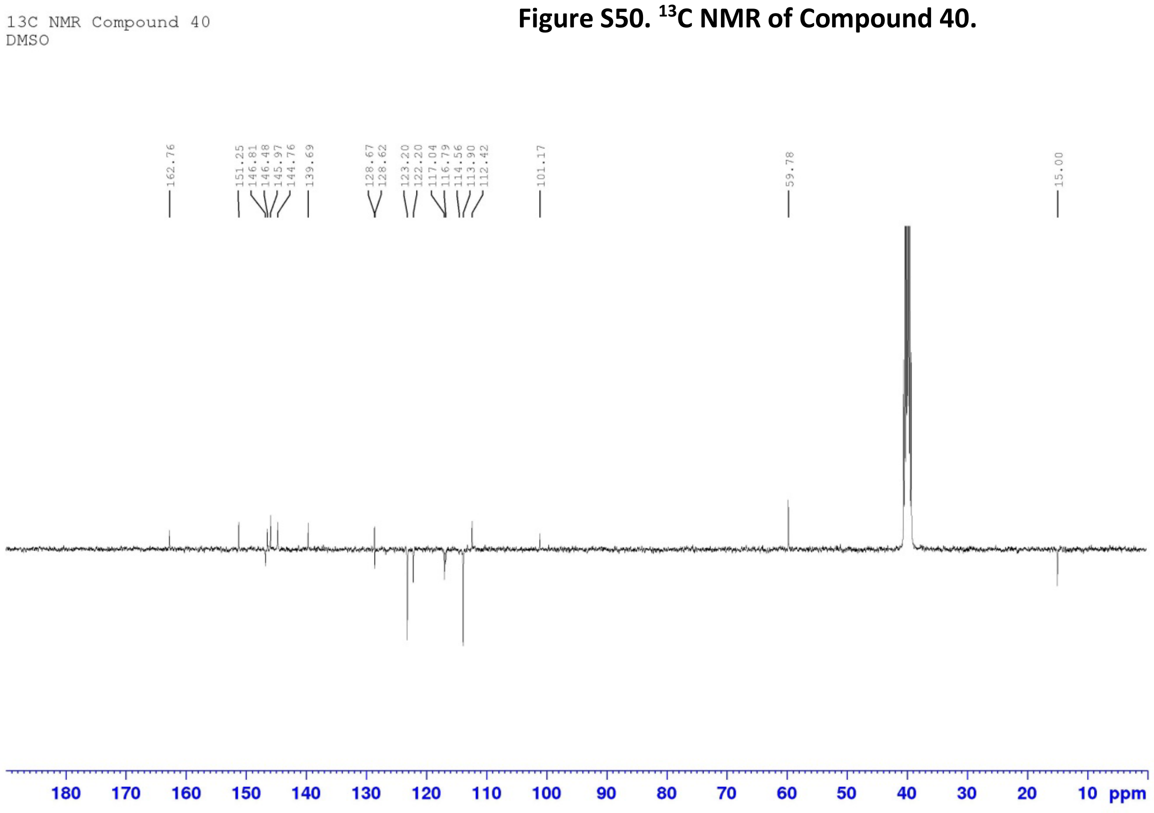

Figure S51. ^{1}H NMR of Compound 42d.

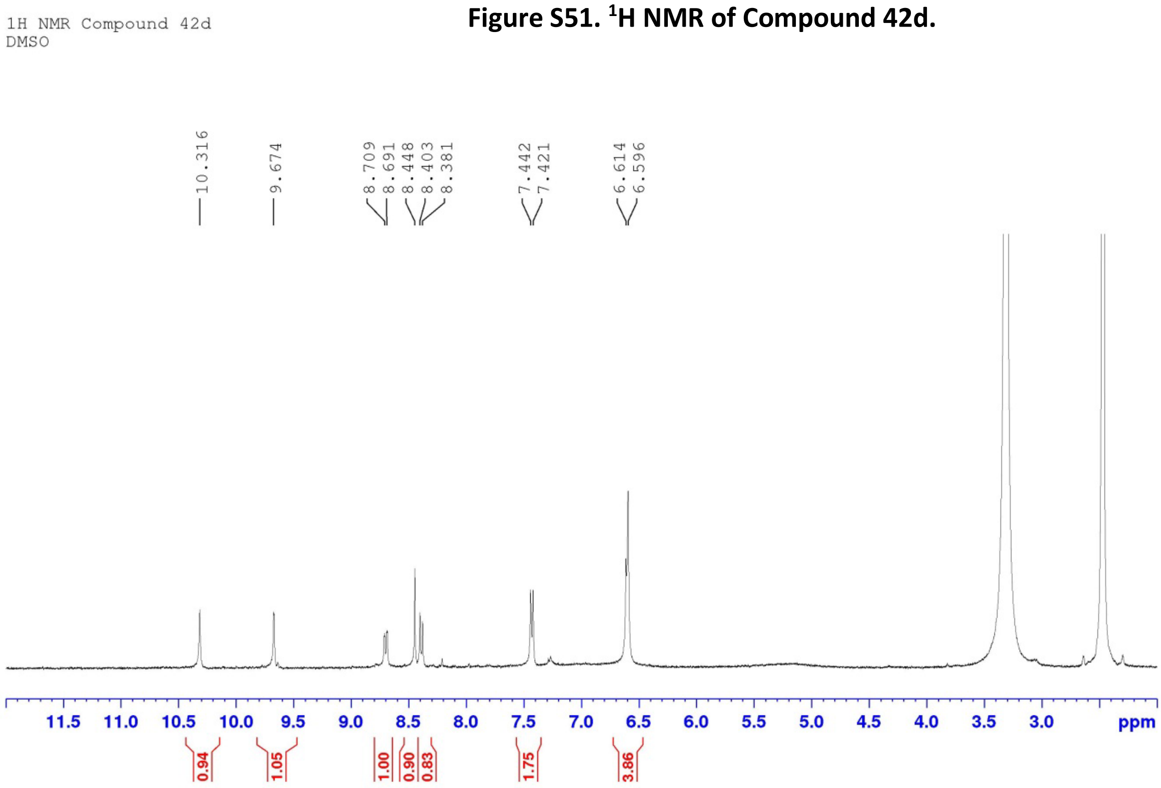

Figure S52. ^{1}H NMR of Compound 42d + D_2O.

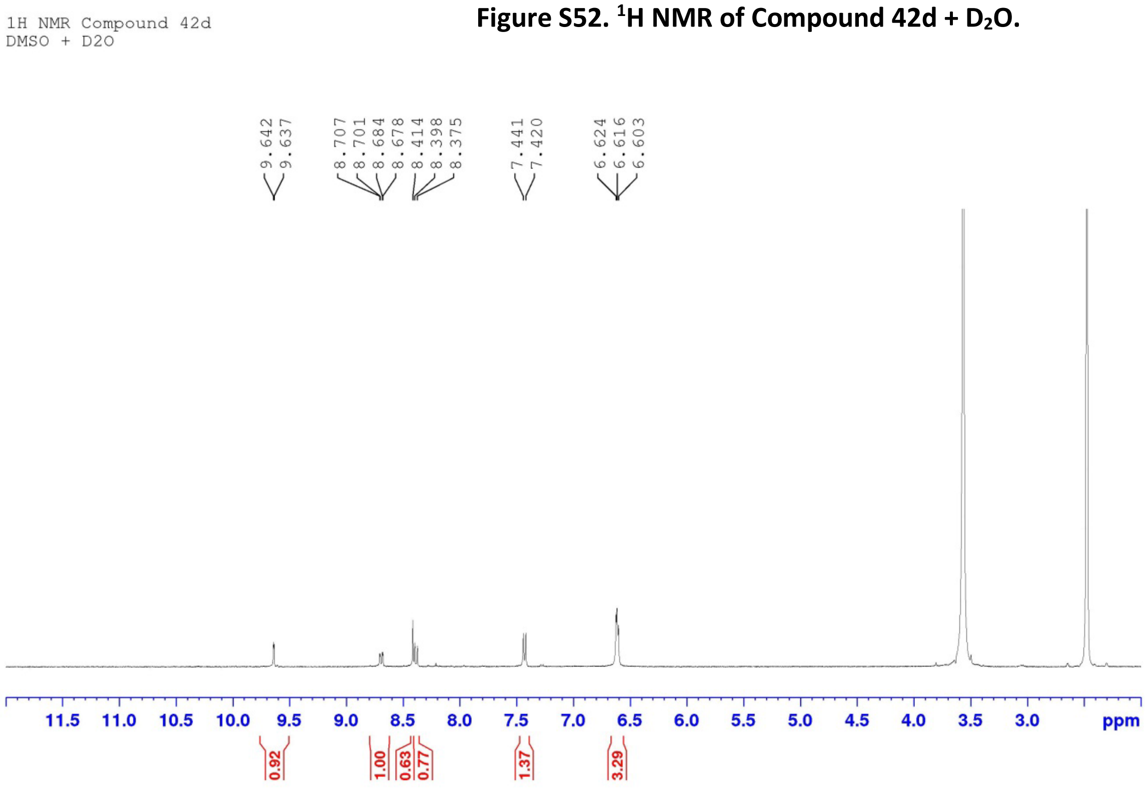

Figure S53. ^{13}C NMR of Compound 42d.

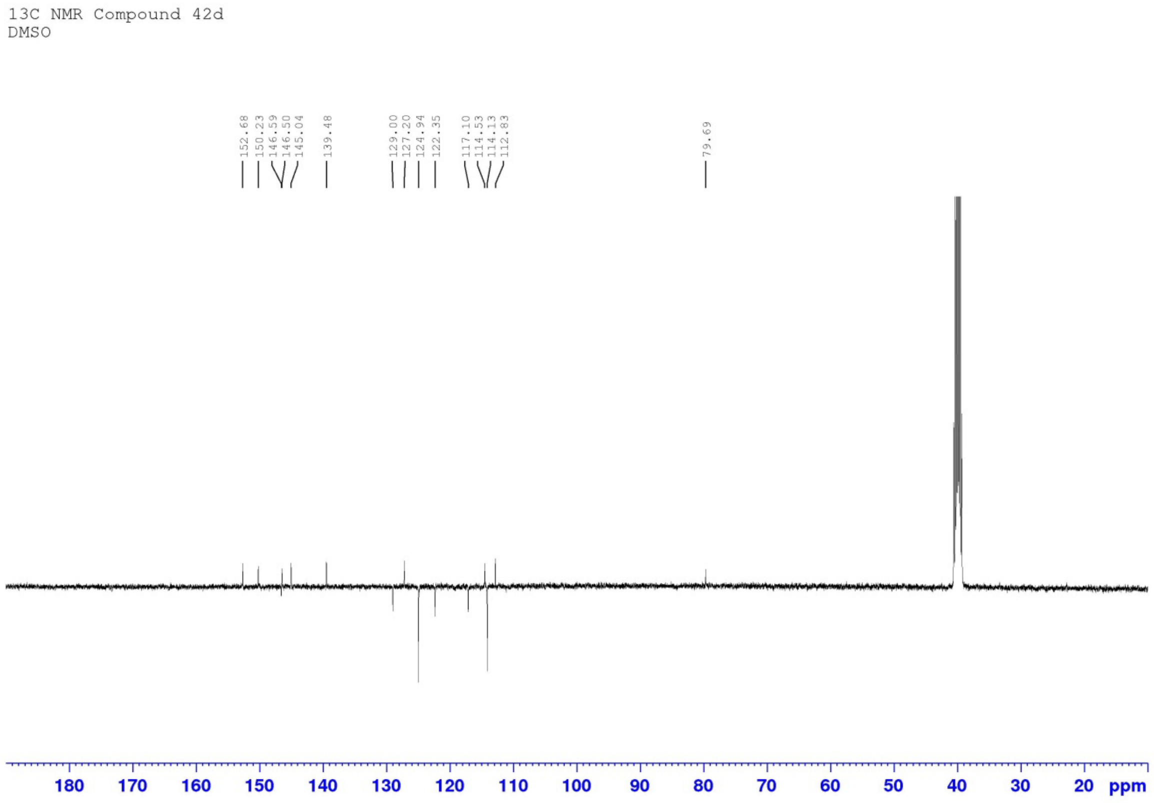

Figure S54. ^{1}H NMR of Compound 46a.

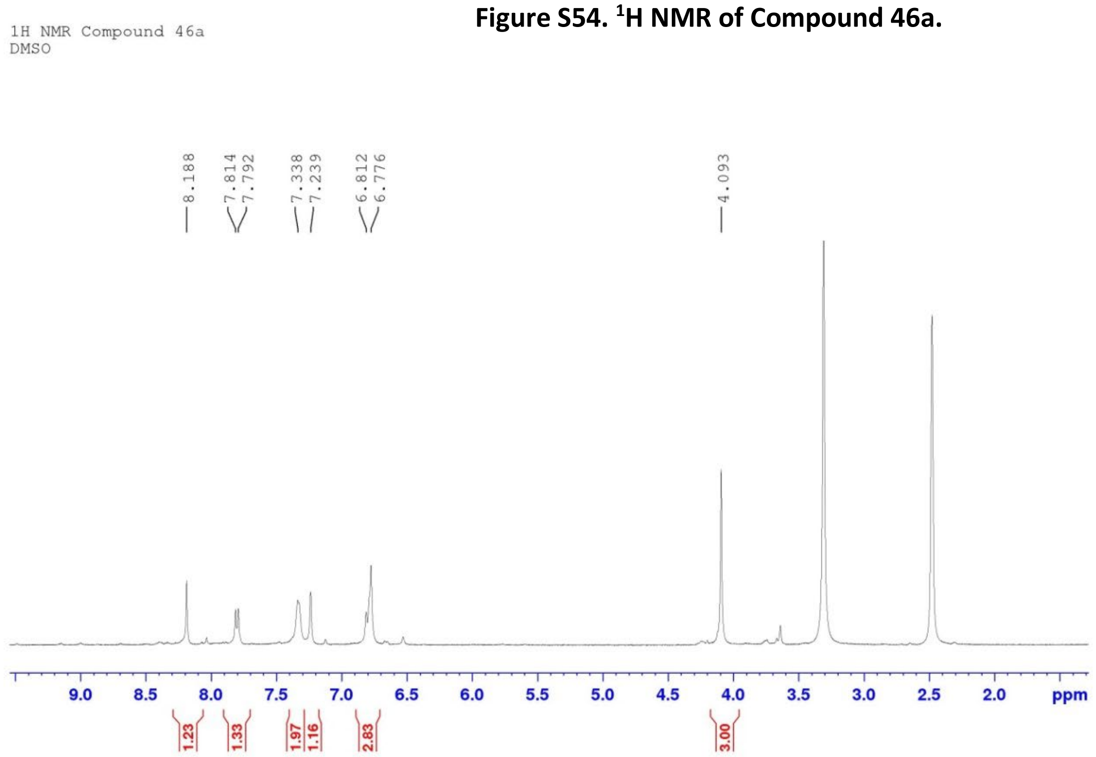

Figure S55. ^{13}C NMR of Compound 46a.

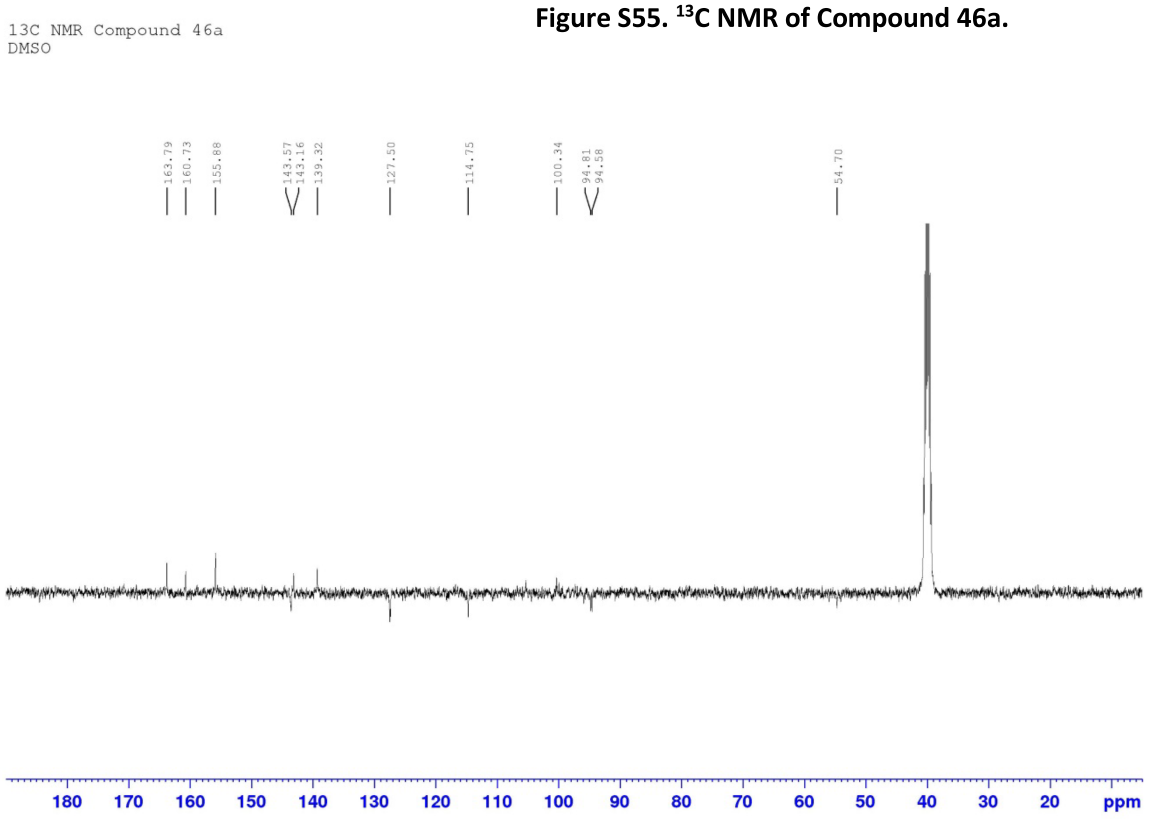

Figure S56. ^{1}H NMR of Compound 47.

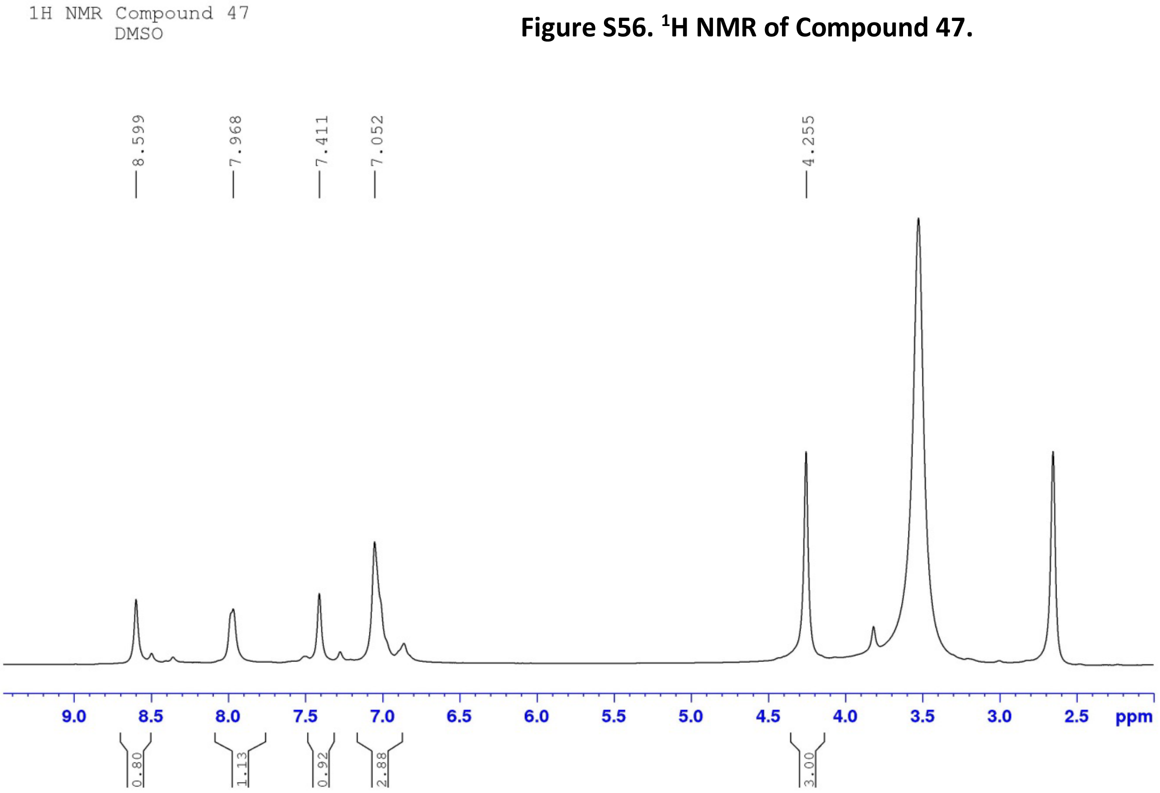

13C NMR Compound 47
DMSO

Figure S57. ^{13}C NMR of Compound 47.

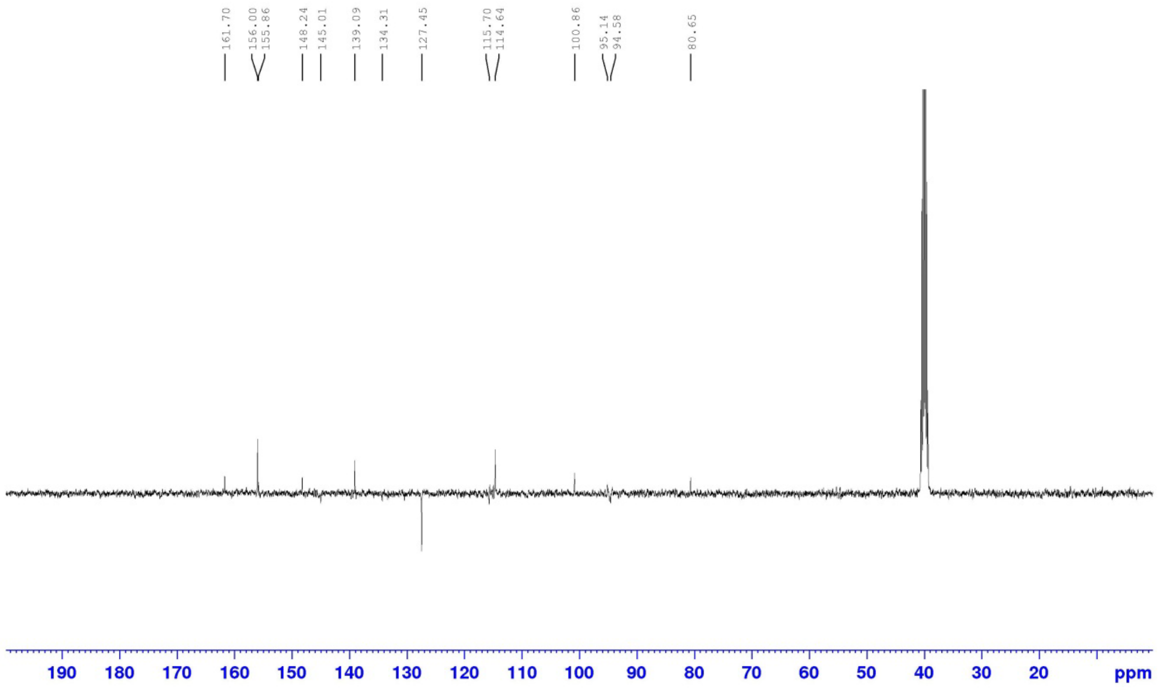

1H NMR Compound 49
DMSO

Figure S58. ^{1}H NMR of Compound 49.

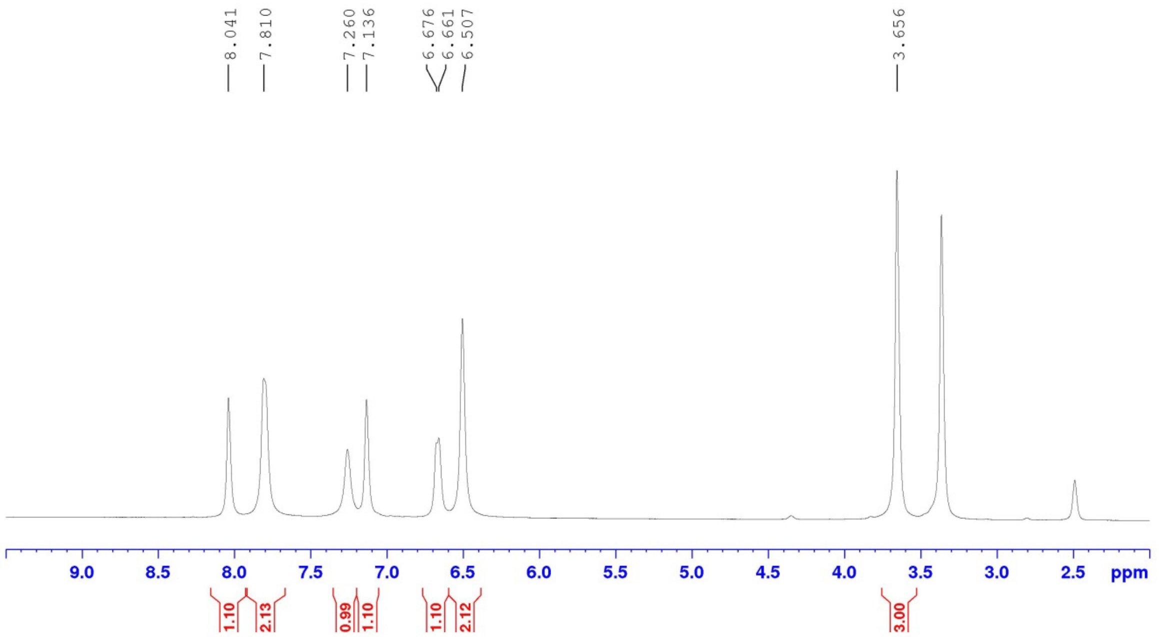

Figure S59. ^{13}C NMR of Compound 49.

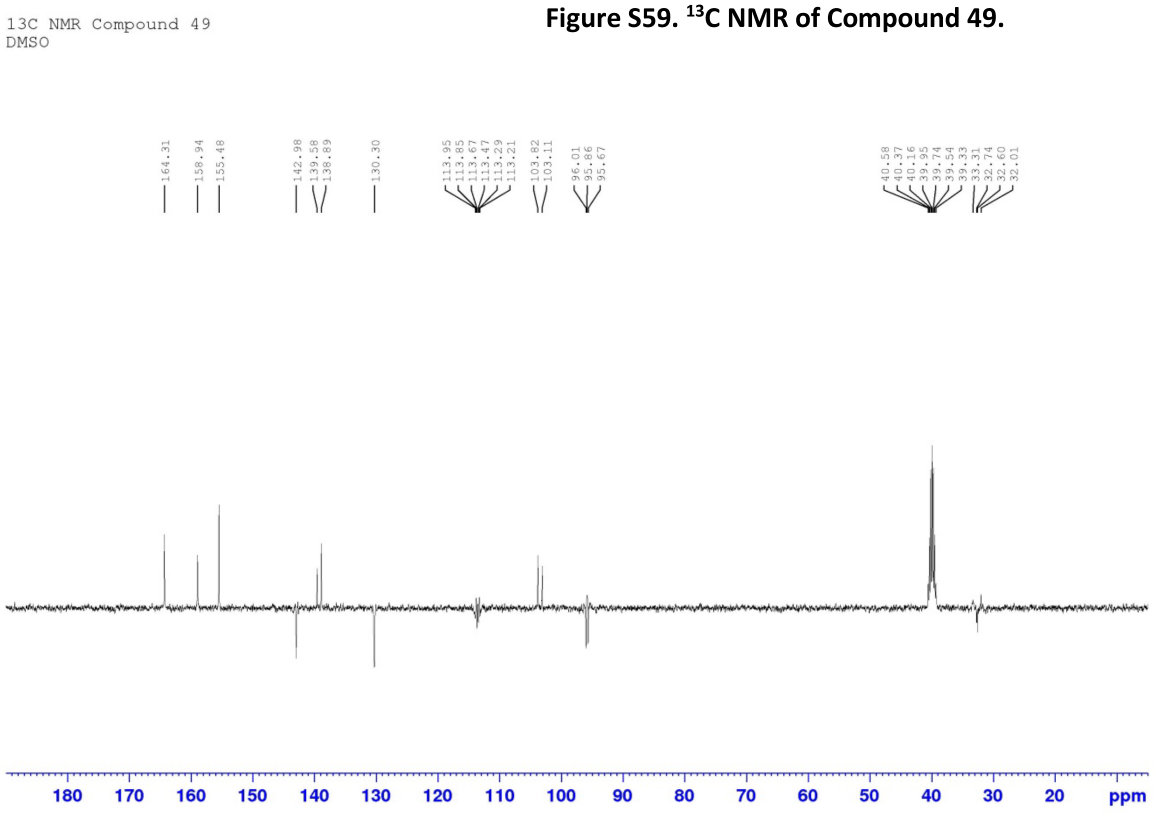

4. HSQC and HMBC of compounds 7b and 14c.

HSQC Compound 7b

Figure S60. HSQC of Compound 7b.

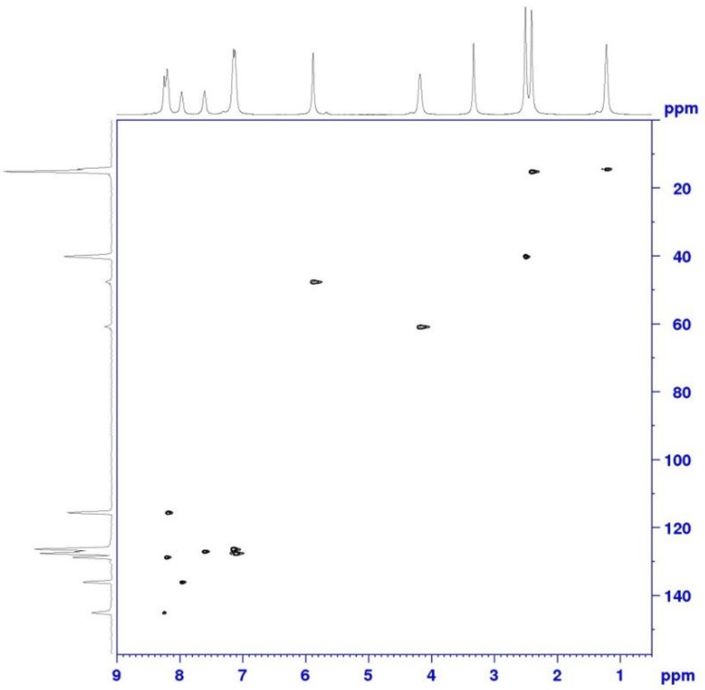

HMBC Compound 7b

Figure S61. HMBC of Compound 7b.

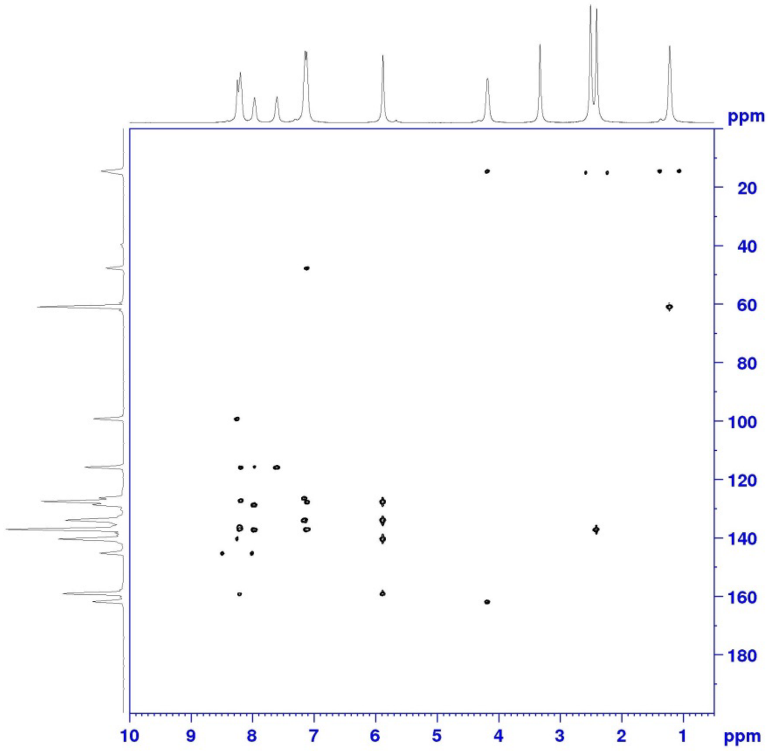

Figure S62. HSQC of Compound 14c.

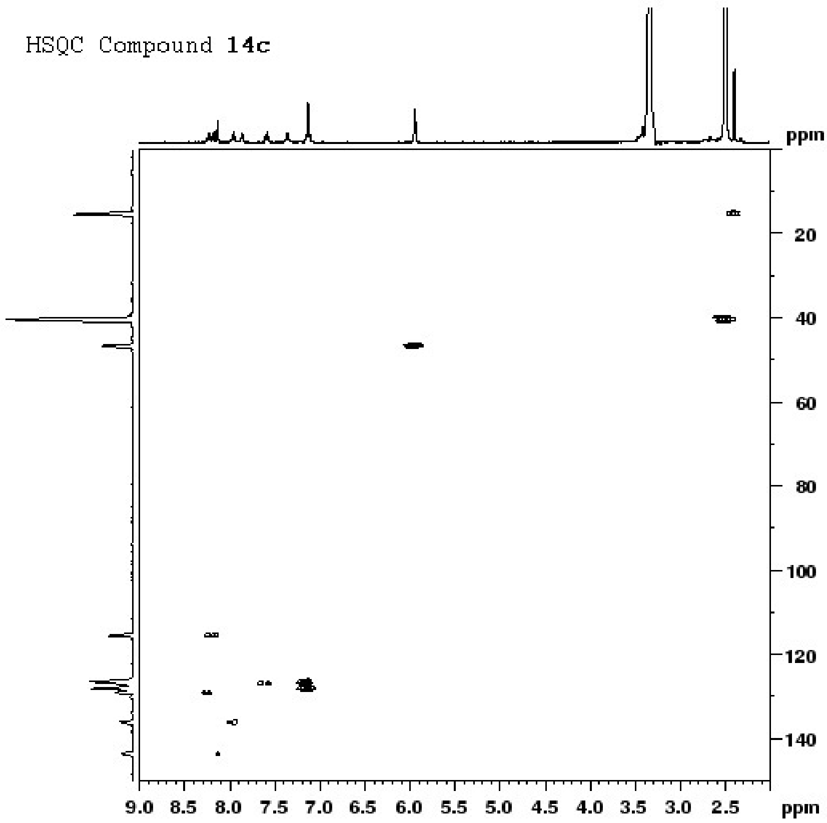

Figure S63. HMBC of Compound 14c.

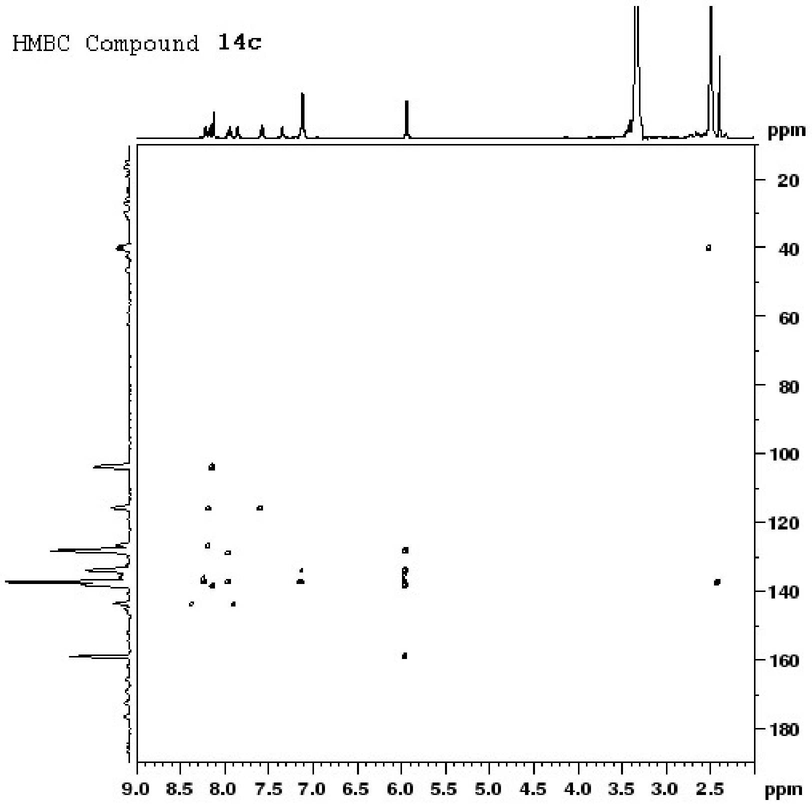

5. **Table S4.** Potential human protein targets for compounds **13i** and **16** identified by PharmMapper

	PDB	Targets for Compound 13i	Fit Score	Normalized Fit Score
**1**	**1JD0**	Carbonic anhydrase 12	2.999	0.9998
**2**	**1PME**	Mitogen-activated protein kinase 1 (ERK2)	2.976	0.9919
**3**	**3EKO**	Heat shock protein HSP 90-alpha	2.965	0.9883
**4**	**1YB1**	Estradiol 17-beta-dehydrogenase 11	2.949	0.983
**5**	**1MUO**	Serine/threonine-protein kinase 6 (PAK6)	2.949	0.983
**6**	**1GFW**	Caspase-3	2.925	0.975
**7**	**1PQ9**	Oxysterols receptor LXR-beta	2.923	0.9743
**8**	**1DVZ**	Transthyretin	2.923	0.9742
**9**	**1REU**	Bone morphogenetic protein 2	2.914	0.9714
**10**	**1ZUC**	Progesterone receptor	2.91	0.97
**11**	**1EUB**	Collagenase 3	2.906	0.9687
**12**	**2HD6**	Carbonic anhydrase 2	3.869	0.9673
**13**	**1W7H**	Mitogen-activated protein kinase 14 (p38**α**)	2.902	0.9672
**14**	**1CZM**	Carbonic anhydrase 1	2.891	0.9638
**15**	**2VTA**	Cell division protein kinase 2	2.877	0.959
**16**	**3HVC**	Mitogen-activated protein kinase 14 (p38**α**)	2.876	0.9588
**17**	**2OHP**	Beta-secretase 1	2.87	0.9568
**18**	**3EQM**	Cytochrome P450 19A1	2.865	0.9549
**19**	**1OZN**	Reticulon-4 receptor	2.856	0.9521
**20**	**2PIN**	Thyroid hormone receptor beta	2.853	0.951
**21**	**1YA8**	Liver carboxylesterase 1	2.846	0.9488
**22**	**1QKM**	Estrogen receptor beta	2.837	0.9458
**23**	**2PG2**	Kinesin-like protein KIF11	2.835	0.945
**24**	**1PMV**	Mitogen-activated protein kinase 10 (JNK3)	2.81	0.9368
**25**	**1UKI**	Mitogen-activated protein kinase 8 (JNK1)	2.807	0.9358
**26**	**1RS0**	Complement factor B	2.806	0.9354
**27**	**2P3G**	MAP kinase-activated protein kinase 2	2.799	0.9329
**28**	**2PIR**	Androgen receptor	2.794	0.9312
**29**	**2ITX**	Epidermal growth factor receptor	2.788	0.9293
**30**	**1YA3**	Mineralocorticoid receptor	3.702	0.9255

	PDB	Targets for Compound 16	Fit Score	Normalized Fit Score
1	1PME	Mitogen-activated protein kinase 1 (ERK2)	2.991	0.9972
2	1CZM	Carbonic anhydrase 1	2.973	0.9908
3	1W7H	Mitogen-activated protein kinase 14 (p38α)	2.969	0.9896
4	1JD0	Carbonic anhydrase 12	2.964	0.9879
5	3EKO	Heat shock protein HSP 90-alpha	2.951	0.9837
6	2PIN	Thyroid hormone receptor beta	2.946	0.9821
7	1LYW	Cathepsin D	2.937	0.979
8	2PG2	Kinesin-like protein KIF11	2.934	0.9779
9	1OKL	Carbonic anhydrase 2	2.93	0.9768
10	1REU	Bone morphogenetic protein 2	2.914	0.9714
11	1DVZ	Transthyretin	2.911	0.9704
12	1NO9	Prothrombin	2.902	0.9674
13	1TBB	cAMP-specific 3,5-cyclic phosphodiesterase 4D	3.86	0.9651
14	3HVC	Mitogen-activated protein kinase 14 (p38α)	2.893	0.9645
15	2OHP	Beta-secretase 1	2.886	0.9621
16	2VTA	Cell division protein kinase 2	2.873	0.9577
17	3GAM	Ribosyldihydronicotinamide dehydrogenase	2.866	0.9552
18	2BRG	Serine/threonine-protein kinase Chk1	2.862	0.9539

19	1YA8	Liver carboxylesterase 1	2.828	0.9428
20	1RS0	Complement factor B	2.823	0.941
21	1PMV	Mitogen-activated protein kinase 10 (JNK3)	2.811	0.9372
22	3EQM	Cytochrome P450 19A1	2.81	0.9367
23	1UKI	Mitogen-activated protein kinase 8 (JNK1)	2.809	0.9364
24	2A4Z	Phosphatidylinositol-4,5-bisphosphate 3-kinase catalytic subunit gamma isoform	2.796	0.9321
25	1QKM	Estrogen receptor beta	2.776	0.9252
26	2OCF	Estrogen receptor	2.77	0.9234
27	3DEJ	Caspase-3	2.743	0.9145
28	2PIR	Androgen receptor	2.738	0.9128
29	2OVM	Progesterone receptor	2.733	0.9108
30	2C3I	Serine/threonine-protein kinase Pim-1	2.725	0.9082

6. Molecular modelling for complexes of compounds 13i and 16 with JNK3 (PDB: 4WHZ) using AUTODOCK 4.1 program with further optimization using the amber99sb force field.

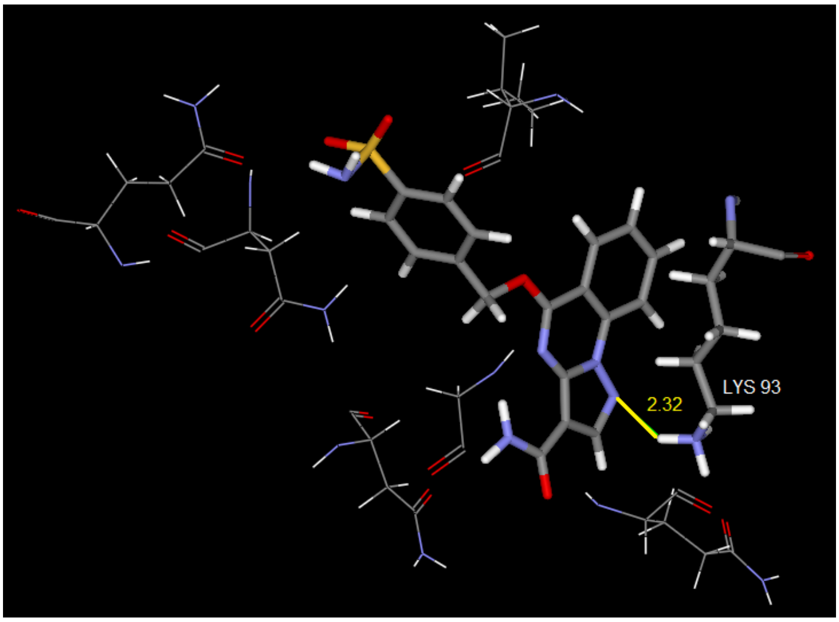

Figure S64. Complex of JNK3-13i.

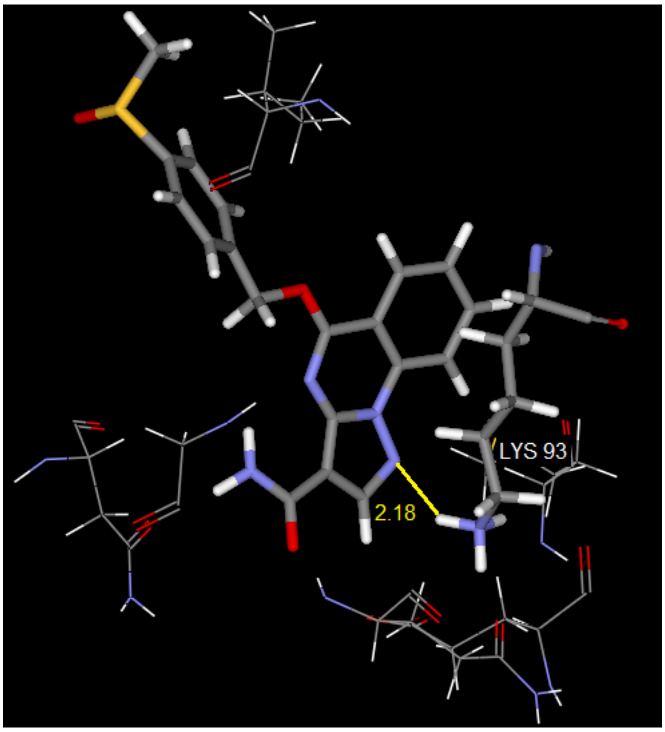

Figure S65. Complex of JNK3-16.

7. **Table S5.** H-bonding interactions obtained from the docking of ligands 13i and 16 into the binding sites of JNK3 (PDB: 4WHZ) obtained using AUTODOCK 4.1 program and geometry optimization with the amber99sb force-field.

**Ligand**	**Atom in the protein residue**	**Atom in the ligand**	**Distance (Å)**
**13i**	OE1 (Gln155)	H62	2.11
	OD1 (Asn194)	H86	1.91
	H (Gln75)	O28	2.91
	HZ2 (Lys93)	N18	2.32
	HZ3 (Lys93)	N18	2.98
	H (Asn152)	O4	2.47
**16**	OD1 (Asn194)	H88	1.93
	H (Gln75)	O54	2.19
	HZ2 (Lys93)	N22	2.18
